# Evolutionarily conserved short linear motifs drive actin filament binding

**DOI:** 10.1038/s41556-026-01979-9

**Published:** 2026-07-06

**Authors:** Themistoklis Paraschiakos, Biao Yuan, Michael Hecht-Bucher, Kostiantyn Sopelniak, Pasquale Cervero, Lisa Simon, Ksenija Zonjic, Dominic Eggers, Franziska Selle, Jing Li, Ali Biabani, Stefan Linder, Thomas C. Marlovits, Sabine Windhorst

**Affiliations:** 1 Department of Biochemistry and Signal Transduction, University Medical Center Hamburg–Eppendorf, Hamburg, Germany; 2 Institute of Microbial and Molecular Sciences, University Medical Center Hamburg–Eppendorf, Hamburg, Germany; 3 CSSB-Center for Structural Systems Biology, Hamburg, Germany; 4 Deutsches Elektronen Synchrotron (DESY), Hamburg, Germany; 5 Molecular Structural Biology, Helmholtz Center for Infection Research, Braunschweig, Germany; 6 Institute for Microbiology, Virology and Hygiene, University Medical Center Hamburg–Eppendorf, Hamburg, Germany; 7 Core Facility Mass Spectrometric Proteomics, University Medical Center Hamburg–Eppendorf, Hamburg, Germany

**Keywords:** Proteins, Cellular signalling networks, Actin, Cryoelectron microscopy

## Abstract

Regulation of the actin cytoskeleton by actin-binding proteins is essential for cellular homeostasis, and the mode of actin binding determines the activity of actin-binding proteins. Here we identify a ‘short linear actin filament-binding motif’ (SFM) based on the cryo-electron microscopy structure of the ITPKA–actin filament complex. Using the computational pipeline SLiMFold, we discovered 103 human proteins containing SFMs with diverse cellular roles. Phylogenetic analysis suggests that SFMs arose de novo and are conserved across eukaryotes, exhibiting actin filament-binding affinities of 2–12 µM. Critical residues mediating binding and modulating affinity were defined, and the cryo-electron microscopy structures of two SFM–actin filament complexes revealed that SFM binding decreases actin-filament stiffness. These findings indicate that SFMs regulate actin-filament conformation and serve as anchoring modules that connect actin dynamics to a broad variety of cellular functions, providing a framework for understanding the actin-associated roles of numerous proteins.

## Abstract (web summary)

Paraschiakos, Yuan et al., using a computational pipeline, identify the short linear actin filament-binding motif (SFM) as an evolutionarily conserved actin filament-binding motif present in 103 human proteins.

## Main

Actin filaments form a dynamic cytoskeletal framework in eukaryotic cells, driving essential processes such as cell motility, division and morphogenesis. These processes are orchestrated by different actin-binding proteins (ABPs) along with signalling and scaffolding proteins^1^. The ABPs assist in polymerization, nucleation, capping, severing, crosslinking and bundling of actin filaments, and are required to coordinate actin turnover. Their impact on actin dynamics depends on the affinity of the actin-binding domain (ABD) and, in most cases, on cellular stimulation^2^. Some ABPs are inactive in resting cells and many of them are activated by small GTPases. Failures in the regulation or expression of ABPs as well as mutations can lead to severe diseases, such as neuronal and immunological disorders and cancer^3–7^.

Among the ABPs, many conserved domains and motifs have been identified, on which ABP-families with similar functions were defined. Supplementary Table 1 illustrates how the remarkable variety of motifs and domains matches the diversity of cellular actin functions. Despite this diversity, many ABPs, such as cofilin-1, plastins, myosins and gelsolin, bind to a hydrophobic cleft between actin subdomains (SDs) 1 and 3 (ref. ^8^). Here the binding modus and/or the affinity is different, resulting in competition of the ABPs with each other^9^.

Alongside globular domains, such as that of cofilin, plastins, myosins and gelsolin, unstructured regions play essential roles in many dynamic cellular processes^10–19^. Among these, the most common functional modules are short amino acid stretches known as short linear motifs (SLiMs)^20–23^. The actin-binding Wiskott–Aldrich homology 2 (WH2) motif is a prominent example of a SLiM; structure elucidation revealed that it binds to globular actin at the hydrophobic cleft between actin SD1 and SD3 (ref. ^24^). This motif is found in many ABPs, where it stabilizes actin monomers in a polymerization-competent conformation. Wiskott–Aldrich syndrome protein (WASP) and WASP-family verprolin-homologous protein (WAVE2) activate the actin-related protein 2 and 3 complex (Arp2/3) nucleation factors and facilitate the delivery of actin monomers, whereas proteins such as Spire and Cordon-Bleu, containing tandem WH2 domains, are capable of sequestering, nucleating and severing actin filaments^25^. Missense mutations located inside the WH2 motif are associated with diseases, for example, the R543L^26^ and L550F^27^ mutations in human Leiomodin-3 (LMOD3) are associated with nemaline myopathy 10. Moreover, WH2 motifs can be part of bacterial effector proteins, often leading to restructuring of the actin cytoskeleton of the host cell and promoting either efficient colonization (VopL and VopF) or bacterial motility during infection (Sca2 and RickA)^28–31^.

Here we identify a short linear actin filament-binding motif (SFM) that mediates actin filament binding in diverse human proteins. Comparative sequence analyses indicate that the SFM is under purifying selection and acts as a molecular anchor, targeting various cytosolic proteins to the actin cytoskeleton. Notably, our findings not only unify unclassified sequences but also enable the prediction of undiscovered SFM-containing proteins. These observations point to a broader role for the SFM in cytoskeletal organization.

## Results

### Structural insight into inositol-trisphosphate 3-kinase A in actin filament binding

Many ABPs contain uncharacterized domains responsible for actin filament binding, including the human inositol-trisphosphate 3-kinase A (ITPKA)^32–36^. ITPKA constitutively binds to actin filaments and, due to homodimer formation, exhibits actin filament bundling activity^36^. ITPKA helps regulate the postsynaptic actin architecture of hippocampal neurons and is also expressed in malignant tumour cells, where its actin bundling activity induces the formation of cellular protrusions essential for invasion^34,37^.

**Fig. 1:**
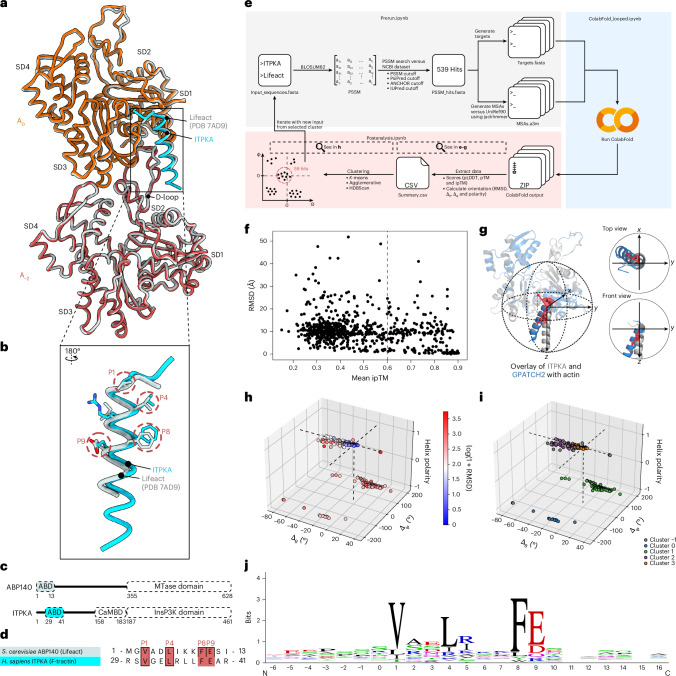
Structural alignment of ITPKA^Arg28–Ala49^ and Lifeact points towards an actin filament-binding motif, prompting proteome-wide discovery using the SLiMFold pipeline. **a**, Structural superimposition of the ITPKA^Arg28–Ala49^–actin complex (the two actin subunits, A_0_ and A_−2_, are shown) with the Lifeact–actin structure (PDB: 7AD9). **b**, A 180° rotation of the helices reveals which residues interact with actin filaments. Conserved side chains are represented as sticks and labelled P1, P4, P8 and P9. **c**, Domain architecture of *Saccharomyces cerevisiae* ABP140 and *Homo sapiens* ITPKA. ABP140 comprises an N-terminal ABD and a C-terminal methyltransferase (MTase) domain. ITPKA features an N-terminal ABD, a central calmodulin-binding domain (CaMBD) and a C-terminal inositol 1,4,5-trisphosphate 3-kinase (IP3K) domain. **d**, Sequence comparison. The conserved positions P1, P4, P8 and P9 are labelled in red. **e**, SLiMFold pipeline overview. The SLiMFold pipeline starts with the hypothesized SLiMs of the Lifeact and ITPKA^Arg28–Ala49^ sequence, on which basis a PSSM was calculated to identify motif hits from NCBI databases, which were filtered by PSSM scores, IUPRED, ANCHOR and PSIPRED. This yielded 539 hits in the first iteration. Thereafter, MSAs were calculated locally using UniRef90 with modified jackhmmer filters, followed by multimer predictions on ColabFold. The predicted structures were analysed by extracting AlphaFold2 scores, computing RMSD and angle metrics (θ, φ). These data were clustered to identify 59 new sequences that carry the suspected motif. **f**–**i**, Detailed illustration of calculated metadata from **e**. **f**, Scatterplot of predicted peptide–actin complexes after three iterations of the SLiMFold pipeline. For each complex, the mean ipTM values were calculated from the top-three AlphaFold2 Multimer models. Mean ipTM scores are plotted against RMSD relative to ITPKA^Arg28–Ala49^ to indicate structural similarities of actin-binding modes for the identified SLiM–actin interactions. A red dotted line at ipTM = 0.6 marks the threshold below which interactions are unreliable. **g**, Helix orientation was assessed by comparing the ∆φ and ∆θ angles in a polar coordinate system after superimposing each complex onto the ITPKA^Arg28–Ala49^–actin reference. **h**, A three-dimensional plot of ∆φ, ∆θ and helix polarity (1, same direction; −1, opposite) maps each predicted peptide, with data points coloured according to the log-transformed RMSD (Å) values. Angles near zero indicate an orientation similar to that of ITPKA^Arg28–Ala49^. **i**, The hdbscan algorithm clusters these data points by grouping peptides according to their orientation and RMSD. Outliers are assigned to Cluster –1. Cluster 3 contains helices most closely matching ITPKA^Arg28–Ala49^. **j**, Sequence logo depicting positional conservation among the 59 identified peptides confirms robust conservation at positions P1, P4, P8 and P9 (strongest at P1 and P8). Source data

**Extended Data Fig. 1:**
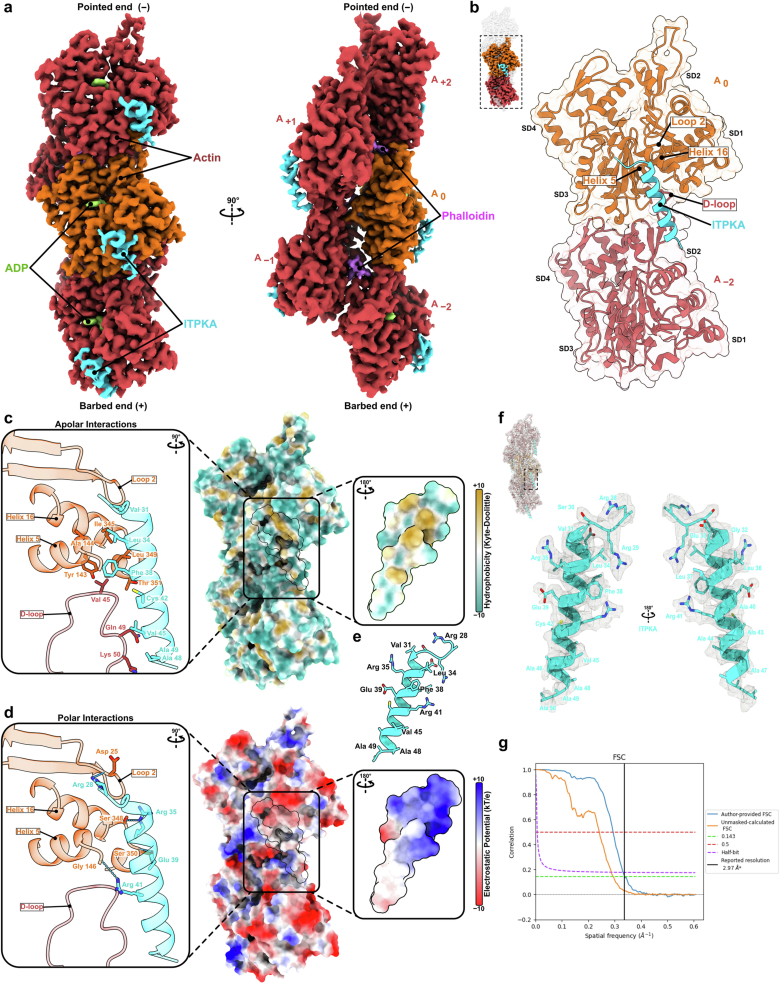
Cryo-EM structure of the ITPKA–actin complex. **a**,The electron density map illustrates the ITPKA–actin complex. A single ITPKA helix of residue 28–49 (cyan) is resolved, forming the primary binding interface with actin. Actin subunits are displayed with the central subunit in orange and surrounding subunits in red. Phalloidin is shown in purple, and ADP in light. **b**, A cartoon representation of the ITPKA–actin complex shows ITPKA bridging two adjacent actin subunits. Key interaction regions in actin helix 5 (AA 137–145), helix 16 (AA 338–348), loop 2 (AA 21–28), the D-loop (AA 38-51) and actin subdomains SD1–SD4 are labelled. **c**, Apolar interactions between ITPKA and actin are depicted by surface colour-coding for hydrophobicity (yellow = hydrophobic). Left panel: Zoomed-in view and a 90° rotation highlight interacting residues displayed as sticks. Right panel: Zoomed-in view and a 180° rotation; here hydrophobicity scores are shown (low: cyan, high: yellow). **d**, Polar contacts between ITPKA and actin are shown via electrostatic potential mapping on actin (negative: red, positive: blue). Left panel: Zoomed-in view and a 90° rotation highlight interacting residues displayed as sticks. Right panel: Zoomed-in view and a 180° rotation, here electrostatic scores are shown (negative: red, positive: blue). **e**, The binding helix of ITPKA is separately depicted as a cartoon with side chains shown as sticks. **f**, Left, the full actin filament model is shown with a semi-transparent molecular surface so that the underlying cartoon representation remains visible; boxes mark the bound ITPKA. Right, zoom-in views show the ITPKA helix with its corresponding cryo-EM density (grey mesh) in two orientations related by a 180° rotation around the helix axis. **g**, Map–model Fourier shell correlation (FSC) curves. Shown are FSCs between the refined model and the unsharpened half-maps, with standard 0.5 and 0.143 criteria indicated.

To investigate the molecular basis of ITPKA for actin filament binding, we solved the cryo-electron microscopy (cryo-EM) structure of the ITPKA–actin filament complex at a resolution of 2.97 Å. In this structure, the ABD of ITPKA is clearly visible, whereas the catalytic domain and the linker connecting it to the ABD were not resolved (Fig. 1a, Extended Data Fig. 1 and Supplementary Table 2), confirming that the carboxy-terminal InsP_3_Kinase domain is not directly involved in actin filament binding. The amino acid residues Arg28–Ala49 bind to the hydrophobic cleft between SD1 and SD3, and also contact the DNase-binding loop (D-loop) of the adjacent actin subunit (A_−__2_ ; Fig. 1a and Extended Data Fig. 1). In parallel to us, the Belyy group resolved the cryo-EM structure of actin filament bound by F-tractin, which represents the core F-actin-binding domain (ABD) of ITPKA and is used as actin filament marker for live-cell imaging^38,39^. This structure matches our findings of the actin filament-binding mode^38^.

Collectively, we found that the core ABD of ITPKA only comprises 21 amino acid residues folded into an alpha helix. Interestingly, this peptide exhibits the characteristics of a SLiM and its structure resembles that of the actin filament probe Lifeact^9,38,40^. Based on this consideration, we performed a structural alignment of ITPKA^Arg28–Ala49^ with Lifeact (PDB: 7BTE and 7AD9) and found highly similar helix positioning, including the aligned positions Val (P1), Leu (P4), Phe (P8) and Glu (P9; Fig. 1b–d).

**Extended Data Fig. 2:**
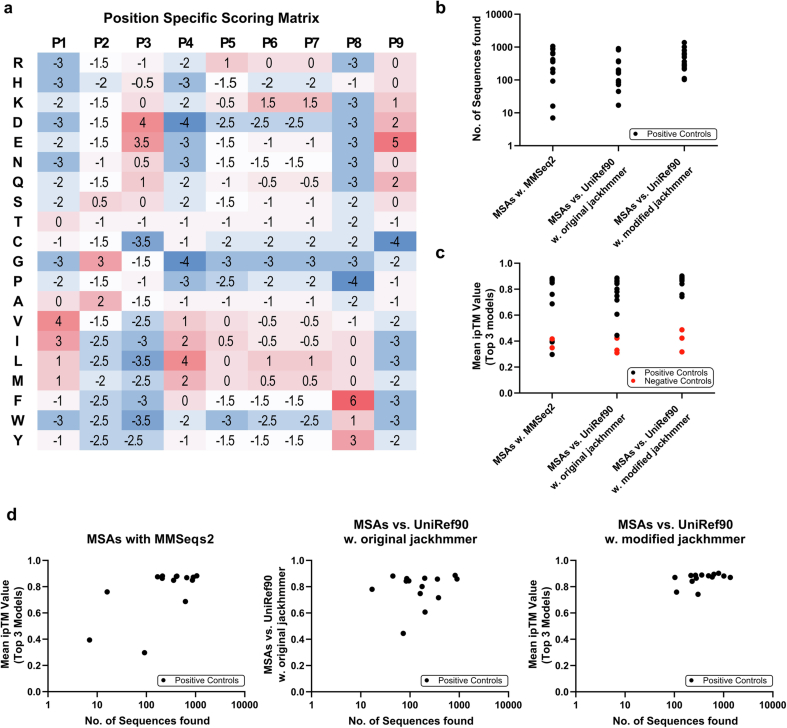
Position-specific scoring matrix derived from the two validated motif sequences, ITPKA and Lifeact and optimization of AlphaFold2 parameters for the SLiMFold pipeline. **a**, Nine positions (P1–P9) were aligned (Fig. 2c) and scored using a BLOSUM62-based approach, generating the PSSM shown. In this heat map, rows are grouped by amino acid type: positively charged (+), negatively charged (−), polar (P), unique (U), non-polar (NP) and aromatic (A) residues; and the position-wise scores are colour-coded (red = favourable, blue = unfavourable). Higher (positive) scores indicate preferential substitutions, whereas lower (negative) values suggest disfavoured residues at a given position. This matrix, which reflects sequence conservation in the ITPKA and Lifeact SLiMs, provided the starting point for screening and iterative refinement in the SLiMFold pipeline (Fig. 2 and Methods). **b**, Comparison of the number of sequences identified (*y* axis, log scale) under different multiple sequence alignment (MSA) algorithms: MMSeq2, UniRef90 with original jackhmmer parameters, and UniRef90 with modified jackhmmer parameters. Positive controls (black) are known SLiM-containing peptides validated in this work; negative controls (red) are random amino acid sequences. **c**, Mean ipTM scores (top 3 ranked of 20 predicted structures) for positive controls (black) versus negative controls (red) across the same MSA methods. Positive controls run with the modified jackhmmer settings exhibit higher mean ipTM values compared with MMSeq2 or the original jackhmmer, suggesting improved interface prediction accuracy with the modified parameters. **d**, Scatter plots show the relationship between the number of sequences identified (*x* axis, log scale) and the mean ipTM values (*y* axis) for each MSA approach. Source data

This discovery led us to propose the actin filament-binding SLiM VxxLxxxFE (Fig. 1d). To determine whether other human proteins contain this motif, we developed the computational pipeline SLiMFold (Fig. 1e), integrating a variety of bioinformatic tools for identification, filtering and structure prediction of putative SLiMs. First, we derived a position-specific scoring matrix (PSSM) from the aligned sequences of ITPKA^Arg28–Ala49^and Lifeact (Fig. 1e and Extended Data Fig. 2), and used it to screen against the NCBI human protein database. The PSSM scores each motif position so that not only exact matches but also closely related amino acids (for example, Val, Leu and Ile or Phe and Tyr) are accepted as compatible substitutions. Next, we applied PSIPRED^41^, ANCHOR^42^ and IUPred^43^ cutoffs to further filter candidates containing this SLiM (Fig. 1e(top)). For each of these 539 hits, a multiple sequence alignment (MSA) was performed by running jackhmmer^44^ against the UniRef90 database with optimized parameters to improve speed and coverage (Extended Data Fig. 2).

These MSAs were used as input for customized ColabFold^45^ runs employing AlphaFold2 Multimer v3 to predict the structures of all peptide–actin complexes. Models were evaluated using the predicted local distance difference test (pLDDT), predicted template modelling score (pTM) and interface pTM score (ipTM). Candidates with a mean ipTM score of <0.6 were excluded from further analysis (Fig. 1e(bottom),f).

**Extended Data Fig. 3:**
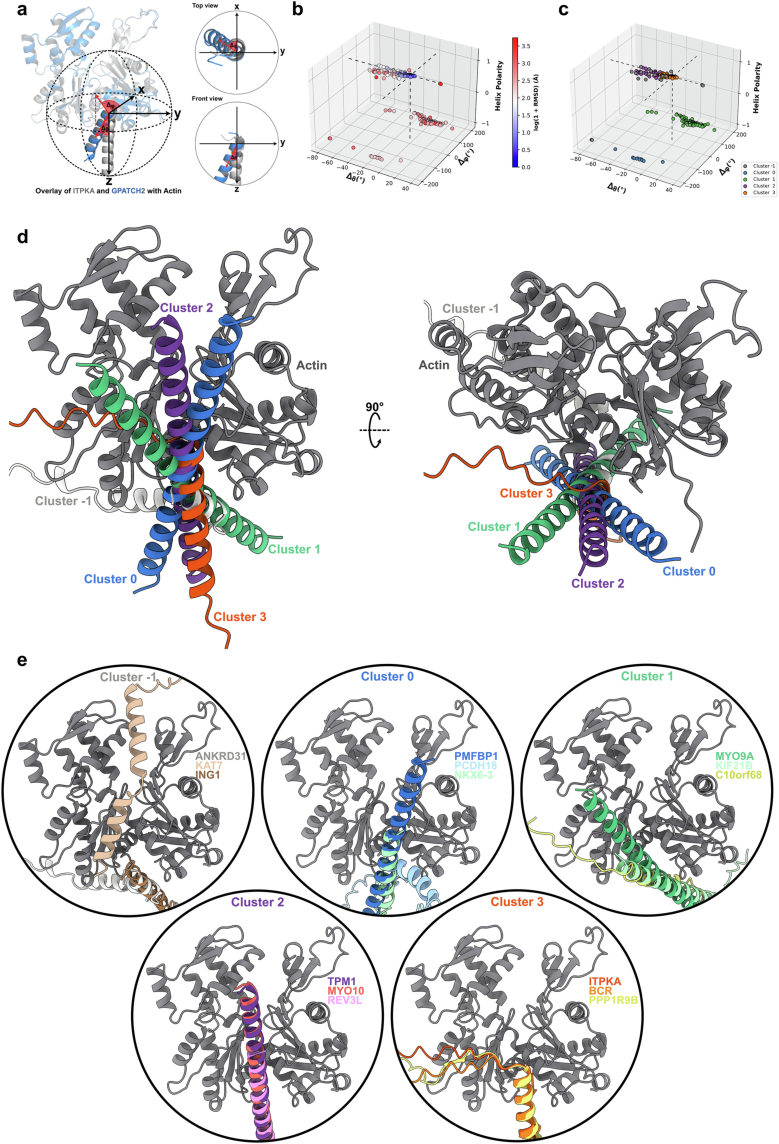
Orientation-based clustering of SLiMFold hits on actin. **a**, Reference frame used to compare peptide orientations. Actin is shown in grey; example helices (ITPKA and GPATCH2) are overlaid to illustrate the alignment procedure. After superposing each peptide–actin model onto the ITPKA–actin reference, helix direction and position are described by two angles: Δφ (rotation around the filament/barbed-end axis) and Δθ (tilt)—and a polarity term (+1, same N → C direction as ITPKA; −1, opposite). **b**, 3D scatter of all predicted complexes in (Δφ, Δθ, polarity) space, coloured by RMSD. **c**, The same embedding coloured by hdbscan cluster assignment. **d**, Cluster prototypes mapped onto actin (two views 90° apart). Helices are coloured by cluster: Cluster 0 (blue), Cluster 1 (green), Cluster 2 (purple), Cluster 3 (orange). Outliers are denoted Cluster −1 (grey). The four clusters occupy distinct grooves/orientations around the barbed-end surface. **e**, Representative models from each cluster with example protein names annotated (matching the colours in **d**). These exemplars illustrate the sequence and placement diversity captured by the orientation-based clustering.

To assess the structural convergence of each predicted peptide–actin model with the reference ITPKA^Arg28–Ala49^ motif, each candidate underwent a detailed comparison against the ITPKA^Arg28–Ala49^–actin complex. We measured the root mean square deviation (RMSD) for α-carbon atoms (C-α; positions P1–P9), helix orientation (Δθ, Δφ) using a polar coordinate system and helix polarity (Fig. 1g–i). A helix polarity of +1 indicates an orientation in the same amino-to-carboxy terminus direction as the reference ITPKA^Arg28–Ala49^ helix and a polarity value of −1 denotes an inverted orientation. The resulting models were then clustered using hierarchical density-based spatial clustering of applications with noise (hdbscan)^46^, which represents a density-based algorithm that effectively segregates core clusters in the (Δφ, Δθ, polarity)-space and distinguishes them from outliers (Fig. 1i). Cluster 3 displayed minimal RMSD and orientation shifts relative to ITPKA, suggesting a highly similar helix arrangement for actin binding (Fig. 1i and Extended Data Fig. 3). A sequence logo of these 59 clustered hits confirmed strong positional conservation at P1, P4, P8 and P9, with the highest degree of conservation at P1 and P8 (Fig. 1j). Given that this actin-binding motif functions as a SLiM, we designated it as the short linear actin filament-binding motif (SFM).

### Validation of SFM peptides in cells and in vitro

**Fig. 2:**
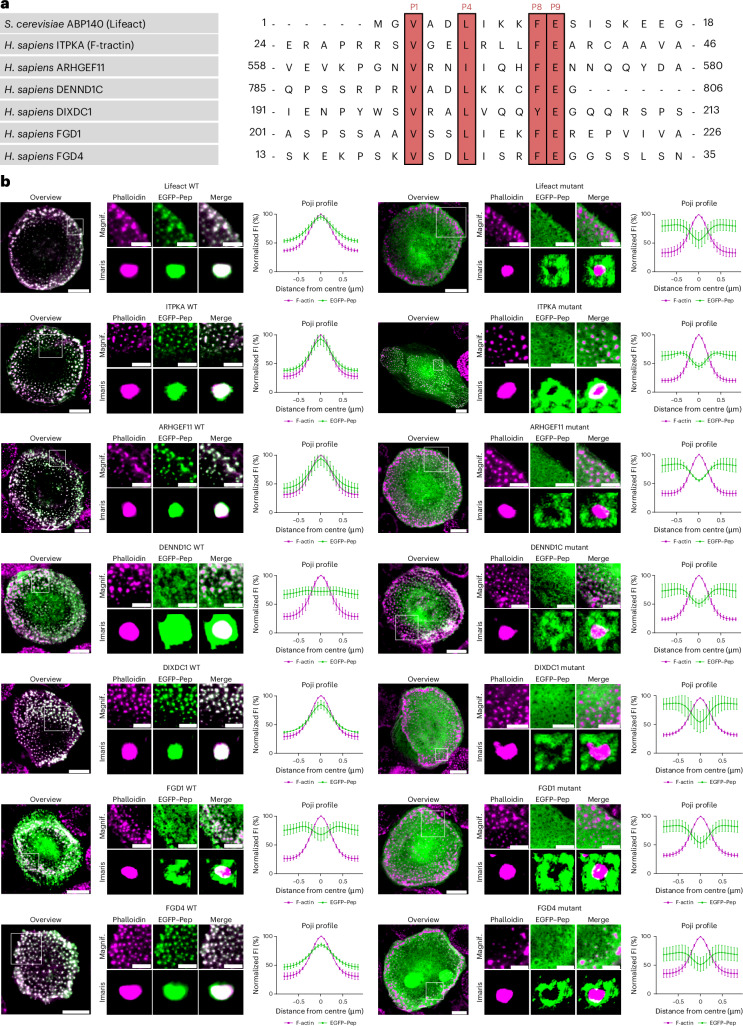
Validation and functional analysis of the short linear actin filament-binding motif by cellular actin filament co-localization. **a**, Five peptides identified by the SLiMFold pipeline were aligned with the ITPKA and Lifeact sequences. The conserved positions P1, P4, P8 and P9 are highlighted in red. **b**, Primary human macrophages were transfected with vectors coding for the EGFP-tagged peptides (EGFP–Pep) depicted in **a** and stained with phalloidin-568 to visualize actin filaments. Representative cell images and magnified views of the boxed region (magnif.) showing a podosome substructure are provided. Scale bars, 10 (main images) and 5 µm (magnified views). Imaris three-dimensional reconstructions and Poji radial profiles of the fluorescence intensities, at a *z* plane of highest fluorescent phalloidin intensity, with the mean ± s.d. of the actin filaments and the respective peptide from 180–953 podosomes from 3 cells are shown (*n* = 3). The Poji radial profiles were normalized to set the intensity values from 0 to 100%. FI, fluorescence intensity. Source data

**Fig. 3:**
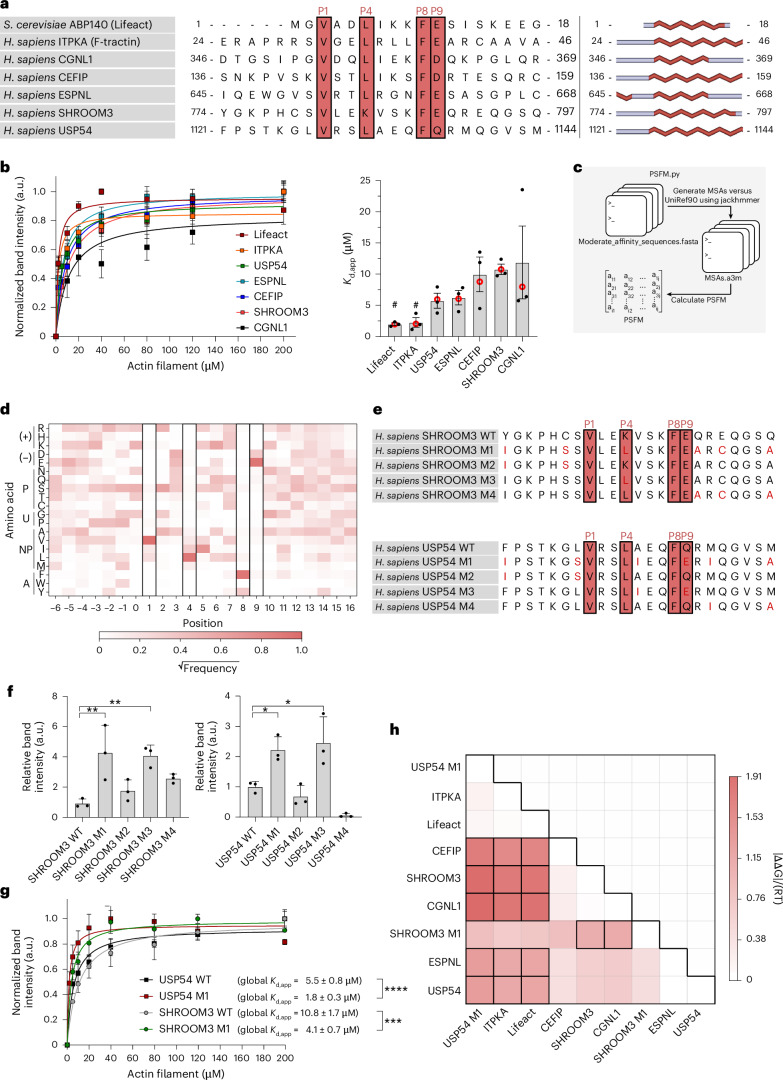
Determination of SFM binding affinities to actin filaments and identification of affinity-modulating amino acids. **a**, Five additional peptides identified by the SLiMFold pipeline were aligned with the sequences of ITPKA and Lifeact (left). The conserved positions P1, P4, P8 and P9 are highlighted in red. Secondary structures of these peptides, from SLiMFold predictions, are shown (right). **b**, The peptides were co-sedimented with actin filaments, analysed by western blotting and the actin filament concentrations were plotted against the normalized band intensities (left). The *K*_d__,app_values for all peptides tested are provided (right). The dots represent the *K*_d__,app_ values obtained from three independent experiments, the bars show the mean ± s.e.m. and red circles indicate the global-fit *K*_d__,app_ values. The ITPKA and Lifeact binding curves were compared using the extra-sum-of-squares *F*-test. ITPKA differed significantly from CGNL1 (*P* = 0.0009), CEFIP (*P* = 0.0009), ESPNL (*P* = 0.0077), SHROOM3 (*P* < 0.0001) and USP54 (*P* = 0.0054) but not Lifeact (*P* = 0.9006). Similarly, Lifeact differed significantly from CGNL1 (*P* < 0.0001), CEFIP (*P* < 0.0001), ESPNL (*P* = 0.0007), SHROOM3 (*P* < 0.0001) and USP54 (*P* = 0.0004) but not ITPKA (*P* = 0.9006). **c**, To identify the amino acids modulating the affinity of the peptides to actin filaments, their occurrence frequency was analysed by generating a PSFM using MSAs from CEFIP, USP54, ITPKA, PPP1R9A, Lifeact, ARHGEF11, FGD4 and DIXDC1. **d**, Position-specific frequency matrix. Positively charged (+), negatively charged (−), polar (P), unique (U), non-polar (NP) and aromatic (A) residues are represented on the *y* axis and the position inside the SFM is shown on the *x* axis. Residues that occur less frequently are shown in white and the more frequent residues in red. **e**, SHROOM3 and USP54 mutants generated based on the PSSM. Mutated positions are labelled in red. **f**, Relative band intensities from mutant SHROOM3–actin (left) and USP54–actin (right) filament co-sedimentation assays normalized to the WT. Data are the mean ± s.e.m. of *n* = 3 biologically independent experiments per SFM. SHROOM3: WT versus M1, *P* = 0.0047; WT versus M2, *P* = 0.6548; WT versus M3, *P* = 0.0070; WT versus M4, *P* = 0.1646. USP54: WT versus M1, *P* = 0.0300; WT versus M2, *P* = 0.8266; WT versus M3, *P* = 0.0112; WT versus M4, *P* = 0.0988. Statistical significance relative to the WT was assessed using a one-way analysis of variance with Dunnett’s multiple comparisons test. **g**, Affinity of USP54 M1 and SHROOM3 M1 for actin filaments. Curves represent global nonlinear regression fits from *n* = 3 independent experiments per SFM. The global best-fit *K*_d__,app_ estimate ± s.e.m. for each dataset is provided. Statistical significance between WT and M1 constructs was determined using extra-sum-of-squares *F*-tests comparing models with shared versus separate *K*_d,app_ values. USP54 WT versus USP54 M1, *P* < 0.0001; SHROOM3 WT versus SHROOM3 M1, *P* = 0.0002. **h**, Pairwise comparison of the binding free-energy differences between all tested SFM peptides. For each peptide pair, the absolute difference in binding free energy (ΔΔG), derived from their *K*_d,app_ values, was normalized to RT (0.589 kcal mol^−1^). This dimensionless metric represents how many thermal energy units separate the affinities of two peptides. Values of <1 reflect differences within the range of thermal noise (|ΔΔG | < RT), whereas values of >1 indicate energetic separation. *P < 0.05, **P < 0.01; ***P < 0.001, ****P < 0.0001; a.u., arbitrary units. Source data

**Extended Data Fig. 4:**
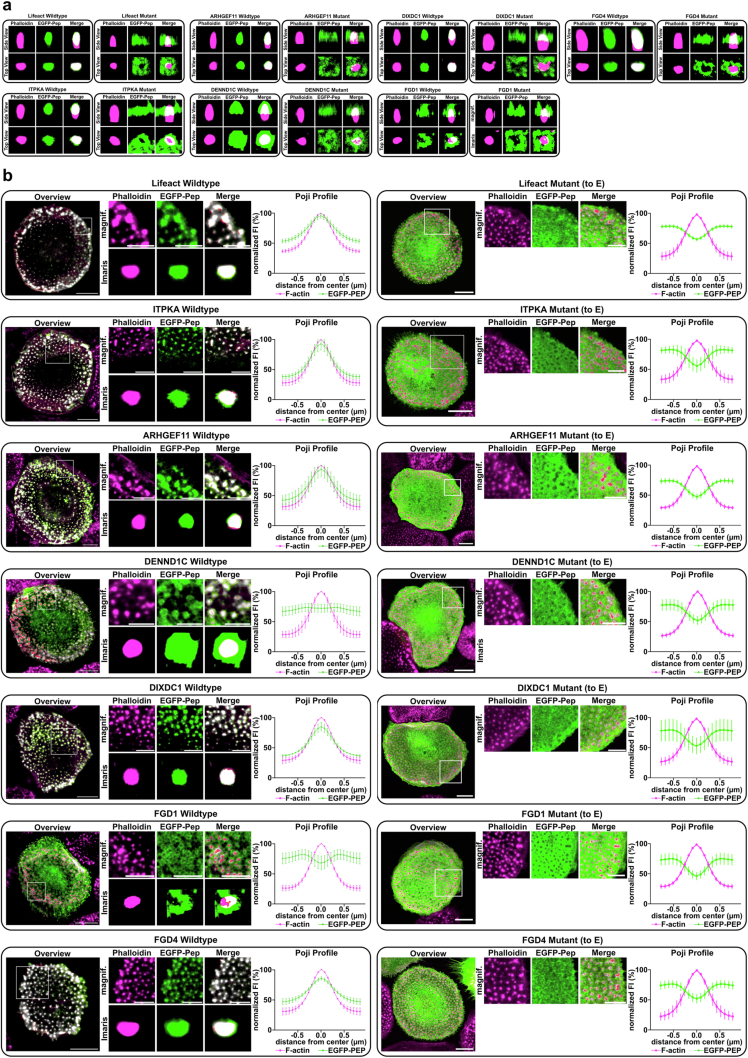
Validation and functional analysis of the novel short linear actin filament-binding motif by cellular actin filament co-localization. **a**, Representative confocal images are shown in both top and side views (Imaris 3D reconstructions), emphasising subcellular peptide localization. Wild-type peptides generally display robust co-localization with F-actin (appearing white in merged panels), whereas alanine substitutions at key residues (P1 or P8) diminish or abolish F-actin association. **b**, Primary human macrophages were transfected with the vectors coding for the EGFP-tagged peptides and their respective mutants (position P1 and P8 were mutated to Glutamate E) and stained with phalloidin-568 to visualize actin filaments (magenta). Representative cell images are shown including zoomed- into one podosome substructure, scale bars: 10 and 5 µm, respectively. Co-localization is evident when EGFP-tagged peptides (green) overlap with phalloidin-stained actin (magenta). Right panels: Imaris 3D reconstructions and Poji radial profiles confirm varying degrees of peptide–actin co-localization. Shown are Poji radial profiles of fluorescence intensities, at a *z* plane of highest fluorescent phalloidin intensity, with the mean values of actin filaments and respective peptide ± s.d. of at least three cells, and 240 podosomes per transfected peptide. Poji radial profiles were normalized to set the intensity values from 0% to 100%. Source data

**Extended Data Fig. 5:**
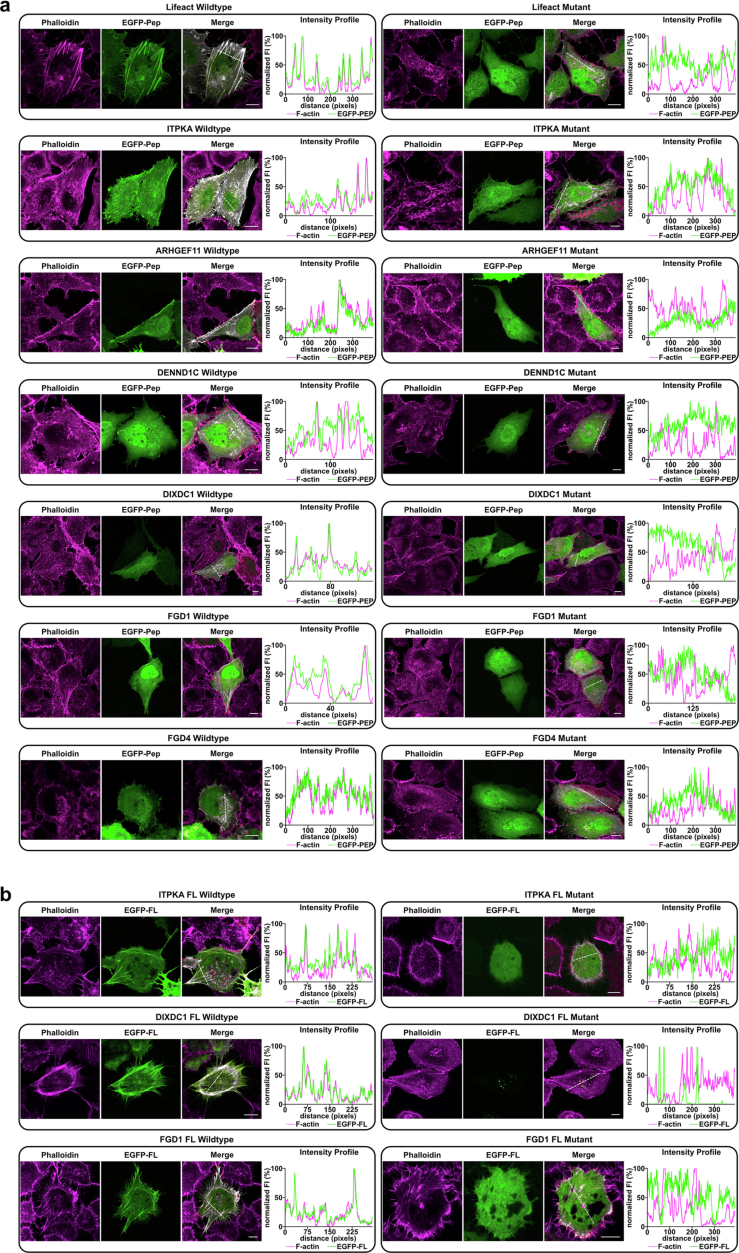
Co-localization of phalloidin-stained actin and EGFP-tagged peptides and proteins in H1299 cells with accompanying intensity profiles. **a**, Representative confocal micrographs show EGFP-tagged peptides (green) alongside phalloidin-568 staining of F-actin (magenta). Merged images highlight varying degrees of co-localization (appearing white). Intensity profiles (right panels) were generated along the dashed white line in each cell, quantifying the overlap between F-actin and peptide signals. Wild-type peptides generally display robust co-localization, whereas alanine substitutions at key positions (P1 or P8) diminish or abrogate actin association. Scale bars, 10 µm. **b**, Representative confocal images show H1299 cells transfected with WT or mutant full-length (FL) constructs fused to EGFP (green), together with phalloidin-568 staining of F-actin (magenta). Merged panels highlight varying extents of co-localization (appearing white). Intensity profiles (right panels) were recorded along the dashed white lines in each cell, quantifying relative fluorescence for F-actin and the respective EGFP-FL protein. Wild-type constructs generally exhibit robust co-localization with actin filaments, whereas alanine substitutions at key motif positions (P1 or P8) disrupt or reduce F-actin association. Scale bars, 10 µm. All imaging experiments in **a** and **b** were independently repeated three times with similar results; representative cells are shown. Source data

**Extended Data Fig. 6:**
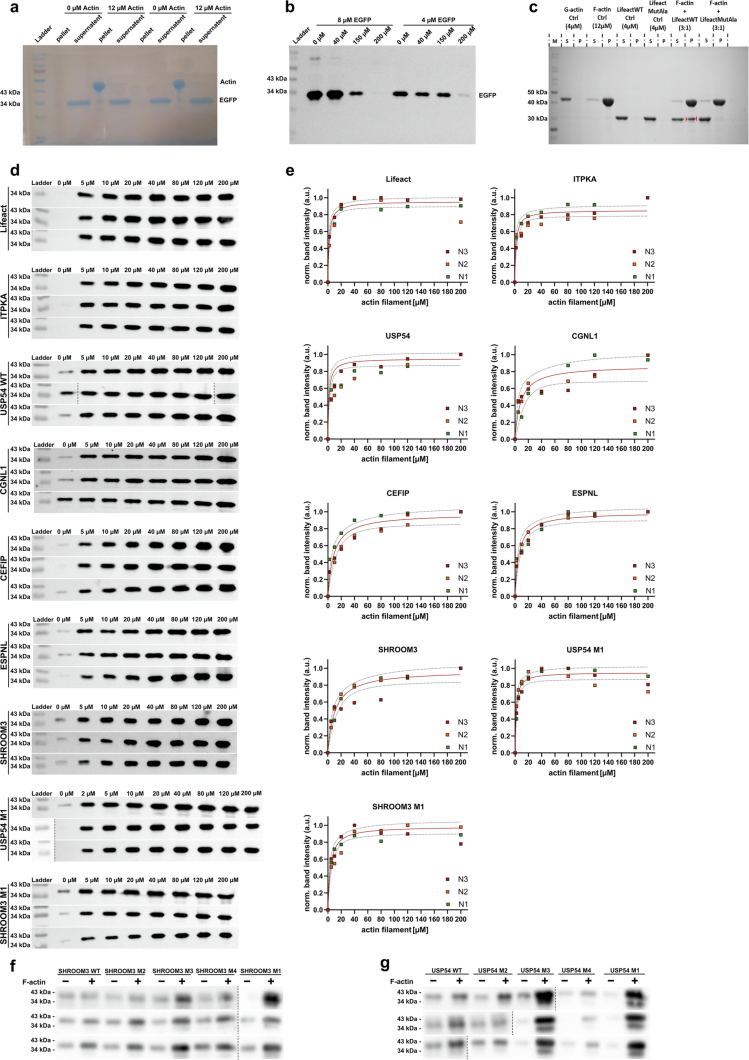
In vitro filamentous actin co-sedimentation assay of EGFP-tagged peptides and mutant analyses. **a**, Control co-sedimentation assay with EGFP. EGFP was centrifuged at 100,000*g* in the absence or presence of 12 µM actin. Supernatant and pellet fractions were analysed by Coomassie-stained SDS–PAGE. Shown is a representative control experiment performed once. **b**, Control co-sedimentation assay with EGFP only. EGFP (8 µM or 4 µM) was centrifuged at 100,000*g* in the absence or presence of increasing actin concentrations (0–200 µM). Pellet fractions were analysed by western blotting using anti-EGFP. Shown is a representative control experiment performed once. **c**, Control co-sedimentation assay with Lifeact WT and Lifeact P1/P8 mutants. Globular actin and filamentous actin (4 µM or 12 µM) were centrifuged at 100,000*g* in the absence or presence of EGFP–Lifeact WT or EGFP–Lifeact MutAla. Supernatant and pellet fractions were analysed by SDS–PAGE. The ‘MutAla’ mutant contains V3A and F10A substitutions, targeting the P1/P8 positions within the SFM. Shown is a representative control experiment performed once. **d**,**e**, Western blots and graphs of actin pulldown assays for each peptide. The pellet fraction is shown, with peptide bands visualized using anti-EGFP. These assays were performed to determine the binding affinities (*K*_d__,app_) of wild-type (WT) and mutant peptides. Experiments were independently repeated three times. **f**, Quantified relative band intensities from SHROOM3 pulldown assays, normalized and averaged across three independent experiments, showing differences in binding affinities between WT and mutants. **g**, Quantified relative band intensities from USP54 pulldown assays, normalized and averaged across three independent experiments, highlighting differences in binding affinities between WT and mutant peptides. Source data

To validate binding of the predicted SFM peptides to actin filaments, we randomly selected candidates and split them into two groups. Group 1 included PH domain-containing proteins 1 and 4 (FGD1 and -4), DENN domain-containing protein 1 (DENNDC1), Rho guanine nucleotide exchange factor 11 (ARHGEF11) and DIX domain-containing protein 1 (DIXDC1; Fig. 2a and Supplementary Tables 3,4). Group 2 comprised the actin bundling proteins Shroom family member 3 (SHROOM3), Espin-like (ESPNL), ubiquitin specific peptidase 54 (USP54), Cardiac-enriched FHL2-interacting protein (CEFIP) and the tight junction protein Cingulin-like 1 (CGNL1; Fig. 3a and Supplementary Tables 3,4). The Group 1 candidates were validated by actin filament co-localization with enhanced green fluorescent protein (EGFP)-tagged peptides or full-length proteins in primary human macrophages and H1299 cells (Fig. 2b and Extended Data Figs. 4,5). The Group 2 candidates were validated by actin filament co-sedimentation assays with recombinant peptides (Fig. 3b and Extended Data Fig. 6).

The Group 1 SFM peptides, including the positive controls ITPKA^Arg28–Ala49^ and Lifeact, were transiently expressed as EGFP fusion peptides in primary human macrophages, allowing quantification of co-localization with actin filament subsets^47^. We additionally tested site-specific mutants (Val to Ala and Phe to Ala; Val to Glu and Phe to Glu) at positions 1 and 8. As shown in Fig. 2 and Extended Data Fig. 4, Lifeact, ITPKA^Arg28–Ala49^, FGD4 and ARHGEF11 co-localized prominently with podosome cores. However, DENND1C predominantly localized to the podosome cap, whereas FGD1 displayed a broader distribution, partially overlapping the podosome core (Extended Data Fig. 4). On the other hand, all P1 and P8 mutants were diffusely distributed, confirming that positions P1 and/or P8 are essential for actin filament binding (Fig. 2 and Extended Data Fig. 4(right)). Similar results were obtained in H1299 cells (Extended Data Fig. 5). Together, these data indicate that the SFM binds to actin filaments with different preferences to actin filament subpopulations.

To further confirm these findings, we tested full-length versions of selected proteins in H1299 cells and introduced the mutations at P1/P8. Co-localization with actin filaments was observed for the wild-type (WT) full-length proteins but completely disappeared following mutation of the residues at P1/P8 (Extended Data Fig. 5). This result highlights the critical role of the SFM in mediating actin filament binding not only in isolated peptides but also in full-length proteins.

For the Group 2 candidates, the peptides’ affinities for actin filaments were assessed using a co-sedimentation assay. Here actin filaments and associated EGFP-tagged peptides are enriched in the pellet fraction. Western blotting was used to quantify the EGFP-tagged peptides in the pellet following titration with actin at concentrations ranging from 5 to 200 µM. Analysis of band intensities allowed determination of the apparent dissociation constants (*K*_d,app_) for binding to actin filaments (Fig. 3b and Extended Data Fig. 6). The positive controls, Lifeact and ITPKA, bound to actin filaments with a mean *K*_d__,app_ of approximately 2 µM. In contrast, the identified peptides exhibited weaker binding affinities, with a mean *K*_d__,app_ ranging from 6 to 12 µM. Among these, the binding behaviour for ITPKA and Lifeact compared with USP54, ESPNL, CEFIP, SHROOM3 and CGNL differed significantly. To identify potentially physically relevant *K*_d__,app_ differences above thermal noise, we determined the corresponding change in Gibbs free energy (ΔG) values (*R* = 0.0019872 kcal (mol·K)^−1^, *T* = 296.15 K) and calculated the ΔΔG between all pairs of biochemically validated SFM peptides. To visualize these *K*_d__,app_ differences, a colour-coded ΔΔG matrix highlighting values that were >0.589 kcal mol^−1^ (thermal noise RT ≈ 0.589 kcal mol^−1^ at 296 K) was constructed (Fig. 3h). This analysis revealed moderate but distinct differences between the ITPKA and Lifeact, and the USP54, ESPNL, CEFIP, SHROOM3 and CGNL group (Fig. 3h). In summary, our data indicate that SFM peptides can bind to actin filaments with moderately varying affinities.

### Amino acids modulating affinity to actin filaments

The data depicted in Fig. 3b revealed that the binding affinities of all tested SFM peptides showed moderate differences, although they contained the conserved residues at P1 and P8. This raises the question whether amino acids flanking the motif modulate the affinity.

To address this, we aimed to identify the amino acid in the SFM modulating the affinity to actin filaments. Jackhmmer-generated MSAs of the peptide sequences from ITPKA, CEFIP, USP54, FGD4, ARHGEF11, DIXDC1 and Lifeact were used to generate a position-specific frequency matrix (PSFM; Fig. 3c). Homologous sequence alignment revealed amino acid frequency, ranging from highly frequent (in dark red) to infrequent (white; Fig. 3d). Given that the highly frequent amino acids interact with actin, the residues shown in white or light red might represent candidate mutation sites for replacement with more frequently occurring amino acids.

On this basis, three hotspots were defined: an N-terminal, an internal (between P1 and P9) and a C-terminal motif. Targeted mutations in SHROOM3 and USP54 were introduced to test whether substitution of a low-frequency amino acid with a high-frequency amino acid would enhance binding of the SFM peptides to actin filaments. The point substitutions Tyr1Ile and Cys6Ser (M2), Lys11Leu (M3), and Gln17Ala, Glu19Cys and Gln23Ala (M4) were produced in SHROOM3 along with a triple-hotspot variant (M1). Similarly, Phe1Ile and Leu7Ser (M2), Ala12Ile and Gln16Glu (M3), and Met18Ile and Met23Ala (M4) USP54 mutants were designed along with USP54 M1, which included all mutations (Fig. 3e).

Compared to the WT protein, mutation of the residues between conserved P1 and P9 significantly enhanced binding to actin filaments—fourfold in SHROOM3 M3 and threefold in USP54 M3 (Fig. 3f). However, mutations N- or C-terminal to P1 and P9 (SHROOM3 M2 and M4, and USP54 M2 and M4) had no significant effects (Fig. 3f). Moreover, the *K*_d__,app_ of USP54 M1 (1.7 ± 0.2 µM) and SHROOM3 M1 (3.9 ± 0.39 µM) were significantly lower (*P* < 0.005) than their WT counterparts (USP54, 6 ± 0.9 µM; SHROOM3, 10.8 ± 0.9 µM); the corresponding ΔΔG (0.74 and 0.61 kcal mol^−1^, respectively) also indicated moderate differences (Fig. 3f,g,h). These data suggest that the amino acids between conserved P1 and P9 modulate the binding affinity of the SFM peptides.

### Cryogenic electron microscopy structure of USP54 M1 highlights critical actin-binding residues

The affinities of USP54 and SHROOM3 M1 for actin filaments were higher than their WT counterparts (Fig. 3e–h). Specifically, the SHROOM3 M3 Lys11Leu (P4) mutation enhanced actin binding relative to WT SHROOM3, whereas in USP54, the Ala12Ile (P5) and Gln16Glu (P9) substitutions resulted in increased actin filament affinity.

The enhanced binding of SHROOM3 M1 can be attributed to the Lys-to-Leu substitution, as the Leu at P4 is conserved within the SFM. In contrast, the USP54 M1 mutant contains two relevant substitutions, Ala12Ile (P5) and Gln16Glu (P9). Among these, only P9 corresponds to a conserved actin-binding site (Fig. 3a), whereas P5 has not been predicted to substantially contribute to actin interaction. Moreover, our PSFM prediction (Fig. 3c) indicates that the glutamine at P9 in WT USP54 represents a low-frequency (white) amino acid. This suggests that the Gln-to-Glu substitution probably accounts for the increased actin-binding strength of USP54 M3 more than the Ala-to-Ile substitution.

**Fig. 4:**
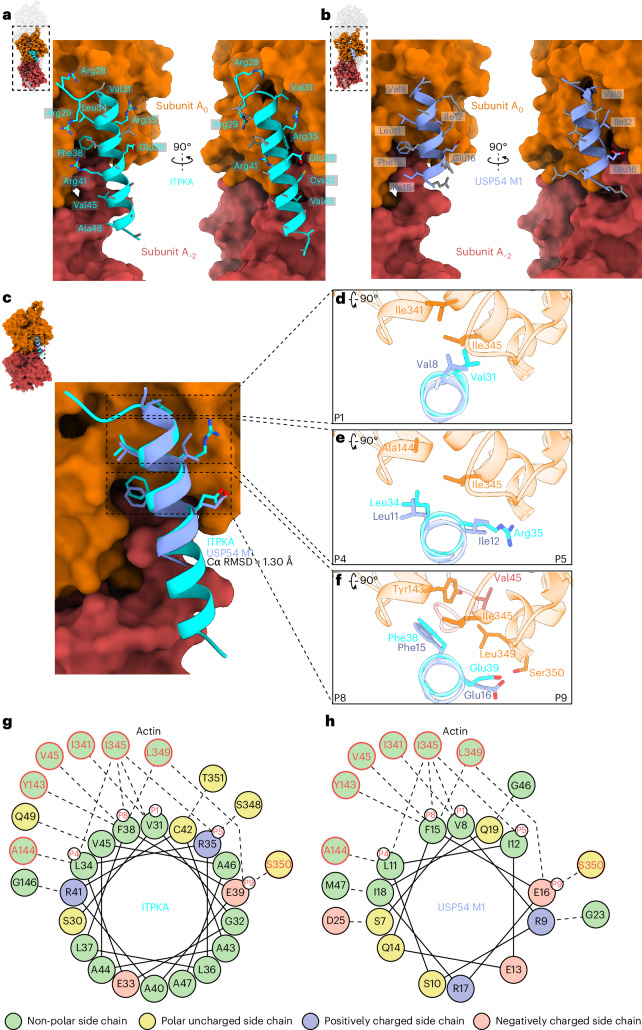
Structural comparison of ITPKA–SFM and USP54 M1–actin complexes. **a**, ITPKA–SFM (cyan) bound to actin filaments shown as molecular surface (central subunit A_0_ in orange, neighbouring subunit A_−2_ in red). Two views are shown, rotated by 90°. **b**, Same representation for the USP54 M1 helix (blue) shown in two orientations related by a 90° rotation. **c**, Superposition of the ITPKA and USP54 M1 helices on the same actin pocket. Cα RMSD = 1.30 Å (least-squares fit on the helices residues), indicating a highly similar placement and orientation. **d**–**f**, Magnified views of the conserved anchor positions P1 (**d**), P4 and P5 (**e**), and P8 and P9 (**f**) in a 90° top view. **g**,**h**, Helical-wheel projections for ITPKA (**g**) and USP54 M1 (**h**). Circles indicate helix residues coloured by chemistry. Dotted lines denote contacts to actin; actin residues contacted by both peptides are highlighted in red.

**Extended Data Fig. 7:**
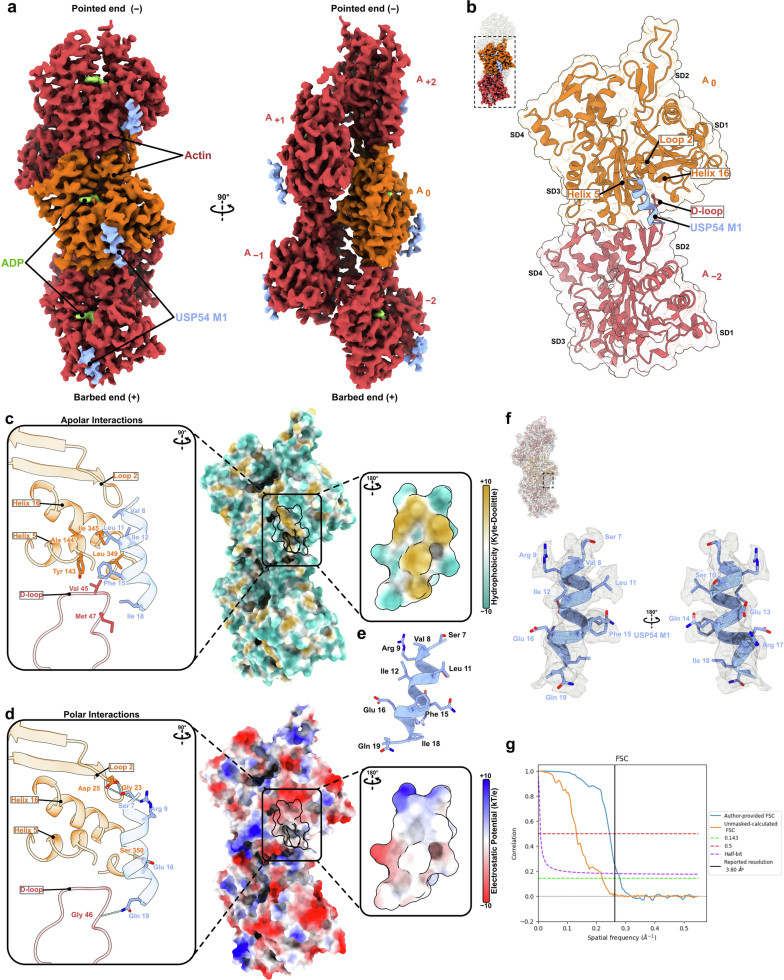
Cryo-EM structure of the USP54 M1–actin complex. The sample contained an USP54 M1–GFP fusion; due to flexibility, no density is observed for GFP or the linker, and only the USP54 M1 helix (residues 7–19) is resolved. **a**, The electron density map illustrates the USP54 M1–actin complex. A single USP54 M1 helix of residue 7–19 (blue) is resolved, forming the primary binding interface with actin. Actin subunits are displayed with the central subunit in orange and surrounding subunits in red. ADP is shown in light yellow. **b**, A cartoon representation of the USP54 M1–actin complex shows USP54 M1 bridging two adjacent actin subunits. Key interaction regions in actin helix 5 (AA 137–145), helix 16 (AA 338–348), loop 2 (AA 21–28), the D-loop (AA 38 -51) and actin subdomains SD1–SD4 are labelled. **c**, Apolar interactions between USP54 M1 and actin are depicted by surface colour-coding for hydrophobicity (yellow = hydrophobic). Left panel: zoomed-in view and a 90° rotation highlight interacting residues displayed as sticks. Right panel: zoomed-in view and a 180° rotation; here hydrophobicity scores are shown (low: cyan, high: yellow). **d**, Polar contacts between USP54 M1 and actin are shown via electrostatic potential mapping on actin (negative: red, positive: blue). Left panel: zoomed-in view and a 90° rotation highlight interacting residues displayed as sticks. Right panel: zoomed-in view and a 180° rotation, here electrostatic scores are shown (negative: red, positive: blue). **e**, The binding helix of USP54 M1 is separately depicted as a cartoon with side chains shown as sticks. **f**, Left, the full actin filament model is shown with a semi-transparent molecular surface so that the underlying cartoon representation remains visible; boxes mark the bound SFM. Right, zoom-in views show the USP54 M1 SFM helix with its corresponding cryo-EM density (grey mesh) in two orientations related by a 180° rotation around the helix axis. USP54 M1 SFM (blue) follows well-defined α-helical density with resolved side chains (labelled), enabling unambiguous sequence registry (Ser7–Gln19). **g**, Map–model Fourier shell correlation (FSC) curves. Shown are FSCs between the refined model and the unsharpened half-maps, with standard 0.5 and 0.143 criteria indicated.

To obtain structural insights into the binding modus of the USP54 M1–actin filament complex, we solved the cryo-EM structure of the USP54 M1–actin complex (Fig. 4 and Extended Data Fig. 7). The mutant helix binds to the canonical SFM pocket in the same orientation and polarity as ITPKA and Lifeact. The conserved P1, P4, P8 and P9 adopt similar conformations and interact with the same actin amino acids (ITPKA and USP54 M1 have an alpha carbon (Cα) RMSD of 1.30 Å; Fig. 4a–f).

Helical-wheel projections (Fig. 4g,h) illustrate the amino acids within the ITPKA–SFM and USP54 M1–SFM peptides that interact with actin. As expected, the conserved residues at P1, P4 and P8 display similar interaction patterns with actin in ITPKA and USP54 M1. Furthermore, the introduced glutamate at P9 in USP54 M1 interacts with actin residues Ser350 and Leu349. In contrast, the Ile residue at P5 in USP54 M1 only interacts with one amino acid inside the actin molecule, that is, Ile345. These findings suggest that the Gln-to-Glu mutation is primarily responsible for the enhanced actin-binding strength of USP54 M1.

Collectively, our structural and biochemical analyses indicate that the conserved positions P4 and P9 play key roles in modulating the affinity of SFM peptides for actin filaments.

### Molecular dynamics simulations reveal that SFM peptide binding reduces actin filament rigidity

Although our static structural and biochemical data define how conserved SFM positions bind to filamentous actin, recent work highlighted a bidirectional link between cellular mechanical load, filament mechanics and ABP engagement^48,49^. In particular, cofilin-decorated actin represents a well-characterized mechanically altered filament state with substantially reduced persistence length (*L*_p_) compared with undecorated actin filaments, resulting in higher bending flexibility^50^. To determine whether SFM-containing peptides might induce a similar mechanical effect, we performed all-atom molecular dynamics (MD) simulations of filamentous actin alone and in complex with either the ITPKA or USP54 M1 peptide. Simulations were run for 40 ns using 13-mer filaments reconstructed from our cryo-EM structures in the ADP–Mg^2+^ state in the presence of 180 mM KCl.

**Fig. 5:**
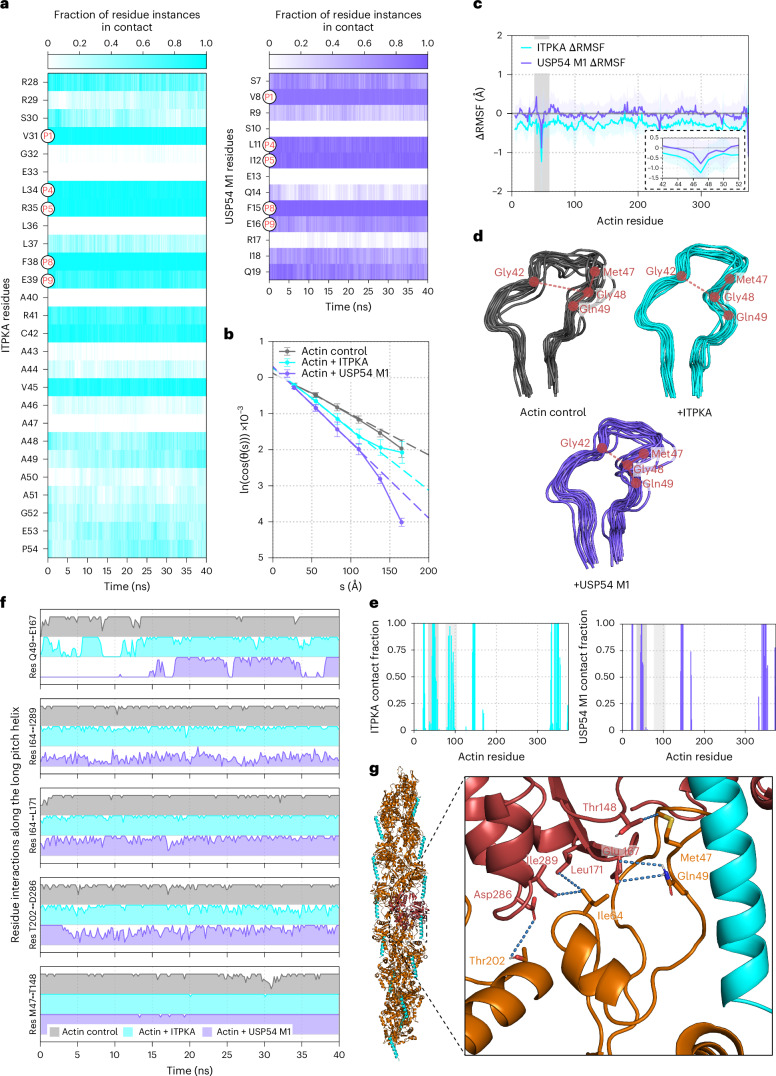
Molecular dynamics simulations reveal SFM-induced stabilization of the actin D-loop and increased filament bending flexibility. **a**, Time-resolved contact occupancy for SFM peptides binding to actin filament. For each SFM residue (ITPKA, left; USP54 M1, right), the fraction of residue instances in contact with actin is plotted over the 40 ns trajectory. Colour intensity indicates contact fraction (white, no contact; cyan/purple, sustained contact). **b**, Actin filament stiffness is decreased by SFM binding. Decay of the tangent–tangent correlation function plotted against contour separation (*s*) for actin alone, actin + ITPKA and actin + USP54 M1. Data points represent the mean ± s.e.m. from spatial (11 subunits) and temporal (36 ns) averaging. Dashed lines show linear fits to the initial decay region, from which the *L*_p_ is extracted as *L*_p_ = −1 / slope. **c**, Differential RMSF (ΔRMSF; heavy atoms), computed as (actin + SFM) − (actin alone) for both ITPKA and USP54 M1, plotted across all actin residues. The shaded bands indicate the s.d. across the 11 actin subunits. Inset: expanded view of the D-loop region (residues 42–52) to highlight the residues most affected by SFM binding. **d**, The D-loop conformational ensemble demonstrates SFM-induced rigidification. Overlaid trajectory snapshots of the D-loop region (residues 35–55) from actin alone (top left), actin + ITPKA (top right) and actin + USP54 M1 (bottom). Key D-loop residues (Gly42, Met47, Gly48 and Gln49) are indicated. **e**, SFM contact occupancy mapped onto the actin structure. Bar plots show the fraction of trajectory frames in which each actin residue contacts an SFM peptide for ITPKA (left) and USP54 M1 (right). The D-loop regions are highlighted in grey and contact hotspots in light grey. Contact fractions approaching 1.0 indicate stable, persistent binding throughout the 40 ns simulation. **f**, Time-resolved occurrence of specific inter-residue contacts. Contact density plots for five key residue pairs between long-pitch neighbouring actin protomers (subunits i and i-2) tracked over 40 ns: Q49(i)-E167(i-2), I64(i)-L171(i-2), I64(i)-I289(i-2), T202(i)-D286(i-2), M47(i)-T148(i-2). Residue-pair labels are written as subunit i (lower protomer) − subunit i-2 (upper protomer). Each horizontal band represents one system (actin alone and the two SFM-bound conditions); bar height indicates the fraction of subunits where the contact is present. Per cent changes reported in the main text correspond to the change in contact occupancy relative to actin alone (actin + SFM minus actin), expressed in percentage points. The residue pairs shown correspond to the most significant SFM-dependent interaction changes. **g**, Structural context of monitored inter-residue contacts in **f**. Actin subunits are coloured in red and orange, and the ITPKA helix in cyan. All analyses are based on *n* = 1 independent MD simulation per condition. Source data

The core motif residues of both SFM peptides remained stably bound throughout the 40-ns trajectories (Fig. 5a) and, in agreement with previously published *L*_p_ values for undecorated filaments^50,51^, the control simulation yielded an *L*_p_ of 9.4 ± 1.2 µm. This value was obtained after discarding the initial 4 ns and block-averaging the remaining 36 ns; fitting the full 40-ns trajectory resulted in an *L*_p_ of 8.8 µm (Fig. 5b)^50,51^. Strikingly, a pronounced *L*_p_ decrease was observed when filaments were decorated with ITPKA or USP54 M1 peptides (Fig. 5b). The block-averaged *L*_p_ of ITPKA-decorated filament was 6.3 ± 0.7 µm (*L*_p_ of 5.9 µm for the fit across the full 40 ns) and that of USP54 M1 was 5.1 ± 0.8 µm (*L*_p_ of 4.8 µm for the fit across the full 40 ns).

**Extended Data Fig. 8:**
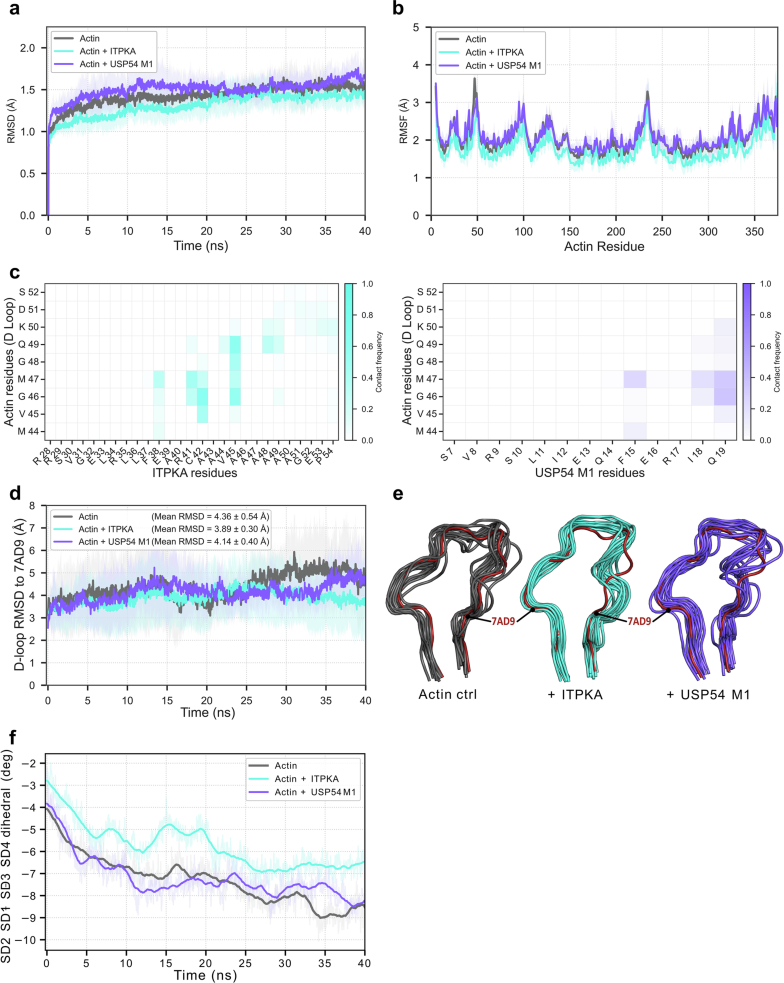
Molecular dynamics simulations. **a**, Time-resolved backbone RMSD trajectories demonstrate system stability. Global RMSD (Cα atoms) computed for actin alone (grey), actin + ITPKA (cyan) and actin + USP54 M1 (purple) over 40 ns of simulation. Lines indicate the mean RMSD and shaded bands indicate SD across the 11 actin subunits within the filament. The trajectories indicate that the overall filament architecture remains intact without structural disintegration, with mean RMSD values of ~1.3–1.6 Å indicating well-behaved simulations suitable for mechanical analysis. **b**, Per-residue RMSF profiles reveal region-specific effects of SFM binding on actin dynamics. Root mean square fluctuation (all heavy atoms) plotted across all actin residues (1–375), showing the mean ± s.d. for each system. All three systems exhibit characteristic high-flexibility regions, including prominent peaks at the D-loop (~40–50). Notably, SFM binding does suppress D-loop fluctuations, with both ITPKA and USP54 M1 showing reduced RMSF amplitudes in this region compared with actin alone, indicating local stabilization at the binding interface. **c**, Residue-level contact frequency heat maps identify key SFM–actin interaction hotspots. Left panel: ITPKA residue contacts mapped against actin D-loop residues (44–52), showing sustained high-occupancy contacts (cyan, approaching 100% occupancy) at specific positions throughout the 40 ns trajectory. The contact pattern reveals that actin residues 45–49 are primarily targeted, with ITPKA residue F38 (P8), R41(P11), C42(P12) and V45 (P15) maintaining the most persistent interactions. Right panel: USP54 M1 shows a similar contact pattern (purple), with the same actin residues (45–49) serving as the primary binding interface, contacted by USP54 M1 residues F15 (P8), I18 (P11) and Q19 (P12). **d**, Time-resolved D-loop deviation from the 7AD9 reference. Each actin subunit was aligned to 7AD9 using core Cα atoms (residues 5–375; excluding residues 38–54), and D-loop Cα RMSD (residues 46–50) was calculated per frame and averaged across chains (mean ± s.d.) for actin alone (grey), actin + ITPKA (cyan), and actin + USP54 M1 (purple). **e**, Structural superposition of the Lifeact-bound D-loop (PDB 7AD9) with representative ensembles from MD simulations. The 7AD9 reference structure is shown in red and is overlaid with sampled conformations for actin alone (grey), Actin–ITPKA (cyan), and Actin–USP54 M1 (purple). **f**, Time evolution of the actin monomer flatness defined by the SD2-SD1-SD3-SD4 dihedral angle (calculated as per-frame averages over the nine internal actin monomers, excluding the two terminal subunits at each end to avoid edge effects). The trajectories show the dihedral angle for actin alone (grey) and actin + USP54 M1 (purple) decreasing to approximately −9°, while actin + ITPKA (cyan) maintains a less negative angle (averaging approximately −6.5°) over the course of the simulation. Source data

To assess how SFM binding affects filament dynamics at the atomic level, we computed differences in heavy-atom root mean square fluctuation (RMSF) of SFM-bound and undecorated filaments, and found an unexpected rigidification of the actin D-loop in the presence of both SFM peptides (Fig. 5c and Extended Data Fig. 8). Figure 5d shows representative D-loop ensembles from undecorated and ITPKA- or USP54-decorated actin filaments, and reveals pronounced D-loop flexibility in undecorated actin. USP54 decoration moderately reduces D-loop flexibility, which is further restricted in the presence of ITPKA, resulting in pronounced rigidification as reflected by the strong alignment of representative D-loop conformations. This marked ITPKA-mediated effect could be mediated by the formation of additional contacts with the D-loop and residues 80–100 in SD1 (Fig. 5e).

We next investigated how ITPKA and USP54 M1 affect lateral actin–actin interactions within the filament. Actin residues that directly contact the SFM exhibited the largest immediate local changes in inter-subunit contacts along the long-pitch helix (subunit i to subunit i-2). This observation is consistent with a competitive ‘sequestration’ mechanism in which SFM binding occludes interface atoms that would otherwise mediate inter-protomer actin–actin interactions. For example, both peptides induced a reduction of the overall contact occupancy for the inter-protomer contact of residues Gln49–Glu167 (ITPKA, −22%; USP54 M1, −26%; change in contact occupancy versus actin alone in percentage points; Fig. 5f), in agreement with direct engagement of the D-loop tip by the SFM.

Beyond these local D-loop contact rearrangements, both peptides also induced pronounced changes in actin–actin contacts distal to the SFM binding site, suggesting an allosteric effect. Specifically, we observed strong decreases in inter-subunit contacts such as Ile64–Ile289 (ITPKA, −7%; USP54 M1, −30%), Ile64–Leu171 (ITPKA, −6%; USP54 M1, −17%) and Thr202–Asp286 (ITPKA, −6%; USP54 M1, −17%) together with increases including Met47–Thr148 (ITPKA, +20%; USP54 M1, +16%; Fig. 5f,g). These coordinated gains and losses indicate a redistribution of load-bearing lateral contacts across the filament interface rather than a simple net loss of contacts at the binding patch.

Collectively, these SFM-driven changes suggest that peptide binding remodels lateral actin–actin coupling and lowers effective inter-chain connectivity at key interface edges. We propose that this reweighting of inter-protomer interactions could provide a mechanistic explanation for the observed reduction in *L*_p_ and the resulting increase in filament bending flexibility.

### The amino acids inside the SFM are conserved over evolution

**Fig. 6:**
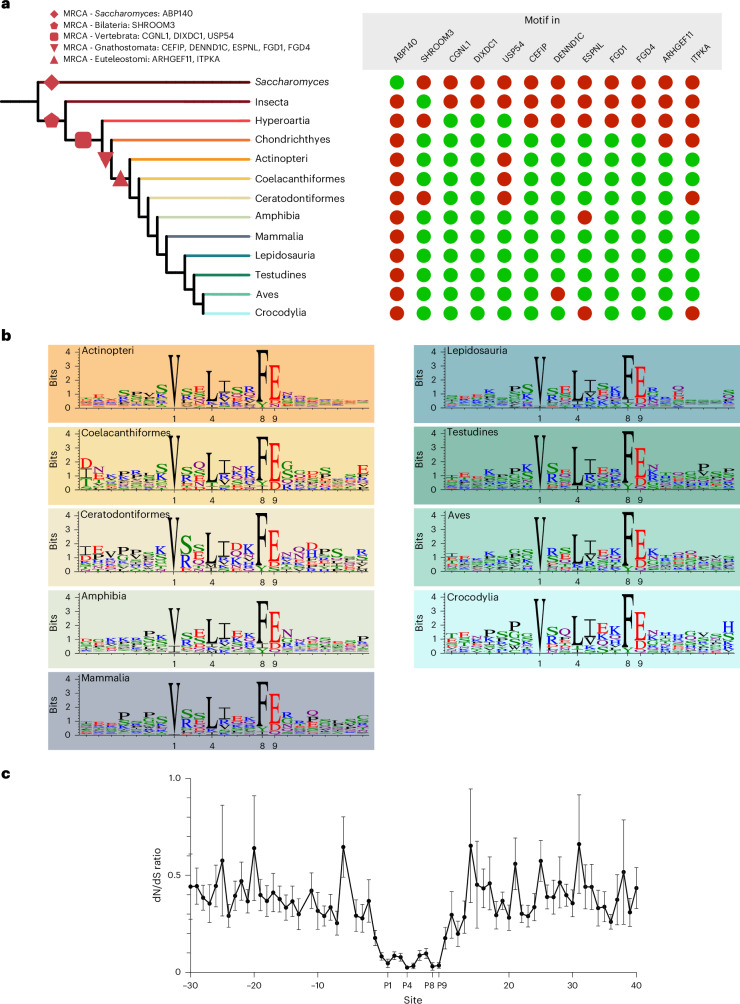
Evolutionary analysis of validated SFM-containing proteins. **a**, Phylogenetic tree illustrating the origin and retention of the SFM. Simplified species tree of the sampled clades (left); the MRCA symbols shown on the tree indicate the inferred first appearance of the SFM within each gene. Clade × gene matrix for validated SFM-containing genes (right); green, motif present; red, motif absent or not detected. **b**, Class-pooled sequence logos for the SFM across nine representative organism classes (Actinopteri, Coelacanthiformes, Ceratodontiformes, Amphibia, Mammalia, Lepidosauria, Testudines, Aves and Crocodylia). For each class, the logo is built from all instances contributed by the 13 validated SFM-containing proteins that possess a confidently detected SFM in that class. Proteins that lack a confidently detected SFM in a given class do not contribute. Key motif positions P1, P4, P8 and P9 are labelled on the *x* axis. The letter height reflects residue conservation—larger letters indicate higher residue conservation. **c**, Analysis of the dN/dS ratio of all validated SFM-containing peptides (*n* = 12). Data are the mean ± s.e.m. The conserved SFM residues (P1, P4, P8 and P9) are annotated. Source data

To obtain further insight into the evolutionary development of the motif, phylogenetic trees tracing each validated SFM-containing protein back to its most recent common ancestor (MRCA) were constructed (Fig. 6a). This analysis revealed the earliest appearance of the SHROOM3 SFM in Bilateria, followed by the SFMs of DIXDC1, USP54 and CGNL1 in Vertebrata. The SFMs of CEFIP, DENND1C, ESPNL, FGD1 and FGD4 emerged in Gnathostomata, whereas the motifs of ARHGEF11 and ITPKA arose in Euteleostomi (Fig. 6a). Importantly, searches across additional metazoan phyla, including Cnidaria, Mollusca and Nematoda, did not identify motif-positive orthologues, indicating that the SFM (with the exception of ABP140 and Lifeact among the proteins analysed) emerged comparatively late in animal evolution. Moreover, an early MRCA does not imply universal retention across descendant lineages. The patchy distribution across higher taxa is instead consistent with lineage-specific loss or divergence, as summarized in the presence–absence matrix in Fig. 6a. The absence of conserved motif-bearing exons or modules, the patchy taxonomic presence within otherwise clear orthologues and a motif age younger than gene age indicate a convergent evolution and a spontaneous origin (ex nihilo) of the SFM.

To examine the evolutionary conservation of the SFM and each position, we generated class-pooled sequence logos of the SFM by aggregating all motif instances from the 13 validated SFM-containing proteins that carry a detected SFM in each organism class (Fig. 6a,b). The classes analysed were: Actinopteri, Coelacanthiformes, Ceratodontiformes, Amphibia, Mammalia, Lepidosauria, Testudines, Aves and Crocodylia. Proteins lacking a class-specific SFM do not contribute to the corresponding sequence logo. The resulting logos consistently highlighted Val (P1), Leu (P4), Phe (P8) and Glu (P9) as predominantly conserved residues across the taxa. Although a small number of substitutions were observed at these positions in certain species, these core residues remained largely unchanged, indicating strong evolutionary constraints (Fig. 6b). This observation aligns with our initial comparison of ITPKA and Lifeact (Fig. 1), where a similar set of core residues were conserved. To accurately quantify the selective evolutionary pressure acting on these conserved positions, we calculated the non-synonymous-to-synonymous mutation ratio (dN/dS)^52^, where values of <1 indicate purifying selection within each SFM-containing gene. We then averaged these values around the SFM core (Val (P1) to Glu (P9)) for all proteins. The resulting low dN/dS ratio relative to nearby flanking regions indicates that the SFM is under purifying (negative) selection, confirming its functional importance (Fig. 6c).

To conclude, these analyses indicate strong purifying selection on the SFM core and a scenario of ex nihilo development across gene lineages.

### Final iteration of the SLiMFold pipeline delivers 103 SFM candidates with different biological roles

**Fig. 7:**
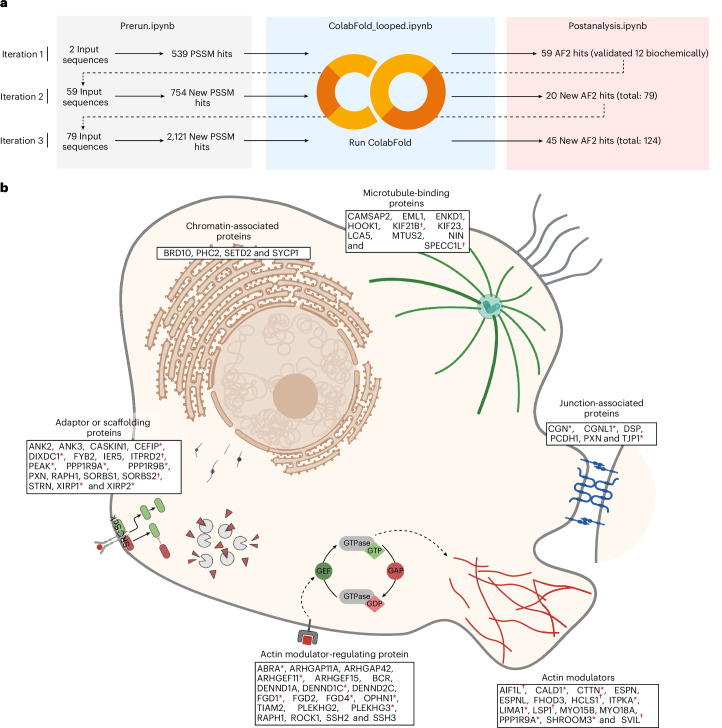
Iterative SLiMFold pipeline expansion and functional classification of newly identified SFMf-containing proteins. **a**, Schematic of three iterative SLiMFold rounds (detailed description in Fig. 3) including in vitro and validation in cells. AF, AlphaFold2. **b**, Eukaryotic cell map illustrating the functional categories of newly identified SFM-bearing proteins. The SFM candidates are grouped according to their principal roles and subcellular localizations. *Proteins for which actin filament binding has been attributed to the SFM (for example, validated by truncation studies); †proteins that bind actin filament but lack direct evidence linking the interaction specifically to the SFM. Panel **b** created in BioRender: Paraschiakos, T. https://biorender.com/35bnaup (2026).

**Extended Data Fig. 9:**
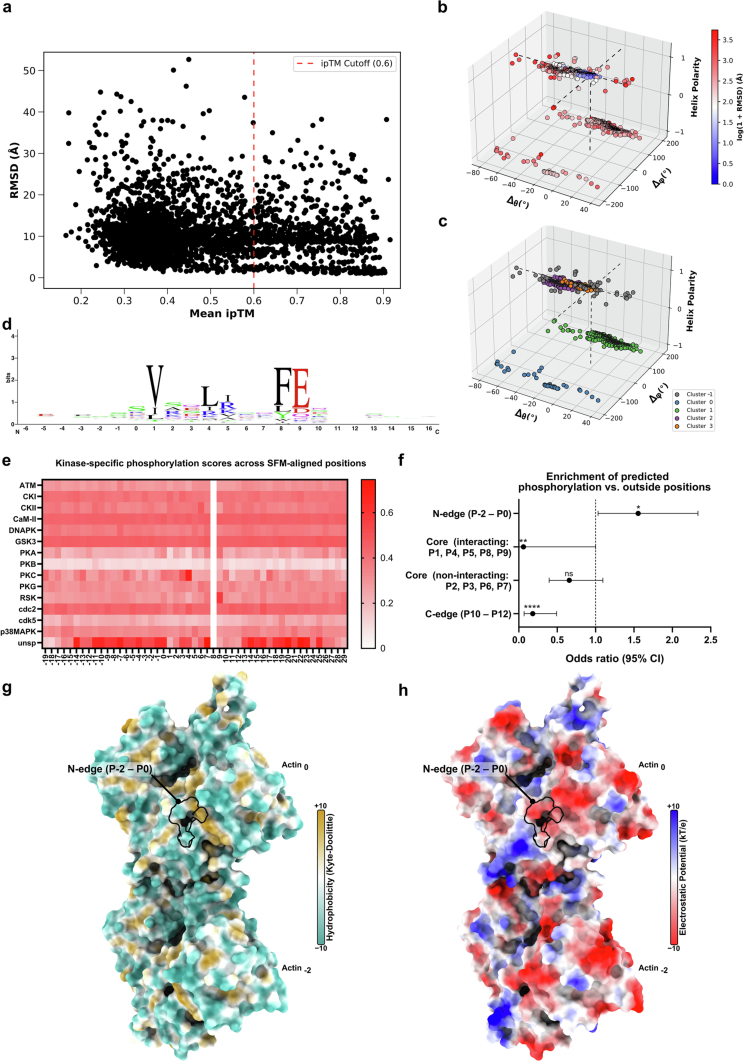
Large-scale SLiMFold pipeline results from all three iterations and predicted phosphorylation around the SFM and electrostatic context. **a**, Scatter plot of predicted peptide–actin complexes (*n* = 3,414) displaying RMSD relative to the ITPKA reference motif (*y* axis) versus mean ipTM score (*x* axis). The dashed red line at ipTM = 0.6 indicates the threshold below which predicted interfaces are typically deemed unreliable. **b**, 3D plot of Δφ, Δθ, and helix polarity for each prediction, coloured by log(1 + RMSD), illustrating the orientation distributions of all modelled peptide–actin complexes. **c**, The same 3D orientation space as in **b** but colour-coded by hdbscan clusters (0–3) and outliers (−1). Cluster 3, in particular, exhibits minimal RMSD and orientations similar to ITPKA. **d**, Sequence logo of cluster 3, highlighting the core SFM residues (P1, P4, P5, P8 and P9). The logo underscores the high sequence conservation in these positions, consistent with strong structural similarity to the ITPKA–actin reference. **e**, Heat map of the mean NetPhos serine/threonine score per kinase (rows) across relative positions. **f**, Forest plot of odds ratios (OR) with 95% confidence intervals; points denote the centre of mass and horizontal bars denote the 95% confidence interval, comparing N-edge (P − 2–P0), core (interacting) (P1/P4/P5/P8/P9),core (non-interacting)(P2/P3/P6/P7), and C-edge (P10–P12) against all other positions in the ±20-amino-acid flanks. ORs were calculated using two-sided Fisher’s exact tests on 2 × 2 contingency tables of high-confidence versus not high-confidence NetPhos serine/threonine predictions. The numbers of kinase-position observations analysed were: N-edge, *n* = 813 (inside *n* = 113, outside *n* = 700); core (interacting), *n* = 719 (inside *n* = 19, outside *n* = 700); core (non-interacting), *n* = 796 (inside *n* = 96, outside *n* = 700); and C-edge, *n* = 756 (inside *n* = 56, outside *n* = 700). No biological or technical replicates were involved, as this was a computational analysis of kinase-specific positional predictions. The vertical dashed line indicates OR = 1. Exact two-sided Fisher’s exact test *P* values were: N-edge, *P* = 0.0378; core (interacting), *P* = 0.0017; core (non-interacting), *P* = 0.1188; and C-edge, *P* < 0.0001. Asterisks denote significance by Fisher’s exact test (*P < 0.05; **P < 0.01; ****P < 0.0001; ns, not significant). **g**, Molecular surface coloured by Kyte–Doolittle hydrophobicity; the N-edge box indicates P−2–P0 of ITPKA. **h**, Molecular surface coloured by electrostatic potential (kT/e; blue = positive, red = negative). The N-edge box lies adjacent to a negatively charged (red) patch on actin, supporting the hypothesis that phosphorylation at the N-edge (adding negative charge) could electrostatically disfavour binding and modulate the interaction. Source data

Finally, we refined the PSSM of the SLiMFold pipeline by incorporating our identified SFM peptides and then performed two additional iterations to compile a definitive list of putative human SFM proteins (Fig. 7a and Extended Data Fig. 9). A total of 124 final SFM sequences emerged in 103 genes (summarized in Supplementary Table 3). Of these, 26 proteins have been tested using truncation-based approaches for the SFM^53–73^. Furthermore, 16 proteins are known to bind actin filaments through more indirect methods (for example, co-localization or co-sedimentation of the full-length protein)^74–90^. Further testing and more detailed truncation studies are needed to validate the remaining predicted proteins.

To gain insights into the biological roles of the identified SFM proteins, manual database research was performed using NCBI and PubMed. The findings were summarized in a glossary and a cartoon (Fig. 7b and Supplementary Table 4), enabling classification of the SFM peptides into distinct functional categories. These include proteins that directly modulate actin (‘actin modulators’), proteins that regulate the activity of actin-binding proteins (‘actin modulator-regulating proteins’), microtubule-binding proteins, adaptor and scaffolding proteins, junction-associated proteins and chromatin-associated proteins. The largest group comprises proteins that directly modulate actin, including well-characterized modulators such as cortactin (CTTN), caldesmon (CALD1) and supervillin (SVIL) as well as many less-studied proteins. This group is followed by actin modulator-regulating proteins, including Ras homologue family member (Rho) GTPase activators and silencers as well as Rho-associated coiled-coil-containing protein kinase (ROCK) and Slingshot phosphatase (SSH2 and -3), which regulate the activity of myosins and cofilin, respectively^91–93^. In addition, many SFM-containing proteins bind to microtubules, with a substantial portion acting as adaptors or scaffolds. In contrast, junction- and chromatin-associated proteins are less abundant. For some SFM proteins, however, clear functional categorization proved difficult, for example, Tet methylcytosine dioxygenase 3 (TET3), which is involved in DNA demethylation^94^.

In conclusion, SFMs are present in a highly diverse set of proteins, consistent with our finding that these peptides probably arose ex nihilo during evolution.

## Discussion

Actin-binding proteins are essential for the control of cellular actin dynamics; furthermore, mutation, mis-expression or deletion of ABPs can induce severe diseases^3–7^. The biological role of ABPs is defined by their actin-binding mode, and its targeting to actin can alter cytoskeletal dynamics, enzyme activity or cellular localization (reviewed in refs. ^95,96^). Here we present the discovery of the SFM.

The core SFM (V(P1)xxL(P4)xxxF(P8)E(P9)) folds into a single α-helix comprising only nine amino acids flanked by non-conserved residues. We demonstrated the essential roles of Val at position P1 and/or Phe at P8 in actin filament binding in both macrophages and H1299 cells. Shatskiy and colleagues^38^ analysed the affinity of the F-tractin F29A mutant (which corresponds to P8 of the ITPKA–SFM complex) for actin filaments. They reported a *K*_d__,app_ value of >100 µM, confirming its critical role in actin filament binding. In contrast, the residues at P4 (Leu) and P9 (Glu) seem to be less critical for actin binding. For example, SHROOM3 contains a Lys residue at P4 and USP54 contains a Gln at P9. Nevertheless, these peptides exhibit relatively low affinities for actin (*K*_d__,app_ of 11 µM and 6 µM, respectively), indicating a critical role of P4 and P9 for affinity modulation. The Lys-to-Leu mutation at P4 in SHROOM3 and Gln-to-Glu mutation at P9 in USP54 significantly increased their respective actin-binding affinity. These findings suggest that amino acids at P4 and P9 primarily modulate the binding strength of SFM peptides to actin filaments. However, our data indicate that affinity modulation is not solely controlled by P4 and P9 given that ESPNL, despite exhibiting a relatively low *K*_d__,app_ of 6 µM, contains the conserved Leu or Glu at these positions. Because ESPNL features an additional helix at the N terminus of the peptide, it is tempting to speculate that the insertion of extra helices may also modulate the affinity of SFMs to actin. However, future experiments are required to validate this assumption.

The direct interactions between the SFM amino acids and actin may be regulated by phosphorylation given that SLiMs often contain regulatory post-translational modifications^97^. Consistent with this, NetPhos analysis revealed Ser/Thr phosphorylation enrichment at the N-terminal edge of the SFM (P−2–P0). In line with this, many SFM sequences contain Ser/Thr residues clustered in the immediate vicinity of the hydrophobic anchor at P1, providing candidate sites for tuning the local interaction strength. In our USP54 M1 structure, Ser7 (P0) forms a hydrogen bond with actin Asp25 within a negatively charged patch, which suggests that phosphorylation at this site could electrostatically weaken or abolish the interaction and fine-tune SFM–actin binding (Extended Data Fig. 9).

Evolutionary analysis provided further support for the functional importance of the conserved positions P1, P4, P8 and P9 within the SFM for actin-filament-binding strength, as these residues are maintained across diverse species (Fig. 6b). Moreover, a low dN/dS ratio^52^ at these positions evidenced purifying selection and a strong evolutionary drive to retain functional actin filament-binding capacity. Moreover, phylogenetic analysis indicated that the SFM emerged spontaneously (ex nihilo) rather than via exon shuffling or domain duplication. It is probable that the motif randomly developed from point mutations and given that it provided a new functionality it was negatively selected. This ex nihilo development seems to be typical for SLiM evolution, among which classical examples are nuclear import and export motifs as well as the calmodulin-binding site^98^.

**Extended Data Fig. 10:**
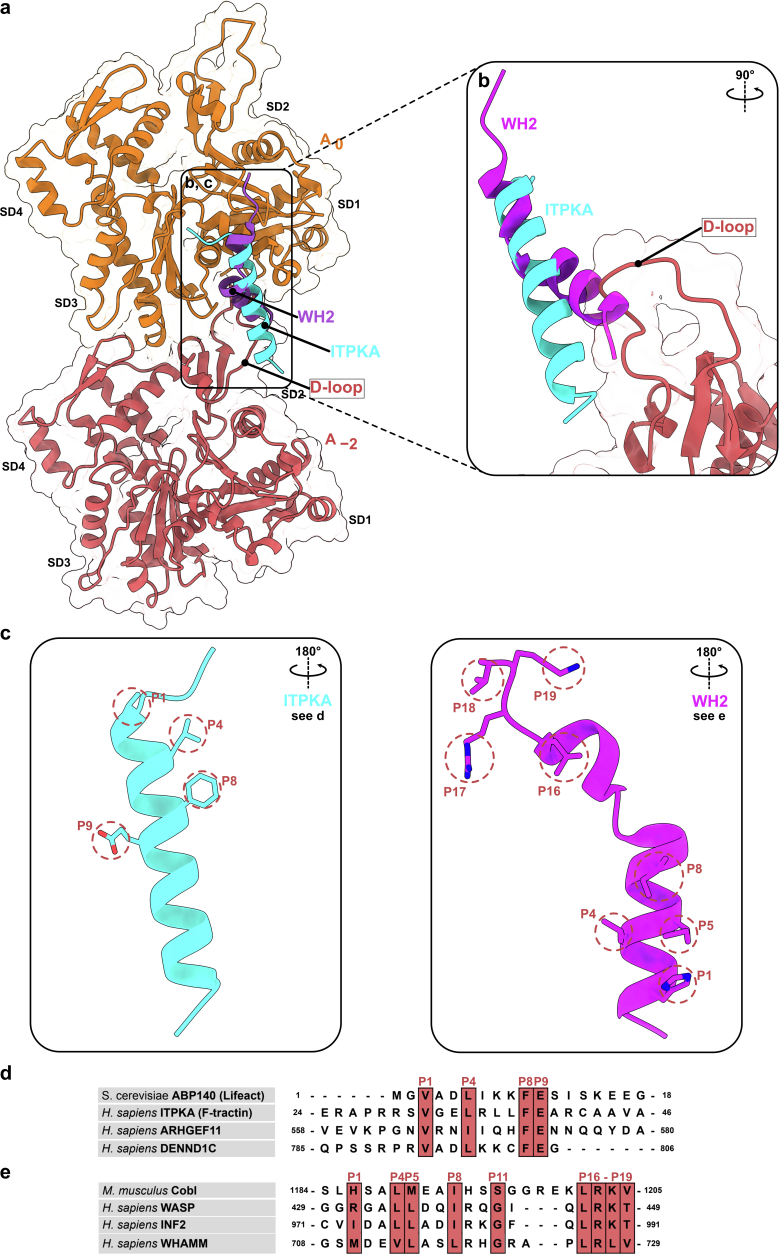
Structural and sequence comparison of the SFM with the canonical WH2 motif. **a**, Overall view of actin with subdomains (SD1–SD4) labelled. The SFM helix from ITPKA (cyan) and a WH2 helix (magenta, PDB ID: 6JCU) bind in the barbed-end groove adjacent to the D-loop. The dashed box marks the region enlarged in **b**,**c**. **b**, Close-up (rotated 90°) showing that the two helices occupy overlapping surfaces, while the WH2 would sterically clash with the D-loop of the underlying actin subunit (A_−2_), whereas the SFM trajectory avoids this clash. **c**, Side-chain views highlighting key positions (dashed circles). Left: ITPKA–SFM with conserved anchors at P1, P4, P8, P9. Right: WH2 with canonical positions P1, P4, P5, P8 and a C-terminal basic segment (P16–P19). In this view, the WH2 helix is oriented antiparallel relative to the SFM. **d**, Sequence alignment of SFM-type binders (for example, ABP140/Lifeact, ITPKA/F-tractin, ARHGEF11, DENND1C) mapped to the SFM index; red boxes mark conserved P1/P4/P8/P9. **e**, Sequence alignment of representative WH2 motifs (Cobl, WASP, INF2, WHAMM), highlighting the WH2 pattern (P1/P4/P8/P11) and the P16–P19 basic stretch.

However, the WH2 motif, which also belongs to the SLiM family, evolved from an ancestral protein and thus did not develop ex nihilo^99^. Like the SFM, the motif binds to the hydrophobic groove between SD1 and SD3 but it primarily interacts with monomeric actin. The WH2 domain adopts an opposite-polarity orientation compared with ITPKA, with its N terminus approaching the D-loop—an arrangement that is unfavourable for filament decoration^100^. By contrast, the SFM utilizes conserved anchor points (P1, P4, P8 and P9) in a geometry that avoids D-loop clashes, supporting filament binding (Extended Data Fig. 10). Consequently, the SFM and WH2 domains constitute distinct actin-binding solutions, specialized for filamentous and monomeric actin, respectively. Hence, a direct competition between the ITPKA/Lifeact and WH2 domains for actin filament binding is unlikely.

Notably, in addition to the SFM and the WH2 domain, many conserved globular actin-binding domains also bind to the hydrophobic cleft between SD1 and SD3 of actin, that is, the ABDs of vinculin and α-catenin^101,102^. They are related and consist of short α-helices arranged into compact bundles. Helices 4 of vinculin and α-catenin mediate hydrophobic interactions within SD1 of actin, involving residues Ile345 and Ile341, which also interact with conserved amino acids within the SFM^101^. The calponin-homology (CH) domains found in many actin-bundling and crosslinking proteins (Supplementary Table 1) are similarly comprised of short α-helices connected by flexible linkers. For instance, the CH3–CH4 domain of L-plastin binds to SD1 through apolar and polar contacts; and, notably, L-plastin and the ITPKA–SFM complex interact with the same residues in SD1 of actin (Ile341, Ile345, Leu349 and Ser350)^102^.

Moreover, according to the cryo-EM structure, cofilin-1 also interacts with residues Ile341, Ile345 and Leu349 within the SD^103^. Here the cofilin-helix shows the same orientation inside the hydrophobic groove as the SFM-peptide^40^, and the overlapping binding sites may explain why Lifeact competes with cofilin-1 for binding to actin filaments^9^. Importantly, both cofilin-1 and ITPKA also soften actin filaments^50^ (Fig. 5). Cofilin-1 reduces the *L*_p_ of actin filaments approximately 4.4-fold, most probably by inducing conformational changes in the D-loop of SD2, thereby facilitating filament severing^50^. In contrast, ITPKA and USP54 M1 decrease the *L*_p_ by only approximately 1.5–1.8-fold. Whether Lifeact also alters the actin filament *L*_p_ or the conformation of the D-loop remains unresolved as MD simulations of the Lifeact–actin filament complex are not yet available. A comparison with MD simulations of undecorated actin showed that the flexible D-loop samples a broad range of conformations, including the one observed in the Lifeact-decorated structure (Extended Data Fig. 8). However, our RMSD analysis revealed that ITPKA binding stabilizes the D-loop in a state that is structurally closer to the Lifeact reference and significantly more rigid than actin alone (Extended Data Fig. 8). We propose that the pronounced D-loop rigidification induced by ITPKA reflects the greater length of its SFM compared with Lifeact and USP54 M1, enabling more extensive interactions with D-loop residues (Extended Data Fig. 8). However, definitive comparison of D-loop modulation by Lifeact, ITPKA and USP54 M1 will require future MD simulations of Lifeact-bound actin filaments. Such studies could also extend sampling timescales to capture the full equilibration from the static cryo-EM conformation to the physiological ‘warm’ actin ensemble described recently^104^. More broadly, determining whether additional SFM peptides modulate actin filament *L*_p_ will be essential to establish whether this effect represents a general property of SFM binding or is restricted to specific SFM peptides.

Interestingly, our SLiMFold prediction suggests that the small SFM helix is present in 103 highly diverse proteins, of which actin binding has been experimentally validated for 26 SFMs (Supplementary Table 3). Although we did not investigate the biological roles of the identified SFM-containing proteins, previously reported cellular functions for most of them (summarized in Supplementary Table 4) allowed us to classify them into functional categories: proteins that directly modulate actin, proteins controlling the activity of actin modulators, microtubule-binding proteins, adaptor or scaffolding proteins and chromatin-remodelling proteins. Among these groups, the SFM-containing proteins involved in actin regulation encompass the highest number of proteins. These include ABPs known to bind to actin via canonical actin-binding motifs, and we assume that the additional SFM actin-binding site has a synergistic or additive effect on proteins targeted to actin by canonical ABDs. It has been shown for cortactin that, in addition to the cortactin repeats, an adjacent linker, which includes the SFM, is required to mediate high affinity binding to actin filaments^57^. Given that cortactin stabilizes Arp2 and -3 actin-filament branches, this activity may be important for the maintenance of cellular protrusions. In addition, the caldesmon (CALD1) SFM was identified and it was shown that its binding to actin can be displaced by calmodulin, indicating that SFM-mediated actin binding is weakened after cellular stimulation^55^. A synergistic or additive effect of the SFM on actin binding may also work in proteins containing SFM repeats, such as pleckstrin homology and RhoGEF domain-containing G3 (PLEKHG3) whose SFM-deletion abrogates cell polarity of 3T3 fibroblasts^66^. Moreover, depletion of the SFM-based actin-binding site in the RhoGEF ARHGEF11 disrupts the balance between Ras-related C3 botulinum toxin substrate (Rac) and Rho signalling, impairing the formation of leading-edge protrusions and trailing-edge retractions in A431 cancer cells^105^. Similarly, SFM repeats are present in the adaptor proteins sorbin and SH3 domain-containing 1 and 2 (SORBS1 and 2) as well as the cofilin-regulating phosphatase SSH2 (Supplementary Table 3).

However, ITPKA contains only a single SFM, yet its deletion severely disrupts actin architecture and impairs invasion of lung cancer cells^37^. This demonstrates that SFMs do not require clustering in repeats to mediate actin filament targeting. Moreover, the SFM of ITPKA is involved in regulation of signal transduction. Deletion of the ITPKA–SFM complex significantly reduces the InsP_3_Kinase activity of ITPKA^36^ and thereby alters calcium signalling in hippocampal neurons and cancer cells^32,34^. Similarly, the cancer-associated adaptor protein Pseudopodium-enriched atypical kinase (PEAK) is targeted to actin and recruits paxillin to focal adhesions, which suggests that its actin localization helps to coordinate focal adhesion architecture following integrin activation^104^. Furthermore, paxillin itself contains an SFM and recruits vinculin and actopaxin to focal adhesion sites^106^. Moreover, deletion of the zonula occludens-1 (ZO-1 or TJP1) SFM alters the barrier function in kidney MDCK cells, indicating that the SFM exhibits an important function in connecting the actin cytoskeleton with cellular junctions^72^.

However, no studies have investigated SFM-mediated actin binding for SFM proteins involved in microtubule regulation or chromatin modification. These groups include proteins with broad cellular functions, such as kinesin family member 21B and 23 (KIF21B and KIF23), as well as the histone methyltransferase SET domain-containing 3 (SETD3). For microtubule-associated proteins, SFM-mediated targeting to actin filaments may provide a physical link between the actin and microtubule cytoskeleton. In chromatin-associated proteins, SFM-mediated actin targeting could serve as a scaffold. Future studies dissecting the biological roles of SFM–actin interactions will be essential to test these hypotheses.

Finally, two central questions remain for future studies. First, does the SFM provide a broad cellular network? Second, does it define local molecular circuits refined during evolution to adapt to environmental changes? Given that the SFM may have developed ex nihilo, the second hypothesis is more plausible.

In conclusion, SFM-mediated actin filament targeting regulates the properties of diverse cellular proteins, and future studies will probably uncover additional, as-yet-unidentified biological roles. Moreover, expanding our motif search to include non-human databases could reveal the conservation and functional diversity of SFMs across different species, providing deeper evolutionary insights and potentially identifying novel SFM-containing proteins. Looking forward, we envision that the elucidation of the biological role of SFM proteins will uncover cellular regulation mechanisms involved in physiological and pathological settings.

## Methods

### Pipeline overview

The SLiMFold pipeline, which integrates multiple bioinformatic tools to systematically identify, filter and validate candidate motifs was used for the identification of actin filament-binding SLiMs. Among others, the SLiMFold pipeline integrates ColabFold^45^, which utilizes AlphaFold2 Multimer to predict the structures of identified actin-binding candidates. To increase the specificity and accuracy of the pipeline, actin filament-binding SLiMs were identified by running three iterations, each involving the followings steps.

#### Prerun

The process begins with Prerun.ipynb, where hypothesized SLiMs (in this study, the aligned sequences of ITPKA and Lifeact ABDs from P1 to P9) were used along with the BLOSUM62 substitution matrix^107^ to create a PSSM. This PSSM was used to scan the human proteome (NCBI Taxonomy ID: 9606) for putative motif hits. To further filter out false positives, we incorporated the following criteria.

- PSSM score: The matrix was used to score sequences in the database, identifying motifs similar to the hypothesized SLiMs^108^.
- IUPRED: Predicts intrinsically disordered regions, highlighting regions suitable for motif embedding. The mean IUPRED score was calculated for the motif and its 60-residue flanking regions (N- and C-terminal)^43^.
- ANCHOR: Identifies regions in intrinsically disordered regions that are likely to bind structured partners. The mean ANCHOR value was calculated within the motif^42^.
- PSIPRED: Predicts the probability of secondary structures (helix, β-strand and random coil)^41^.

All cutoffs for these criteria were progressively relaxed across iterations (described in the next paragraph). This approach allowed the identification of additional hits while iteratively strengthening the PSSM, reducing false positives. Each hit was extended by ±20 flanking residues for subsequent homology searches and redundant sequences were removed. Each hit was then paired with the bait sequence (the human actin sequence) and saved as a separate FASTA file, formatted for direct compatibility with ColabFold.

For each hit, MSAs were generated using jackhmmer^44^ with the UniRef90 database. The following jackhmmer parameters were applied: five iterations; *e*-value = 1 × 10^−5^; no f1 or f2 filter (original AlphaFold2 code: one iteration; *e*-value = 1 × 10^−5^; f1, 0.0005; f2, 0.00005). The modified jackhmmer parameters improved the number of sequences retrieved, probably enhancing detection of more distant homologues. In addition, parallelization was implemented to process multiple hits simultaneously, significantly speeding up the alignment step. The sto-file for the bait sequence was constructed in the same manner. Output alignments were reformatted to A3M format using the reformat.pl script in HH-suite^109^ to ensure compatibility with downstream structure prediction tools. Alignments were sorted and combined to meet AlphaFold2 Multimer input criteria.

#### ColabFold looped

The ColabFold_looped.ipynb notebook, originally developed by the Steinegger laboratory^45^, was modified to enable batch processing and the integration of custom MSAs, making it more suitable for the SLiMFold workflow. These modifications were designed to streamline the process and enhance flexibility when handling multiple input sequences. In the modified version, the notebook was adapted to loop through all FASTA files in a specified folder, such as one located in Google Drive. This automation allowed for seamless processing of multiple sequences without the need for manual input, significantly improving the workflow efficiency. In addition, the modified notebook incorporated the ability to retrieve precomputed A3M files from another designated folder. These alignments, created during the Prerun.ipynb step, were matched to their corresponding FASTA files based on their names. This ensured accurate integration of the custom MSAs into the ColabFold predictions. To manage outputs effectively, the notebook allowed users to specify a folder for storing results. This organizational set-up made it easier to handle and analyse the results of large-scale computations.

Compared with the original ColabFold batch notebook, these modifications introduced enhanced flexibility by supporting custom MSAs and allowing adjustments to the number of seeds used in the AlphaFold2 Multimer predictions.

#### Post analysis

The post-analysis step was performed using the Postanalysis.ipynb script, which systematically processed and analysed the outputs from the SLiMFold pipeline. This step integrated sequence and structural data to evaluate the conformational and functional relevance of candidate actin filament-binding SLiMs. The pLDDT, pTM and ipTM scores were extracted to assess prediction confidence and interaction reliability of each predicted structure. Structures with ipTM scores below 0.6 were excluded from further analysis, ensuring a focus on high-confidence predictions. Each remaining structure was aligned to a reference PDB file (in this study, the ITPKA–actin complex, PDB: 9QGK) to evaluate conformational similarity.

The RMSD between the alpha carbon (Cα) atoms of the predicted structure and the reference structure at the motif positions (P1 to P9) was calculated to quantify structural similarity. RMSD was computed as:

$${\rm{RMSD}}=\frac{1}{N}\displaystyle \mathop{\sum }\limits_{i=1}^{N}{({x}_{i}-{x}_{{\rm{ref}}})}^{2}$$

where *N* is the number of aligned residues, ${x}_{i}$ represents the atomic coordinates of a residue in the predicted structure and ${x}_{{\rm{ref}}}$ represents the corresponding coordinates in the reference structure.

Angular analysis was performed using vector-based calculations, where the spatial coordinates of the alpha carbon (Cα) atoms of key residues in the SLiM (P1 to P9) were used to define vectors representing the orientation of the motif. Two angles, $\varphi$ (azimuthal angle in the *x*–*y* plane) and $\theta$ (polar angle relative to the *z* axis), were calculated to quantify the orientation of the motif relative to the reference vector:

$$\varphi =\arctan \left(\frac{y}{x}\right)\quad\theta =\arccos \left(\frac{z}{{|v|}}\right)$$

where $x$ and $y$ are the coordinates of the vector projection in the *x*–*y* plane, $z$ is the *z*coordinate of the vector and |$v$| is the magnitude of the vector. Helix polarity was also calculated to capture the directionality of the structural alignment of the predicted motif. The Δ*φ* and Δ*θ* values, representing the differences between the angles of the predicted and reference structures, were calculated for each candidate SLiM. These metrics, along with RMSD and polarity, provided a comprehensive structural profile for clustering.

Clustering was performed using the hdbscan^46^ algorithm, leveraging RMSD, Δ*φ*, Δ*θ* and polarity as input features. The clustering parameters, including minimum cluster size and minimum samples, were optimized using the following metrics.

- Silhouette score: Evaluates cluster separation and cohesion.
- Davies–Bouldin index: Measures intra-cluster similarity relative to inter-cluster separation.
- Calinski–Harabasz index: Assesses the ratio of between-cluster dispersion to within-cluster dispersion.

Optimal clustering parameters were selected based on these indices, ensuring robust and meaningful grouping of structurally similar SLiMs. For further analysis, all structures belonging to a single cluster were exported as a PyMOL session file (.pml), enabling manual inspection and visualization. For each cluster, the corresponding sequences were compiled into a FASTA file. These sequences were used to generate sequence logos^110^, highlighting conserved residues and identifying potential functional motifs. In addition, each sequence was mapped to its corresponding gene using the NCBI database. This mapping facilitated gene ontology (GO) enrichment analysis, allowing functional insights into the biological roles of the identified SLiMs.

### Cloning strategies

All constructs were generated via PCR-based cloning using high-fidelity polymerases (Q5 or Phusion; M0491S or M0530S, New England Biolabs Inc.) following the manufacturer’s instructions. For inserting longer DNA fragments such as full-length protein sequences, we employed T4 sequence and ligation independent cloning^111^ (T4 DNA Polymerase; M0203S, New England Biolabs Inc.), which relies on overlaps of about 30 nucleotides between the vector and insert for seamless assembly. Short peptides or specific mutations were introduced using a ‘QuickChange-like’ site-directed mutagenesis protocol, wherein primer pairs incorporated short (8–12 nucleotides) overlaps at their 5′ ends; the PCR products were subsequently treated with DpnI (R0176S, New England Biolabs Inc.) to remove the template and then transformed into *Escherichia coli* XL1-Blue (200249, Agilent Technologies, Inc.). Alternatively, the PCR products were processed using a kinase–ligase–DpnI enzyme mix (M0554S, New England Biolabs Inc.) to remove the template and ligate the new amplicon. For bacterial expression, PCR products were cloned into pSF421-based expression vectors (for example, pSF421_10xHis_GFP_TEV); for eukaryotic expression in mammalian cells (for example, H1299), target genes were inserted into an mEGFP-N1 backbone. All final plasmids were verified by Sanger sequencing (Microsynth Seqlab GmbH). Verified constructs were subsequently transformed into *E. coli* Rosetta (DE3) cells (Novagen, Merck KGaA) for bacterial protein expression or transfected into mammalian cells. Complete plasmid lists, vector maps, sequencing results and primer sequences are provided in Supplementary Tables 5,6 and Source data. The oligonucleotides used in this study were designed in-house and synthesised by Integrated DNA Technologies (IDT).

### Actin purification

Actin was prepared from *Gallus gallus* (chicken) skeletal muscle as described^112^. The final purification step was performed using a HiLoad 16/600 Superdex 200 column equilibrated with globular actin buffer (5 mM Tris–HCl pH 7.5, 0.2 mM CaCl_2_, 0.5 mM dithiothreitol and 0.2 mM ATP).

### Expression and purification of ITPKA and GFP peptides

All constructs were expressed in *E. coli* Rosetta (DE3) competent cells (Novagen, Merck KGaA). The cells were cultured in Terrific Broth at 37 °C to an optical density at 600 nm of 0.6–1.0. Protein expression was induced with 0.2 mM isopropyl β-D-1-thiogalactopyranoside and incubation at 18 °C for 16 h for ITPKA or at 37 °C for 4 h for the GFP-tagged peptides. After induction, the cells were harvested by centrifugation at 3,000*g* and 4 °C for 20 min. The cell pellets were resuspended in cold PBS buffer, centrifuged at 2,000*g* and 4 °C for 30 min, flash-frozen in liquid nitrogen and stored at −80 °C.

For protein purification, the cell pellets were resuspended in ice-cold Buffer A (50 mM Tris–HCl pH 7.4, 400 mM NaCl, 3 mM MgCl_2_ and 1 mM β-mercaptoethanol for ITPKA; 50 mM Tris–HCl pH 7.4, 300 mM NaCl and 1 mM β-mercaptoethanol for GFP peptides) and homogenized using an IKA ULTRA-TURRAX disperser (IKA-Werke GmbH & Co.). DNase I was added and the cells were lysed with a Constant Cell Disruption System (Constant Systems Limited) at 1.8 kbar. Following lysis, phenylmethylsulfonyl fluoride (final concentration of 1 mM) and imidazole (final concentration of 25 mM) were added to the cells. The cell debris was removed by centrifugation at 43,000*g* and 4 °C for 30 min, and the supernatant was incubated with Ni-NTA agarose resin (SERVA Electrophoresis GmbH) for 30 min at 4 °C. The bound proteins were eluted using a gradient of Buffer B (Buffer A with 500 mM imidazole). Fractions were analysed by SDS–PAGE, and those with >90% purity were pooled and then dialysed overnight at 4 °C in Buffer A.

Subsequently, the dialysed proteins (GFP peptides) were concentrated to 0.5–2 ml using Amicon Ultra Centrifugal Filters (Merck-Millipore) and further purified by size-exclusion chromatography using a HiLoad Superdex 16/600 200 pg Gel Filtration Column (Cytiva) on an NGC liquid chromatography system (Bio-Rad Laboratories) equilibrated with Buffer D (50 mM Tris–HCl pH 7.4, 150 mM NaCl, 3 mM MgCl_2_ and 1 mM β-mercaptoethanol for ITPKA; 50 mM Tris–HCl pH 7.4, 150 mM NaCl and 1 mM β-mercaptoethanol for GFP peptides). The peak fractions were concentrated, aliquoted and then flash-frozen in liquid nitrogen, and stored at −80 °C.

### Acquisition and processing of cryogenic electron microscopy data

We initiated all sample preparations using frozen aliquots of globular actin. To convert globular actin into filamentous actin, the samples were first thawed, followed by centrifugation at 100,000*g* for 30 min to eliminate potential aggregates. The polymerization process involved the addition of F buffer (20 mM Tris-HCl pH 7.5, 100 mM KCl, 2 mM MgCl_2_, 0.5 mM ATP, 1 mM EGTA, 1 mM dithiothreitol), followed by incubation at room temperature for 1 h. Subsequently, we collected the filaments by centrifugation at 100,000*g* and 4 °C for 1 h, and resuspended them in F buffer supplemented with phalloidin at twice the molar concentration of the actin filaments.

Quantifoil 200-mesh 2.0/1.0 holey carbon grids were used for cryo-EM grid preparation. Specifically, for phalloidin-stabilized actin filaments, we applied 3.5 μl of 1 μM solution onto glow-discharged grids. For the phalloidin-stabilized actin filament–ITPKA complex, we initially applied 2 μl of 1 μM phalloidin-stabilized actin filaments to the grids. Subsequently, we added 2 μl of 10 μM ITPKA and mixed it directly on the grids immediately before plunge-freezing in a liquid ethane–propane mixture using a Vitrobot Mark IV system (FEI). Datasets were collected on an FEI Titan Krios transmission electron microscope (300 kV; Gatan K3 camera; pixel size, 0.826 Å; dose per frame, 1.4; defocus range, 0.8–3.0 μm).

Single-particle helical reconstruction was performed using Relion 4 (ref. ^113^). Videos were first motion-corrected using MotionCor2 (ref. ^114^) and then the contrast transfer function estimation was performed using CTFFIND4 (ref. ^115^). Particle segments picking model was trained in cryolo 1.7 (ref. ^116^). The helical segments were extracted into 360 × 360 boxes and the junk segments were excluded after two-dimensional classification. The initial helical parameters of a helical rise of 27.3 Å and a helical twist of −166.5° were applied for three-dimensional classification and autorefinement in Relion 4.0. Overall, gold-standard resolution (Fourier shell correlation = 0.143) was calculated in Relion 4.0. The statistics for data collection and processing are listed in Supplementary Table 2.

For the actin filament–USP54 M1 complex, actin filaments were prepared without phalloidin. A total of 2 μl of 4.75 μM actin filaments were applied to glow-discharged grids, followed by 2 μl of 105.83 μM USP54 M1–EGFP fusion protein. The components were mixed directly on the grids immediately before plunge-freezing in liquid ethane using a Vitrobot Mark IV. Data were collected on a Glacios transmission electron microscope operated at 200 kV and equipped with a Falcon 4i camera, an energy filter with a 10 eV slit width and a 50 μm C2 aperture. Images were recorded at a calibrated pixel size of 0.91 Å, with a total accumulated dose of 40 e^−^ Å^−^^2^ and a defocus range of –0.5 to –2.0 μm. Motion correction, contrast transfer function estimation, particle picking, two-dimensional classification and helical refinement were performed in CryoSPARC^117–121^. Helical segments were identified using the Filament Tracer tool, and non-specific particles were removed by two-dimensional classification. The remaining segments were further refined through heterogeneous classification, with the best three-dimensional class subjected to final helical refinement. The resolution of the reconstruction was determined by gold-standard Fourier shell correlation at the 0.143 criterion. A summary of this data collection and image processing statistics is provided in Supplementary Table 2.

### Model building and refinement

Previously published models of phalloidin-bound actin filaments (PDB: 6T20, ref. ^122^; 7BTI ref. ^40^) and the AlphaFold2 structure of ITPKA and USP54 M1 SFM were used as initial models. Models were initially built in ChimeraX 1.7 (ref. ^123^), and further refined against the cryo-EM maps using ISOLDE^124^ and real space refinement in Phenix^125^. The detailed model information and validation statistics for final models are described in Supplementary Table 2.

### Cell culture

NCI-H1299 (H1299) cells were provided by C. Günes (Hamburg, Germany). For detailed cellular characteristics, refer to the American Type Culture Collection. Cell line authentication was performed by the Leibniz Institute DSMZ–German Collection of Microorganisms and Cell Cultures (28 January 2025) using analysis of 17 short-tandem-repeat loci. The cells were cultured in Dulbecco’s modified eagle’s medium supplemented with 10% (vol/vol) fetal calf serum, 4 mM L-glutamine, 100 μg ml^−1^ streptomycin and 100 U ml^−1^ penicillin. The culturing conditions were rigorously maintained to ensure optimal growth and viability. In addition, the cells were tested regularly for mycoplasma contamination to ensure the experiments were conducted with mycoplasma-free cells.

Primary human monocytes were isolated from buffy coats (provided by F. Bentzien, Transfusion Medicine, UKE, Hamburg, Germany). A 20 ml volume of blood was coated on 15 ml lymphocyte separation medium 1077 (PromoCell) and centrifuged at 4 °C and 460*g*for 30 min. The buffy coats were transferred to a new 50 ml Falcon tube and made up to 50 ml with cold RPMI (Gibco). Leukocyte fractions were washed twice in RPMI and centrifuged for 10 min, as described earlier. Enriched leukocytes were resuspended in 400 μl monocyte buffer (5 mM EDTA and 0.5% human serum albumin in Dulbecco’s PBS, pH 7.4), mixed with 100 μl of magnetic beads suspension coupled to antibodies to CD14 (Miltenyi Biotec) and incubated on ice for 15 min. The mixture was subsequently loaded onto Separation columns LS (Miltenyi Biotec) that had been previously placed in a magnetic holder and equilibrated with 500 μl cold monocyte buffer. CD14^+^ monocytes trapped in the column were washed with 500 μl monocyte buffer and then eluted with 1 ml monocyte buffer into 15 ml cold RPMI after removal from the magnets. After centrifugation at 460*g*and 4 °C for 10 min, the supernatant was removed, and the cells were resuspended in 40 ml RPMI and seeded on a six-well plate (Sarstedt) at a density of 2 × 10^6^ cells per well. The monocytes were allowed to adhere for 1 h, after which the RPMI medium was replaced with 2 ml monocyte culture medium (RPMI substituted with 20% human serum and 1% penicillin–streptomycin; Sigma-Aldrich). The monocytes were cultured in an incubator at 37 °C, 5% CO_2_ and 90% humidity. Isolated monocytes were differentiated for at least six days.

### Transfection of cells

For the H1299 cells, a total of 2.5 × 10^4^ cells were seeded into eight-well chamber slides (Ibidi) and incubated for 16 h. The cells were then transfected with 0.5 µg of any mEGFP-N1-Peptide or mEGFP-N1-FL-Proteins using K2 transfection reagent (T060-0.75, Biontex Laboratories GmbH) according to the manufacturer’s instructions. After 24 h, the cells were fixed with 4% paraformaldehyde in 4% sucrose, stained with rhodamine-conjugated phalloidin (ab235138, Abcam Limited) and analysed by fluorescence microscopy using an Olympus IXplore Live microscope imaging system and FV3000 confocal laser-scanning microscope.

Macrophages were detached by incubation with accutase (Invitrogen) for at least 30 min in culturing conditions. The cells were collected with monocyte culture medium, washed in PBS pH 7.3 and resuspended in R Buffer (at a concentration of 1 × 10^6^ cells per 100 µl buffer, 10 µg DNA), provided by the Neon Transfection System (Invitrogen). The macrophages were transiently transfected with plasmid DNA with the following settings: voltage, 1,000 V; width, 40 ms; two pulses. The transfected cells were resuspended in RPMI and seeded on 12-mm glass coverslips (1 × 10^5^ cells per coverslip). The cells were left to adhere for 1 h under culturing conditions. Thereafter, 1 ml of monocyte culture medium was added to the cells, followed by overnight incubation.

### Immunofluorescence and microscopy

Cells were fixed in PBS containing 3.7% formaldehyde for 10 min, followed by permeabilization in PBS containing 0.5% Triton X-100 for 10 min. Thereafter, the cells were incubated for 60 min in blocking solution (2% BSA in PBS) with 1:400 phalloidin-568. The cells were washed three times in PBS and mounted on glass slides with FluoromountG (Invitrogen) containing 4′,6-diamidino-2-phenylindole (Sigma-Aldrich). Images of fixed samples were acquired using an Olympus FV3000 equipped confocal laser-scanning microscope with an ×60 UPlanApo HR oil objective and the Olympus FV3000 software.

### Poji macro analysis

Localization of peptides to actin filaments at podosomes was evaluated using Poji^126^. The Poji macro is a semi-automated ImageJ/Fiji plugin created to characterize protein distribution and enrichment at podosomes. Profile analysis begins with the processing of raw microscopy data as separate fluorescence channels, with the actin filament channel, defined by phalloidin-568 staining, as a reference. To enable good detection quality at the ventral surface of transfected cells, prominence was set to 100 pixels and the detection size of the podosomal circular region of interest (ROI) to 25 pixels. These parameters were kept through all the experiments and cells analysed. At least three cells were analysed simultaneously to reduce the workload. During analysis, fluorescence intensities of 360° intensity profiles of single podosomal ROIs were measured.

Poji generates a circular ROI around each analysed podosome, with subsequent stacking of individual podosomal ROIs to create average intensity profiles and rotational line scans. The Poji radial profiles at optical *z* plane of highest actin filament intensity, with mean ± s.d. values of the fluorescence intensity of actin filament and the respective SFM peptides, were determined for *n* ≥ 3 cells, with 240 podosomes per transfected peptide.

### Co-sedimentation assay and apparent *K*_d_ determination

The *K*_d,app_and binding strength of the interaction between the ABP and filamentous actin were determined using an actin filament co-sedimentation assay.

For this, preparations were made using frozen aliquots of globular actin. To convert globular actin into actin filaments, the samples were thawed and centrifuged at 100,000*g* for 30 min to eliminate potential aggregates. For actin polymerization, F buffer (see above) was added and the samples were then incubated at room temperature for 1 h. The resulting actin filaments were mixed with 4 µM EGFP–SFM peptide and incubated for 1 h at room temperature before co-sedimentation at 100,000*g* for 30 min. Thereafter, the pellet was resuspended in sample buffer, heated to 95 °C for 15 min and analysed by western blotting employing an antibody to EGFP. We then plotted densitometry (*D*) as a function of the total actin concentration ${[{\rm{actin}}]}_{{\rm{total}}}$. These data were fit in GraphPad Prism using the ‘One-site specific binding’ hyperbolic model:

$$D=\frac{{{B}_{{\mathrm{max}}}\times [\mathrm{actin}]}_{\mathrm{total}}}{{K}_{{\rm{d}}}+{[\mathrm{actin}]}_{\mathrm{total}}}$$

All experiments were performed in triplicate to ensure reproducibility. EGFP–SFM peptide (fixed concentration of 4 µM) was incubated with Varying concentrations of actin (0–200 µM). In each replicate, a negative control (0 µM actin) verified background signal.

For the binding strength determination of USP54 and SHROOM3 mutants (M1, M2, M3 and M4), the proteins were employed at a final concentration of 4 µM and actin filaments at 12 µM. Densitometric analysis was performed on the western blot results, quantifying the signal intensity of the bound EGFP–SFM peptide in the pellet fraction to assess differences in binding strength among the mutants. The densitometry values were corrected by subtraction of the negative control (SFM with 0 µM actin).

As a specificity control, EGFP alone as well as the Lifeact P1/P8 constructs were used (Extended Data Fig. 6).

### SDS–PAGE and western blotting

Equal volumes of samples were loaded onto 12% polyacrylamide gels for SDS–PAGE. Electrophoresis was performed at 120 V until the dye front reached the bottom of the gel. The separated proteins were transferred onto nitrocellulose membranes using a wet transfer system at 60 V for 90 min in 1×blot buffer. Non-specific binding was blocked by incubating the membranes in 5% (wt/vol) non-fat dry milk in Tris-buffered saline with Tween 20 (TBST) for 1 h at room temperature. The membranes were incubated overnight at 4 °C with the primary antibody specific to EGFP (mouse anti-EGFP, 1:1,1000; catalogue number 11814460001, Roche Applied Science) diluted in blocking buffer. After three washes with TBST, the membranes were incubated with secondary antibody for EGFP detection (goat anti-mouse; 1:10,000 dilution in TBST) at room temperature for 1 h. Protein bands were detected using a chemiluminescence reagent (Cytiva Amersham ECL prime western blot detection reagent) and imaged using an INTAS ECL chemocam system (INTAS Science Imaging Instruments GmbH). Band intensities were analysed using ImageJ^127^ with consistent ROIs applied across all lanes and local background subtraction performed individually. Linearity of the detection range was verified using a calibration curve to ensure the measurements remained within the dynamic range of the assay. Western blot detection was chosen instead of Coomassie staining to enable sensitive detection of EGFP–SFM peptides. The *K*_d,app_ values are therefore comparable within this experimental series but should not be directly compared with values obtained using alternative detection methods.

### Position-specific frequency matrix

We developed a pipeline for generating PSFMs that quantify amino acid conservation at each position across a set of sequences, focusing specifically on peptides with high affinity or strong co-localization. Multiple sequence alignments derived from SLiMFold’s jackhmmer output were manually curated to remove alignments with names indicating different proteins, ensuring retention of only true homologues. These curated MSAs served as input for generating individual frequency tables for each peptide, which were then averaged to account for variations in the number of homologous sequences. A 23 × *N*matrix (where *N* is the sequence length) was generated, with each element representing the proportion of a specific amino acid at a given position. To reduce skewness in the data and emphasise subtle conservation trends, a square root transformation was applied to the matrix. Heat maps of the PSFMs were visualized using matplotlib, with conserved positions annotated and residue indices labelled for interpretation. Detailed code has been provided^128^.

### Phylogenetic and motif analysis

We conducted a comprehensive phylogenetic analysis to investigate the evolutionary history of proteins containing actin-filament-binding motifs and their isoforms. Orthologous sequences were retrieved from OrthoDB^129^ and aligned using DECIPHER^130^ (100 iterations, 200 refinements). Maximum likelihood phylogenetic trees were constructed using IQ-TREE 2 (WAG + G model; 1,000 ultrafast bootstraps)^131^. Isoform sequences were identified and filtered based on header annotations and their phylogenetic clustering was visualized. Taxonomic information was incorporated to determine the MRCA of isoform clusters. Actin filament-binding motifs were extracted using regular expressions and filtered based on predicted disorder (IUPRED)^43^, anchor regions (ANCHOR)^42^ and random coil propensity (PSIPRED)^41^. Position-specific scoring matrices were generated and iteratively used to refine motif identification. The MRCA of motif-containing sequences was determined using isoform-specific trees. Motif distribution was validated by mapping motifs to a taxon-based tree. Motif development across isoforms and taxonomic classes was analysed using frequency matrices. Sequence logos^110^ were generated from PSSMs. For nucleotide-level analyses, the corresponding DNA sequences were retrieved from NCBI, filtered for ambiguous bases and stop codons, and codon-aligned using PRANK^132^. Phylogenetic trees were inferred with IQ-TREE 2 (GTR + G model)^131^, and dN/dS analysis was performed using HyPhy^133^. Detailed code and parameter settings are provided (Source data).

### Molecular dynamics simulation and actin-filament persistence length calculation

The MD simulations were performed with GROMACS2022.4 (ref. ^134–137^) using the CHARMM36m protein force field^138,139^. Starting coordinates were derived from the corresponding cryo-EM structures to ensure the most-accurate representation of each peptide–protein interface. Therefore, the asymmetric unit of the fitted cryo-EM models was expanded into a 13-subunit filament using a filament screw transformation in UCSF ChimeraX^140^. Specifically, the actin–ITPKA and actin–USP54 M1 systems were initiated from their corresponding experimental reconstructions. To provide a rigorous baseline, the actin-only control was generated by computationally removing the peptide from the ITPKA-bound scaffold. Although the initial coordinates for each SFM-bound system reflect their specific experimental binding modes, the subsequent all-atom MD simulations at 310 K allow all systems to explore thermally accessible conformations.

All expanded actin filaments were simulated in an ADP:Mg^2+^ bound state using the same protocol. Briefly, actin filaments were solvated in a cubic box with periodic boundary conditions and an initial minimum distance of 2 nm to all boundaries. All systems were charge-neutralized by the addition of potassium ions; a total KCl concentration of 0.18 M was used to approximate cytosolic salt concentrations. To broadly relax the systems, steepest-descent energy minimization was conducted, resulting in convergence to machine precision within 4,000–5,000 steps. Equilibration of temperature and pressure was achieved by two consecutive 100-ps equilibration MD runs. Temperature equilibration was performed in the NVT ensemble with stochastic velocity rescaling using the V-rescale thermostat^141^ at 310 K and a time constant of 0.1 ps. Separate temperature coupling groups were applied for the solute and the solvent. Subsequently, pressure coupling was achieved in the NPT ensemble by isotropic scaling of box vectors with the C-rescale barostat^142^ at 1 bar and a time constant of 2.0 ps (compressibility of 4.5 × 10^−5^ bar^−1^). All equilibration runs involved a position restraint potential with a force constant of 1,000 kJ mol^−1^ nm^−2^ for solute atoms including actin, peptide chains, ADP and Mg^2+^ ions.

Molecular dynamics production runs were conducted with the leap-frog integrator using a time step of 2 fs and coordinates were saved every 25 ps. While the same temperature coupling scheme as applied in the equilibration runs was used, isotropic pressure coupling was performed with the Parrinello–Rahman barostat^143^ at 1 bar with a time constant of 2 ps (compressibility of 4.5 × 10^−5^ bar^−1^). Hydrogen bond constraints were implemented with the Linear Constraint Solver using an order of four and one iteration^144^. Non-bonded interactions were treated with the Verlet cutoff scheme with grid-based neighbour searching. Neighbour lists were updated every 20 steps (40 fs) using a cutoff of 12 Å. Short-range van der Waals interactions were truncated at 12 Å by force-switching between 10 and 12 Å. Long-range electrostatics were calculated using the particle mesh Ewald method^145,146^ with a cutoff of 12 Å as well as fourth-order interpolation and 1.6 Å grid spacing for the fast Fourier transform grid. No dispersion correction was used with the CHARMM36m protein force field.

All trajectory analyses were performed using MDAnalysis^147,148^ in Python. Before analysis, MD trajectories were pre-processed with GROMACS tools to fix broken molecules and remove periodic boundary conditions as well as rotation and translation. For structural analyses (RMSD, RMSF and contacts), water and potassium ions were removed. To ensure analysis of equilibrated systems, the first 4 ns of each trajectory were discarded and subsequent analyses were performed on the 4–40 ns time window.

To calculate the *L*_p_ of actin filaments, pre-processed trajectories were read with MDAnalysis and a filament centreline was built by computing actin monomer centreline points based on the per-frame centre of mass (COM) of all atoms for each actin monomer^149^.

$${{\boldsymbol{r}}}_{{\boldsymbol{i}}}=0.5{{\rm{COM}}}_{i}+0.25\,\left({{\rm{COM}}}_{i-1}+{{\rm{COM}}}_{i+1}\right)$$

Discrete tangents are then computed from centerline point differences:

$${\tau }_{i}=\frac{{r}_{i+1}-{r}_{i-1}}{{||}{r}_{i+1}-{r}_{i-1}{||}}$$

Removal of spontaneous curvature was achieved by rotating ${{\boldsymbol{\tau }}}_{{\boldsymbol{i}}}$ such that the frame-averaged tangent aligns with the *z* axis. Finally, tangent–tangent correlations were computed as:

$$\left\langle \cos \theta \left(s\right)\right\rangle =\left\langle {{\boldsymbol{\tau }}}_{{\boldsymbol{i}}}\bullet {{\boldsymbol{\tau }}}_{{\boldsymbol{i}}{\boldsymbol{+}}{\boldsymbol{k}}}\right\rangle$$

By fitting the first three monotonically decreasing points of $\mathrm{ln}\left\langle \cos \theta \left(s\right)\right\rangle$ versus $s$ (with $s=k\delta s$), the *L*_p_ can be determined:

$${\rm{ln}}\langle \cos \theta (s)\rangle \approx -\frac{s}{{L}_{{\rm{p}}}}$$

The global RMSD was computed for Cα atoms relative to the initial frame using the MDAnalysis RMSD module. To avoid edge effects from filament termini, only interior actin chains (chains 2–10 of the 13-mer, chain index starting from zero) were included in the analysis. Per-chain RMSD trajectories were computed independently and aggregated to yield mean ± s.d. values across the filament. The RMSF of each residue was calculated using all heavy atoms excluding solvent and potassium ions with MDAnalysis. For each interior chain, per-residue RMSF values were computed after alignment to the mean structure. The values were then averaged across chains to generate mean ± s.d. profiles along the actin sequence (residues 1–375). Differential RMSF (ΔRMSF) was calculated by subtracting the actin-alone RMSF profile from each SFM-bound condition.

Atomic contacts between SFM peptides (ITPKA or USP54 M1) and actin were quantified using a distance cutoff of 4.0 Å applied to sidechain heavy atoms. For each SFM peptide chain (11 of 13 copies per filament considered), contacts with neighbouring actin subunits were evaluated at each trajectory frame. A residue–residue contact was defined as present when any heavy-atom pair fell within the cutoff distance. Contact frequency matrices were computed as the fraction of frames (normalized to the total number of frames × the number of SFM chains in contact) in which each SFM–actin residue pair was in contact. Time-resolved contact occupancy for individual SFM residues was calculated as the fraction of SFM peptides in contact with any actin residue at each time point. Actin contact occupancy profiles were computed as the fraction of trajectory frames in which each actin residue contacted at least one atom from any SFM peptide, aggregated across all SFM chains.

To monitor actin inter-subunit contacts, specific residue pairs were tracked over the trajectory. For inter-chain contacts (chain N with chain N−2), sidechain heavy atoms were selected and contacts were defined using a distance cutoff of 4.0 Å. Binary contact occurrence (present/absent) was recorded for each chain pair in each frame and the results were aggregated across all valid chain pairs in the interior of the filament. Time-resolved contact density was computed by binning the trajectory into 200-ps windows and calculating the fraction of chain pairs exhibiting the contact within each bin.

To visualize D-loop conformational heterogeneity, trajectory snapshots were extracted at regular intervals (first 4 ns discarded, snapshot every 1.8 ns from the remaining 36 ns) and superimposed. The D-loop coordinates (residues 35–55) from all frames were then overlaid to generate conformational ensembles for each condition. Representative structures were rendered in PyMOL^149^ to qualitatively assess the extent of conformational sampling in each system.

To analyse time-dependent conformational fluctuations of filament-internal actin protomers, the dihedral angle defined by the actin subdomains SD2-SD1-SD3-SD4 was computed as follows. The actin subdomain definition was adapted from Iyer and colleagues^150^: SD1 residues 1–32, 70–144, 338–375; SD2 residues 33–69; SD3 residues 145–180 and 270–337; SD4 residues 181–269. Due to previously described edge effects in protomers located at the pointed or barbed end of the actin filament, the two terminal actin protomers located at each of the filament ends were excluded from the analysis (13-mer filament with nine internal actin protomers). For each internal protomer, the COM of Cα atoms belonging to each subdomain was calculated. The dihedral angle was then determined from the four COM points (SD2-SD1-SD3-SD4) for every trajectory frame. To obtain a filament-averaged dihedral angle for each frame, the individual dihedral angles of all nine internal actin protomers were averaged. For visualization of time-dependent dihedral angle changes, circular running means were calculated with a window size of 2 ns.

### Gene ontology enrichment analysis

To elucidate the biological functions and cellular components associated with SFM-containing proteins, we performed GO enrichment analysis using ShinyGO^151^. The analysis aimed to identify over-represented GO terms within three primary categories: biological process, cellular component and molecular function. We compiled a comprehensive list of SFM-containing proteins identified in this study as the query set. Utilizing ShinyGO with its standard parameters, we input the query set to perform the enrichment analysis. The analysis employed the default settings for multiple-testing correction, including the false-discovery rate, ensuring that only GO terms with an adjusted *P* value of <0.05 were considered significantly enriched.

### AlphaMissense

For each of the 12 proteins examined in this study, the AlphaMissense pathogenicity score^152^ was retrieved using the canonical UniProt ID. The relevant amino acid positions were then manually aligned based on the SFM coordinates. Residues flanking the motif (positions −30 to −20 and +30 to +40) served as control values for comparative analysis. After extracting the AlphaMissense scores for all 12 proteins, the mean score at each aligned position was calculated and the s.e.m. determined. Statistical evaluation of differences between motif residues and the flanking control positions was performed via one-way analysis of variance with multiple comparisons.

### Phosphorylation prediction

All SFM-containing protein sequences used in this study were analysed with NetPhos 3.1 (ref. ^153^). For each sequence position containing Ser or Thr, a site-level maximum score across all NetPhos kinase models was computed. Motif-relative positional groups were defined a priori as N-edge (P−2 to P0), C-edge (P10 to P12), in-ring interacting (P1, P4, P5, P8 and P9), in-ring non-interacting (P2, P3, P6 and P7) and outside (all remaining positions). For each group, unique sequence, position (S/T) sites and the subset with high-confidence predictions (score ≥ 0.90) were counted, with outside serving as background. Enrichment of high-confidence sites relative to outside was quantified as odds ratios with Wald 95% confidence intervals; when necessary, a Haldane–Anscombe correction (0.5 added to each cell) was applied. Significance was assessed using two-sided Fisher’s exact tests. Results were visualized as bar charts of high-confidence fractions, forest plots of odds ratio ± 95% confidence intervals and kinase-resolved position plots.

### Statistics and reproducibility

The Poji macro is a semi-automated ImageJ/Fiji plugin created to characterize protein distribution and enrichment at podosomes. For good detection quality, the ‘prominence’ value (signal-to-noise ratio) was set at 100 and podosomal circular ROI detection size at 25 pixels per podosome among all analysed samples. During analysis, the fluorescence intensity of 360° profiles of single podosomal ROIs was measured. Optical *z*planes of highest F-actin intensity with mean-normalized ± s.d. values of F-actin and the respective SFM peptide are shown. Correlation analysis was performed using GraphPad Prism 10.2.3 (GraphPad software). For each analysed peptide, 180–953 podosomes, in 3 cells (*n* = 3) were characterized. No statistical method was used to pre-determine sample size. No data were excluded from the analysis. The experiments were not randomized. The investigators were blinded to allocation during experiments and outcome assessment. Plasmids encoding different peptides were de-identified using single-digit numbers.

For the determination of *K*_d__,app_values, co-sedimentation assays were performed in triplicate. The investigators were blinded to allocation during experiments and outcome assessment, and samples were randomly assigned to experimental groups. Co-sedimentation with actin filaments was analysed by western blotting and band intensities were quantified using ImageJ. Signals were normalized to the peptide signal in the absence of actin filaments, and normalized band intensities were plotted against the actin-filament concentration. *K*_d,app_ values were determined in GraphPad Prism version 8.0.2 using nonlinear regression with a one-site specific binding model. Replicate-wise *K*_d__,app_ values were obtained from three independent experiments and global-fit *K*_d__,app_ values were obtained by fitting all replicate measurements together. For comparison of apparent affinities, binding signals in each experiment were normalized to the plateau of the respective binding curve before nonlinear regression analysis. The *K*_d__,app_ values were then compared using an extra-sum-of-squares *F*-test. Because this normalization removes differences in maximal signal amplitude, statistical analysis was restricted to apparent *K*_d__,app_ differences and did not assess differences in maximum binding capacity (B_max_).

For analyses of quantified relative band intensities normalized to the WT, statistical significance was assessed using a one-way analysis of variance with Dunnett’s multiple comparisons test. Data were excluded only in cases of technical failure. The number of replicates (*n* = 3) represents the minimal sample size for statistical testing. No statistical method was used to pre-determine sample size. Data met the assumptions of the statistical tests and normal distribution was assumed but not formally tested.

For evaluating significant differences in the occurrence of phosphorylation sites, a two-sided Fisher’s exact tests on 2 × 2 contingency tables of high-confidence versus not high-confidence NetPhos serine/threonine predictions was used.

### Reporting summary

Further information on research design is available in the Nature Portfolio Reporting Summary linked to this article.

## Online content

Any methods, additional references, Nature Portfolio reporting summaries, source data, extended data, supplementary information, acknowledgements, peer review information; details of author contributions and competing interests; and statements of data and code availability are available at 10.1038/s41556-026-01979-9.

## Supplementary information

Reporting Summary

Peer Review File

Supplementary Tables 1–6 Table 1. Representative examples of identified ABDs assigned into SLiMs, intrinsically disordered domains and globular domains. Table 2. Cryo-electron microscopy data collection, model refinement and validation statistics. Table 3. Glossary of predicted SFM proteins. Table 4. Plasmid list. Table 5. Primer list

Supplementary Video 1 This video illustrates how the short linear F-actin-binding motif docks inside the hydrophobic binding groove of the filament.

## Source data

Source Data all Master source data workbook organized on a figure-by-figure basis, with one worksheet per figure as applicable.

Source Data Fig. 3 Uncropped western blots. Band intensities were analysed to calculate apparent dissociation constants in Fig. 3b.

## Data Availability

Coordinates and cryo-EM maps for the actin filament structure have been deposited in the Electron Microscopy Data Bank (EMDB) under the accession code EMD-18866, with corresponding Protein Data Bank (PDB) entry 8R3H. The actin filament–ITPKA complex has been deposited in the EMDB under accession code EMD-53133 and in the PDB under accession code 9QGK. The actin filament in complex with the USP54 M1 mutant has been deposited in the EMDB under accession code EMD-54871 and in the PDB under accession code 9SGK. In addition, the previously published PDB entries 6T20 (ref. ^122^), 7BTI ref. ^40^ and 7AD9 (ref. ^9^) were used for structural comparisons. Source data for this study are avaiable at *Zenodo* (10.5281/zenodo.19039418; 10.5281/zenodo.19017320; 10.5281/zenodo.19017329 and 10.5281/zenodo.19017361)^154–157^. Numerical and uncropped western blot Source data are provided. All other data supporting the findings of this study are available from the corresponding author on reasonable request.

## Code availability

The SLiMFold pipeline code is available at https://github.com/thp42/slimfold and *Zenodo* (10.5281/zenodo.19039117)^158^. The PSFM code is provided at https://github.com/thp42/psfm and *Zenodo* (10.5281/zenodo.10702806)^159^. The phylogenicity code is available at https://github.com/thp42/phylogenicity.
