# Supplementary material for: Evolutionarily conserved short linear motifs drive actin filament binding
Source: Nat Cell Biol. 2026 Jul 6;28(7):1437–52. doi: 10.1038/s41556-026-01979-9 (PMC13364712; doi:10.1038/s41556-026-01979-9)
Supplement: Supplementary file 1 — Reporting Summary [file 41556_2026_1979_MOESM1_ESM.pdf]

nature portfolio

Corresponding author(s): Sabine Windhorst, NCB-A57869R3

Last updated by author(s): 20/03/26

Reporting Summary

Nature Portfolio wishes to improve the reproducibility of the work that we publish. This form provides structure for consistency and transparency in reporting. For further information on Nature Portfolio policies, see our Editorial Policies and the Editorial Policy Checklist.

Statistics

For all statistical analyses, confirm that the following items are present in the figure legend, table legend, main text, or Methods section.

n/a	Confirmed	
☐	☒	The exact sample size (*n*) for each experimental group/condition, given as a discrete number and unit of measurement
☐	☒	A statement on whether measurements were taken from distinct samples or whether the same sample was measured repeatedly
☐	☒	The statistical test(s) used AND whether they are one- or two-sided<br>*Only common tests should be described solely by name; describe more complex techniques in the Methods section.*
☐	☒	A description of all covariates tested
☐	☒	A description of any assumptions or corrections, such as tests of normality and adjustment for multiple comparisons
☐	☒	A full description of the statistical parameters including central tendency (e.g. means) or other basic estimates (e.g. regression coefficient) AND variation (e.g. standard deviation) or associated estimates of uncertainty (e.g. confidence intervals)
☐	☒	For null hypothesis testing, the test statistic (e.g. F, t, r) with confidence intervals, effect sizes, degrees of freedom and P value noted<br>*Give P values as exact values whenever suitable.*
☒	☐	For Bayesian analysis, information on the choice of priors and Markov chain Monte Carlo settings
☒	☐	For hierarchical and complex designs, identification of the appropriate level for tests and full reporting of outcomes
☒	☐	Estimates of effect sizes (e.g. Cohen's *d*, Pearson's *r*), indicating how they were calculated

Our web collection on statistics for biologists contains articles on many of the points above.

Software and code

Policy information about availability of computer code

Data collection

Fluorescence microscopy: FLUOVIEW version
Protein purification by IMAC and size-exclusion chromatography: Chromlab version 6.0.0.35
Western Blot image acquisition: ChemoStar Touch version 0.5.84

Data analysis

Cryo-EM data analysis and structure determination: RELION version 4.0

Fluorescence microscopy data analysis: Fiji version 1.54p with additional custom code for podosome analysis available here: https://github.com/roherzog/Poji

Western Blot images were analysed with Fiji version 1.54p.

Peptide binding affinity calculation and data visualization: GraphPad Prism version 8

The SLiMFold-code is publicly available at https://github.com/thp42/SLiMFold. It's dependencies are:
numpy v.1.26.2
pandas v.2.2.2
blosum v.2.0.3
scikit-learn v.1.5.2
matplotlib v.3.9.2
seaborn v.0.13.1
hmmer v.3.4

hhsuite v.3.3.0
biopython v.1.82
hdbscan v.0.8.40
ansible v.2.18.0

The PSFM-code is publicly available at https://github.com/thp42/PSFM. It's dependencies are:
numpy v.1.26.2
matplotlib v.3.9.2

The phylogenicity analysis code is publicly available at https://github.com/thp42/Phylogenicity. It's dependencies are:

For manuscripts utilizing custom algorithms or software that are central to the research but not yet described in published literature, software must be made available to editors and reviewers. We strongly encourage code deposition in a community repository (e.g. GitHub). See the Nature Portfolio guidelines for submitting code & software for further information.

Data

Policy information about availability of data

All manuscripts must include a data availability statement. This statement should provide the following information, where applicable:

- Accession codes, unique identifiers, or web links for publicly available datasets
- A description of any restrictions on data availability
- For clinical datasets or third party data, please ensure that the statement adheres to our policy

Coordinates and cryo-EM maps for the F-actin structure have been deposited in the Electron Microscopy Data Bank (EMDB) under accession code EMD-18866, with corresponding Protein Data Bank (PDB) entry 8R3H. The F-actin–ITPKA complex has been deposited in the EMDB under accession code EMD-18868 and in the PDB under accession code 8R3J. Additionally, PDB entries 6T20, 7BTI, and 7AD9 were used for structural comparisons. All in silico, in vitro and in cellulo source data generated in this study is made publicly available via Zenodo.

Research involving human participants, their data, or biological material

Policy information about studies with human participants or human data. See also policy information about sex, gender (identity/presentation), and sexual orientation and race, ethnicity and racism.

Reporting on sex and gender	N/a
Reporting on race, ethnicity, or other socially relevant groupings	N/a
Population characteristics	N/a
Recruitment	N/a
Ethics oversight	N/a

Note that full information on the approval of the study protocol must also be provided in the manuscript.

Field-specific reporting

Please select the one below that is the best fit for your research. If you are not sure, read the appropriate sections before making your selection.

☒ Life sciences ☐ Behavioural & social sciences ☐ Ecological, evolutionary & environmental sciences

For a reference copy of the document with all sections, see nature.com/documents/nr-reporting-summary-flat.pdf

Life sciences study design

All studies must disclose on these points even when the disclosure is negative.

Sample size	For determination of KD-values western blot data were analyzed by ImageJ. For this, three different western blots were performed and KD values were calculated by non-linear fitting of appropriate binding models.
Data exclusions	No experimental data were excluded from the analysis. From the SliMFold screen, hits with ipTM < 0.6 were excluded
Replication	The SLiMFold-pipeline used experimentally validated actin binding proteins as starting point and, among others, independently identified several previously validated and characterized actin binding proteins (detailed list provided in the manuscript). Three independent actin cosedimentation assays were performed for experimental validation of predicted actin binding proteins.
Randomization	No randomized sample selection was used.

Blinding	Blinding was not relevant for this study as all experiments were done with samples of known composition.

Reporting for specific materials, systems and methods

We require information from authors about some types of materials, experimental systems and methods used in many studies. Here, indicate whether each material, system or method listed is relevant to your study. If you are not sure if a list item applies to your research, read the appropriate section before selecting a response.

Materials & experimental systems

n/a	Involved in the study
☐	☒ Antibodies
☐	☒ Eukaryotic cell lines
☒	☐ Palaeontology and archaeology
☒	☐ Animals and other organisms
☒	☐ Clinical data
☒	☐ Dual use research of concern
☒	☐ Plants

Methods

n/a	Involved in the study
☒	☐ ChIP-seq
☒	☐ Flow cytometry
☒	☐ MRI-based neuroimaging

Antibodies

Antibodies used	GFP: mouse anti-GFP, Article No. 11814460001, Roche Applied Science, Penzberg, Germany
Validation	commercially available

Eukaryotic cell lines

Policy information about cell lines and Sex and Gender in Research

Cell line source(s)	NCL-H1299 were a gift from Cagatay Gunez, for detailed cellular characteristics, refer to the American Type Culture Collection (ATCC-CRL-5803 , Rockville, USA). Human primary macrophages were prepared from buffy coats.
Authentication	Cell line authentication was performed by the Leibniz Institute DSMZ–German Collection of Microorganisms and Cell Cultures (28 January 2025) using analysis of 17 short tandem repeat (STR) loci.
Mycoplasma contamination	is routinely tested by a PCR based assay
Commonly misidentified lines (See ICLAC register)	No commonly misidentified lines per ICLAC register were used

Plants

Seed stocks	N/a
Novel plant genotypes	N/a
Authentication	N/a
