# Supplementary material for: Evolutionarily conserved short linear motifs drive actin filament binding
Source: Nat Cell Biol. 2026 Jul 6;28(7):1437–52. doi: 10.1038/s41556-026-01979-9 (PMC13364712; doi:10.1038/s41556-026-01979-9)

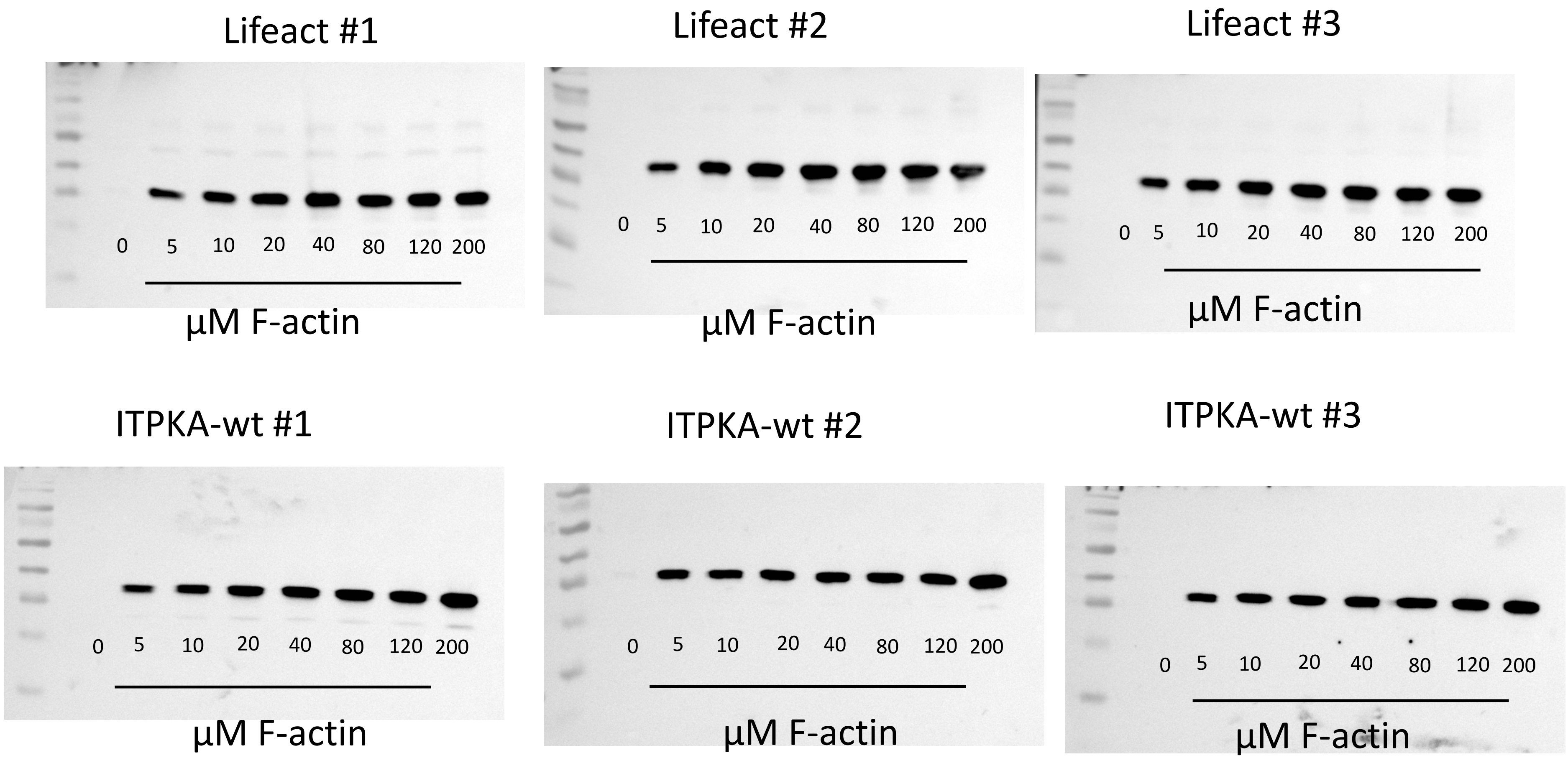

Lifeact #1
0 5 10 20 40 80 120 200
µM F-actin
Lifeact #2
0 5 10 20 40 80 120 200
µM F-actin
Lifeact #3
0 5 10 20 40 80 120 200
µM F-actin
ITPKA-wt #1
0 5 10 20 40 80 120 200
µM F-actin
ITPKA-wt #2
0 5 10 20 40 80 120 200
µM F-actin
ITPKA-wt #3
0 5 10 20 40 80 120 200
µM F-actin

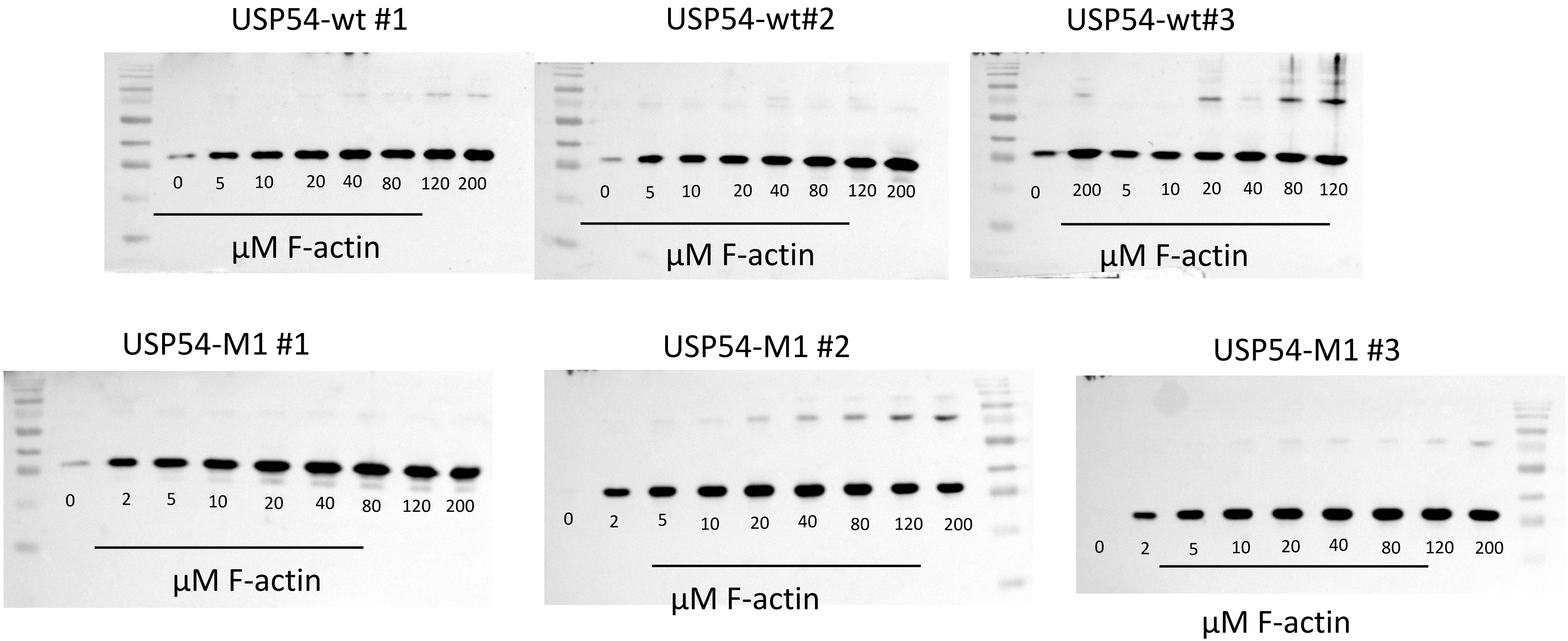
USP54-wt #1
0 5 10 20 40 80 120 200
μM F-actin
USP54-wt#2
0 5 10 20 40 80 120 200
μM F-actin
USP54-wt#3
0 200 5 10 20 40 80 120
μM F-actin
USP54-M1 #1
0 2 5 10 20 40 80 120 200
μM F-actin
USP54-M1 #2
0 2 5 10 20 40 80 120 200
μM F-actin
USP54-M1 #3
0 2 5 10 20 40 80 120 200
μM F-actin

SHROOM #1

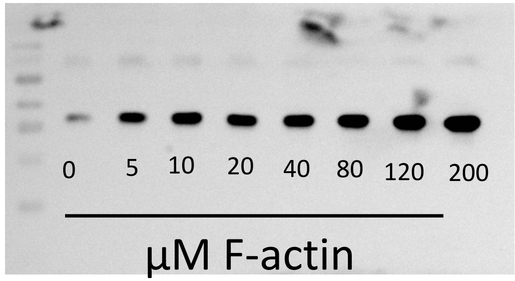

SHROOM #2

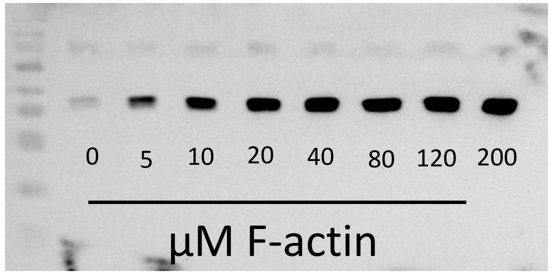

SHROOM #3

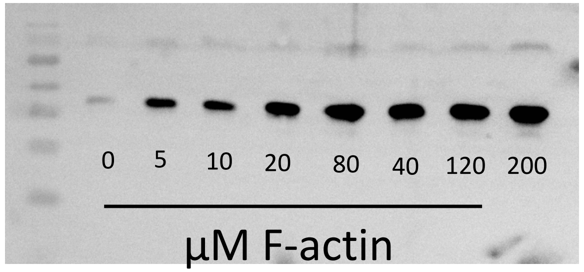

SHROOM-M1 #1

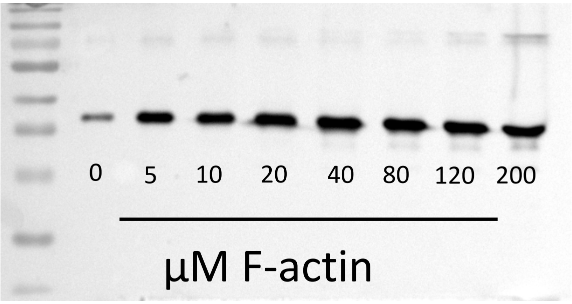

SHROOM-M1 #2

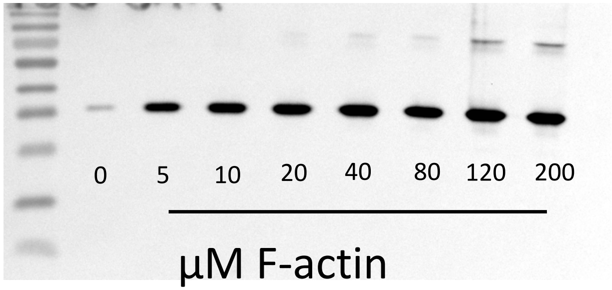

SHROOM-M1 #3

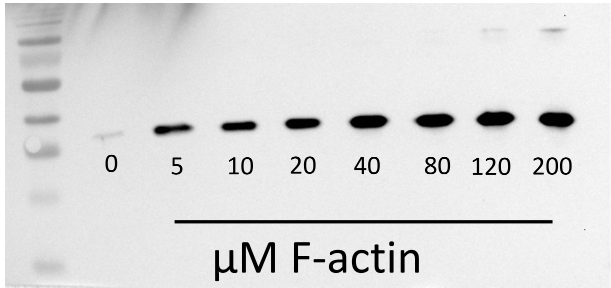

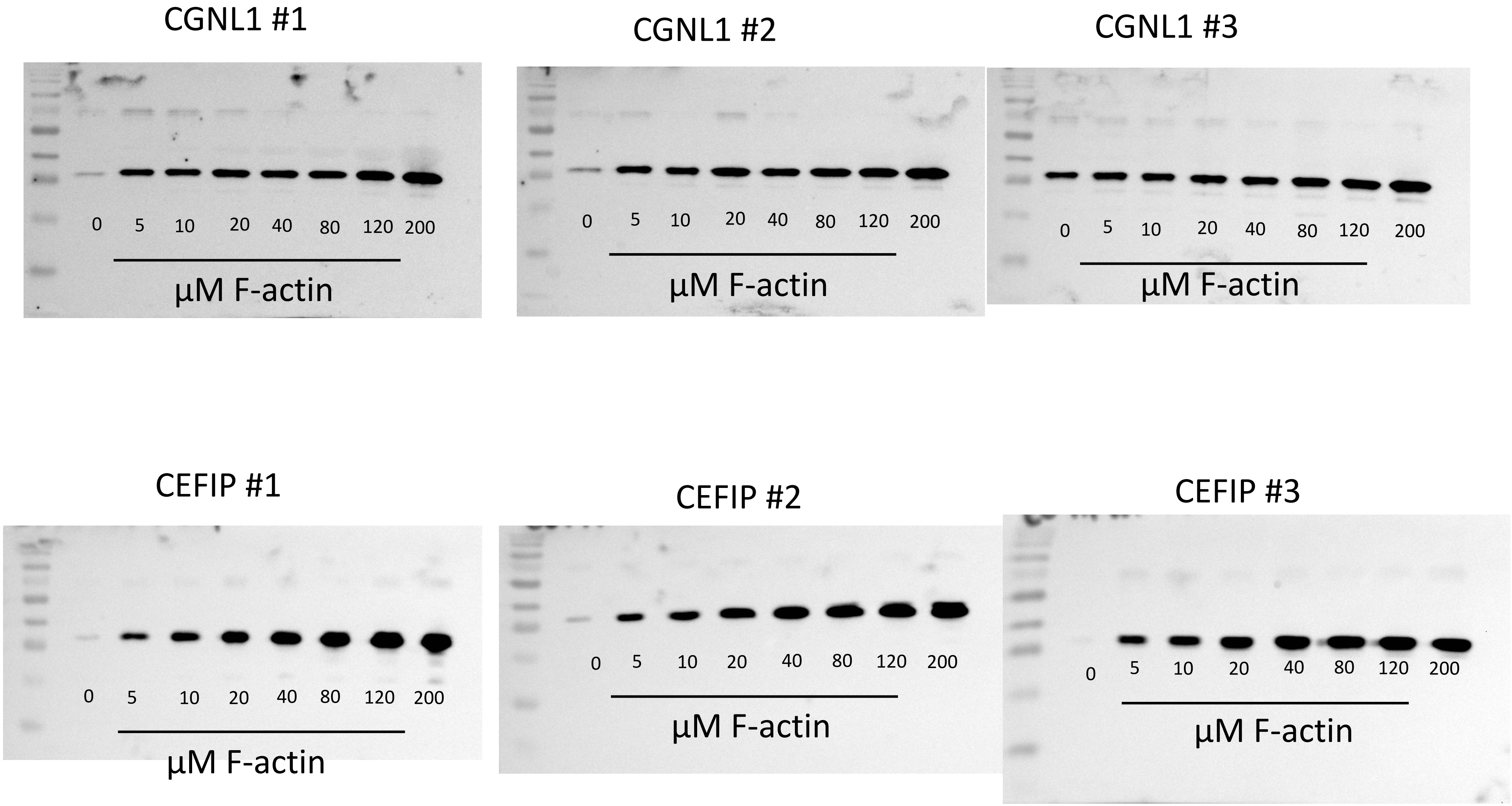
CGNL1 #1
0 5 10 20 40 80 120 200
µM F-actin
CGNL1 #2
0 5 10 20 40 80 120 200
µM F-actin
CGNL1 #3
0 5 10 20 40 80 120 200
µM F-actin
CEFIP #1
0 5 10 20 40 80 120 200
µM F-actin
CEFIP #2
0 5 10 20 40 80 120 200
µM F-actin
CEFIP #3
0 5 10 20 40 80 120 200
µM F-actin

ESPNL1 #1

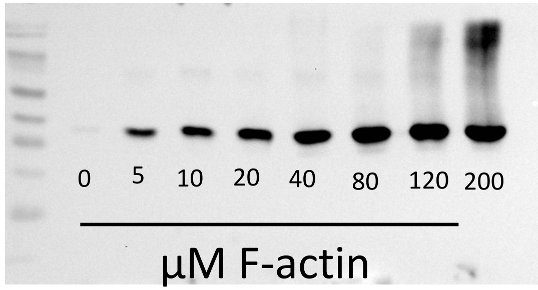

ESPNL1 #2

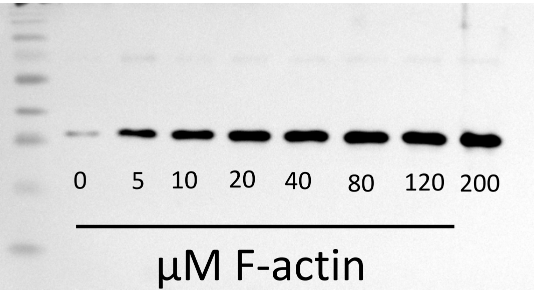

ESPNL1 #3

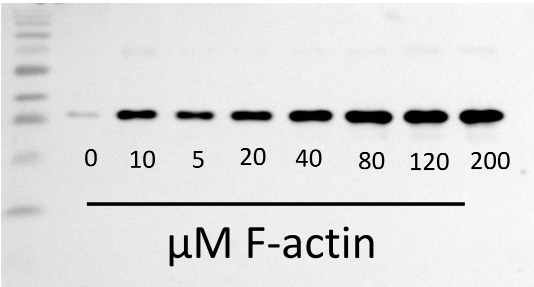

Supplement: Supplementary file 6 — Uncropped western blots. Band intensities were analysed to calculate apparent dissociation constants in Fig. 3b. [file 41556_2026_1979_MOESM6_ESM.pdf]
