# Supplementary material for: Evolutionarily conserved short linear motifs drive actin filament binding
Source: Nat Cell Biol. 2026 Jul 6;28(7):1437–52. doi: 10.1038/s41556-026-01979-9 (PMC13364712; doi:10.1038/s41556-026-01979-9)
Supplement: Supplementary file 2 — Peer Review File [file 41556_2026_1979_MOESM2_ESM.pdf]

nature portfolio

Peer Review File

Evolutionarily conserved short linear motifs drive actin filament binding

Corresponding Author: Professor Sabine Windhorst

Version 0:

Decision Letter:

Revise extended OD

*Please delete the link to your author homepage if you wish to forward this email to co-authors.

Dear Professor Windhorst,

I am writing on behalf of my colleague, Dr. Daryl David, who is out of the office.

Your manuscript, "Discovery of a new evolutionarily conserved short linear F-actin binding motif", has now been seen by 3 referees, who are experts in cryoEM, mechanobiology (referee 1); protein self-assembly, computational methods (referee 2); and actin, structure (referee 3), with our most sincere apologies for the delayed decision. As you will see from their comments (attached below) they find this work of potential interest, but have raised substantial concerns, which in our view would need to be addressed with considerable revisions before we can consider publication in Nature Cell Biology.

Nature Cell Biology editors discuss the referee reports in detail within the editorial team, including the chief editor, to identify key referee points that should be addressed with priority, and requests that are overruled as being beyond the scope of the current study. To guide the scope of the revisions, I have listed these points below. We are committed to providing a fair and constructive peer-review process, so please feel free to contact me if you would like to discuss any of the referee comments further.

In particular, it would be essential to:

) Carry out additional structure/function studies to address the questions of all 3 reviewers about the structure of SFM peptides bound to actin filaments.
B) Clarify evolutionary analysis (reviewer 1)

C) All other referee concerns pertaining to strengthening existing data, providing controls, methodological details, presentation of data including cryo-EM data, clarifications and textual changes, should also be addressed.

D) Finally please pay close attention to our guidelines on statistical and methodological reporting (listed below) as failure to do so may delay the reconsideration of the revised manuscript. In particular please provide:

- a Supplementary Figure including unprocessed images of all gels/blots in the form of a multi-page pdf file. Please ensure that blots/gels are labeled and the sections presented in the figures are clearly indicated.

- a Supplementary Table including all numerical source data in Excel format, with data for different figures provided as different sheets within a single Excel file. The file should include source data giving rise to graphical representations and statistical descriptions in the paper and for all instances where the figures present representative experiments of multiple independent repeats, the source data of all repeats should be provided.

We would be happy to consider a revised manuscript that would satisfactorily address these points, unless a similar paper is published elsewhere, or is accepted for publication in Nature Cell Biology in the meantime.

When revising the manuscript please:

- ensure that it conforms to our format instructions and publication policies (see below and www.nature.com/nature/authors/).

- provide a point-by-point rebuttal to the full referee reports verbatim, as provided at the end of this letter.

- provide the completed Editorial Policy Checklist (found here https://www.nature.com/authors/policies/Policy.pdf), and Reporting Summary (found here <a

href="https://www.nature.com/authors/policies/ReportingSummary.pdf">https://www.nature.com/authors/policies/ReportingSummary.pdf). This is essential for reconsideration of the manuscript and these documents will be available to editors and referees in the event of peer review. For more information see http://www.nature.com/authors/policies/availability.html or contact me.

Nature Cell Biology is committed to improving transparency in authorship. As part of our efforts in this direction, we are now requesting that all authors identified as 'corresponding author' on published papers create and link their Open Researcher and Contributor Identifier (ORCID) with their account on the Manuscript Tracking System (MTS), prior to acceptance. ORCID helps the scientific community achieve unambiguous attribution of all scholarly contributions. You can create and link your ORCID from the home page of the MTS by clicking on 'Modify my Springer Nature account'. For more information please visit please visit www.springernature.com/orcid.

Please submit the revised manuscript files and the point-by-point rebuttal to the referee comments using this link:

Link Redacted

*This url links to your confidential home page and associated information about manuscripts you may have submitted or be reviewing for us. If you wish to forward this email to co-authors, please delete the link to your homepage.

We would like to receive a revised submission within six months. We would be happy to consider a revision even after this timeframe, however if the resubmission deadline is missed and the paper is eventually published, the submission date will be the date when the revised manuscript was received.

We hope that you will find our referees' comments, and editorial guidance helpful. Please do not hesitate to contact me if there is anything you would like to discuss.

With best wishes,

Christina

Christina Kary, PhD
Chief Editor
Nature Cell Biology
1 New York Plaza

Reviewers' Comments:

Reviewer #1 (Remarks to the Author):

In their paper "Discovery of a new evolutionarily conserved short linear F-actin binding motif", Paraschiakos and colleagues present evidence for a widely distributed conserved "Short Linear Motif" (SLiM) peptide sequence that confers actin-binding activity on many proteins, which they name a "short linear F-actin binding motif" (SFM). Using cryo-EM structural analysis, they resolve the SFM of full-length ITPKA bound to F-actin, confirming a previously reported structure of this peptide bound to F-actin (which corresponds to the widely-used F-actin reporter F-tractin). Guided by this result and the similar structure of Lifeact, the authors proceed to develop (what appears to this non-expert) a sophisticated bioinformatics and protein structure prediction-based pipeline to identify more proteins containing predicted SFMs in many species. Impressively, the authors then validate many of these predictions experimentally, with a mixture of cellular F-actin colocalization assays and biochemical F-actin binding assays. They furthermore develop a bioinformatic model of the sequence determinants of SFM affinity, allowing them to produce SFMs with improved actin-binding affinity through mutagenesis. They next perform evolutionary analysis which they claim supports the convergent evolution "ex-nihilo" of SFMs in many proteins, which have become fixed through purifying selection. Finally, they expand their bioinformatic analysis to identify over 100 putative SFMs in the human genome, which are found in proteins of diverse functions. Many of these SFMs have not been experimentally characterized, providing a resource that may guide future studies into the role of F-actin binding activity in many unexpected pathways.

Overall, this is largely a straightforward and conceptually appealing paper. With the exception of the evolutionary analysis (which we found a bit confusing, detailed below), the data from multiple experimental and computational threads soundly cohere to support the authors' conclusions. The identification of SFMs is likely to stimulate substantial future cell biology research. Furthermore, the authors' bioinformatic model of the SFM has the potential to enable the development of improved or fine-tuned F-actin imaging probes (similar to Lifeact or F-tractin), an idea that was not mentioned in the text. We are thus very enthusiastic about the likely impact of this paper, and we believe it has the potential to be suitable for publication in Nature Cell Biology, pending revision to address the modest concerns outlined below:

Scientific issues:

1) The presentation of the evolutionary analysis is confusing, particularly for a non-specialist reader. Why does a particular dN/dt value indicate purifying selection? How precisely did the authors come to the conclusion that the SFM motifs arose ex-nihilo? This part is particularly problematic, as there appear to be inconsistencies in the presentation. E.g. the authors write "To examine the evolutionary conservation of the SFM motif, we generated sequence logos for 12 SFM-containing proteins… These seven classes were chosen because…" (lines 205-208). However, in Figure 4a sequences logos from 9 "representative organism classes" are shown. It is unclear whether these correspond to 12 proteins across these 9 (not 7) classes, or what precisely is being depicted. This is also true for the tree shown in Figure 4c. This should be clarified.

2) In addition to facilitating the evolution of SLiMs, unstructured regions of proteins are often enriched for posttranslational modifications (PTMs). Are SFMs enriched in known or potential PTM sites? For instance, 7 out of the 8 SFMs displayed in Figure 3a contain serine

and/or threonine residues, either inside the SFM or within 1 residue from the P1 / P9 boundaries, suggesting the potential for phosphorylation.

3) The cryo-EM analysis appears to be sound and well-executed. However, there are some minor issues with the cryo-EM data presentation. Specifically, the authors should show an image of the atomic model fit into the experimental cryo-EM density map, particularly highlighting the fit of the SFM. They should also, at a minimum, display half-map and map-model Fourier Shell Correlation (FSC) curves. Additionally, the EMD / PDB entries for the ITPKA-F-actin complex corresponding to the accession codes provided in the data availability statement appear to have been withdrawn. However, it seems like those in the provided validation report / Table S4 are still active (HPUB). So, this should be updated.

4) Several canonical F-actin binding domains with folded cores feature extended unstructured "tails" which engage the F-actin surface in a linear fashion upon binding. This includes cell adhesion proteins (e.g. vinculin and alpha-catenin) and proteins containing calponin-homology domains (CHDs), e.g. calponin, filamin, spectrin, etc. Many of these domains have been structurally resolved bound to F-actin. The Introduction and / or Discussion could be expanded to mention this as a common theme. It could also be interesting to compare / contrast the binding modes of these "tails" with the results presented here.

Presentation / Clarification:

5) The presentation of the predicted SFM /F-actin complexes in Figure 1 could enhanced by including a more directly visual depiction of the results. E.g., a Ca superposition of all of the complexes assigned as SFMs, versus those that were rejected (which might be shown in a supplemental figure).

6) It would be better to show two-color fluorescence images as magenta / green rather than red / green for colorblind readers.

7) In the Methods, the "Poji" analysis would benefit from a brief explanation, rather than simply referring to a prior publication (line 592).

8) Lines 610 – 616, the authors refer to selecting a binding model based on the amount of remaining "free actin", but likely they mean "free ABP".

9) Lines 299-301, it would be clearer to say that SFMs "Do not need to be clustered in repeats to be necessary for F-actin targeting".

10) Line 192, "Similary USP54 mutants…", this sentence is unfinished.

11) Line 186, "Figure 4" references should likely be "Figure 3".

12) Title and legend of Extended Data Fig. 7, "pulldown assay" should probably be "co-sedimentation assay". It would be useful to include the F-actin concentrations in this figure legend, and the SHROOM protein concentration in b.

13) A matter of personal preference, but the authors frequently use phrases such as "a lot of" and "A high number of", this could be simplified to "Many".

14) In general, the paper would benefit (as is generally the case), from careful proofreading and ensuring that phrases have their intended meaning. As one example, line 316, "it will be very exciting to elucidate their role in actin targeting…", likely the thing of interest is what functional role actin targeting plays in these proteins, rather than vice-versa.

Reviewer #1 (Remarks on code availability):

I have not performed a detailed review of the code, as I do not have the relevant bioinformatics expertise. However, I can confirm that it is available on GitHub as described, and that the code has documentation provided which is comprehensible.

Reviewer #2 (Remarks to the Author):

Review: Discovery of a new evolutionarily conserved short linear F-actin binding motif.

In the article "Discovery of new evolutionarily conserved short linear F-actin binding motif" the authors report a new cryo-EM determined structure of human Inositoltriphosphate 3-kinase A (ITPKA)-F-actin complex. They perform a bioinformatics analysis of amino acid sequence in actin binding proteins and identify a new VxxLxxxFE motif present in short linear F-actin binding helical peptides.

However, the authors should address the following questions/concerns regarding the results on association of SFM-peptides to F-actin. These may require significant revision.

1) Kd values in μM range reported for Group 2 candidates indicate weak and nonspecific interactions which could also be due to the non-specific interactions of GFP.
How is this ruled out?

2) Can the authors report the difference in Kd values of the P1/P8 mutants?

3) Difference in Kd values for the WT and M1 (triple mutant) correspond to about 1 kcal/mol difference in free energy of binding. This is within a thermal fluctuation (kBT is 0.6 kcal/mol). How is this significant?

4) The authors should also study F-actin association with simultaneous mutations of P1/P8 and residues in F-actin that interact with P1/P8?

5) D-loop interactions are known to stiffen the actin filament (see, Chu and Voth, www.pnas.org/cgi/doi/10.1073/pnas.0503732102; Kang H et al. Proc Natl Acad Sci U S A. 2012 , doi: 10.1073/pnas.1211078109; Hocky GM et al. J Phys Chem B. 2016 , doi: 10.1021/acs.jpcb.6b02741). Did ITPKA binding to the D-loop stiffen the actin filament?

6) On page 8, “mutant S2 and U2” are not defined.

7) The small range of helix orientations in Fig. 1h appears to be an outcome of steric clashes with actin subunits rather than the effect of SLiM sequence conservation. This should be clarified

8) In Group 1 SMF peptide studies, P1 is mutated from V to A , but these amino acids have very similar characteristics. The authors should study how V to D/E mutation change F-actin association?

Reviewer #3 (Remarks to the Author):

This work uses cryo-EM structures reported here and recently published by others of a peptide from the protein ITPKA also called “F-tractin” to search similar actin filament-binding motifs in other proteins. The authors find a remarkable number of proteins incorporating related peptides characterized by the residues Val, Leu, Phe and Glu in positions 1, 4, 8 and 9 (VxxLxxxFE). Samples of these peptides colocalized with phalloidin-stained actin filaments in cultured cells and bound to actin filaments in co-sedimentation assays. The evolutionary analysis is a strong point of the paper. These findings are interesting and likely to generate additional research of value.

On the other hand, the manuscript has weaknesses including incomplete or unclear explanations of the work, problems with the design and interpretation of some assays and missed opportunities to understand mechanisms. In particular, the authors emphasize the output of bioinformatic tools but miss many opportunities to understand the relation of structure to function, by not studying and thinking about the structures of the peptides bound to actin filaments. Additional work is required to correct these problems.

Detailed comments

The introduction emphasizes well characterized WH2 motifs but they are not compared structurally or functionally with the STM peptides.

Line 123 following: The informatic procedures for finding actin-binding peptides related to VxxLxxxFE need to be explained more fully. For example, the paragraph does not explain the creation of atomic models of peptides bound to actin filaments or explain the question of polarity.

Line 129: “detailed comparison against the ITPKAArg28-Ala49–actin complex.” Rather than or in addition to using this automated method, why not simply make homology models of each peptide bound to an actin filament based on the ITPKA structure? Identifying steric clashes would explain weak or no binding. Explain how “HDBSCAN” works. What is the meaning of “orientation shifts relative to ITPKA?” Do some peptides have the opposite polarity?

Line 140: “Validation of SFM-peptides in cellulo and in vitro”. In my opinion, these terms are misused. Since the invention of tissue culture a century ago, “in vitro” has been used to describe cultured cells, not biochemical and biophysical studies (also in glass) of purified proteins and other macromolecules. “In cellulo” is a vague, silly recent neologism. It does not reveal whether the cell is living or dead, in culture or in a multicellular organism. “In cells,” “in vitro” and “in vivo” are clearer.

Lines 148-149. Cite the relevant figures.

Line 159: “Together, these data indicate that the SFM binds to F-actin with different efficiency, and preference to F-actin subpopulations.” Efficiency is a thermodynamic parameter not relevant to this point. How can these peptides have preferences for different actin filament subpopulations? Do you have an hypothesis? The observation that the mutants (V→A and F→A) at positions 1 and 8 do not bind actin is obvious from the structures and the biochemical experiments. Were these mutants tested in cells to rule out nonspecific binding?

Line 168, following: Actin filament binding assays. Varying the actin filament concentration is exactly what to do. However, it is impossible to measure a Kd unless the binding curves reach saturation, which accomplished for only two peptides (CEFIP and USP54) in Fig. 3B and two USP54 peptides in Fig. 3F but not the other peptides tested. If P9E is so important for binding, why does USP54 with P9Q have a higher affinity for actin filaments than other peptides with P9E? Using either SD or SEM to describe the variation of three data points is inappropriate, since one cannot verify the assumption of normal distributions with three points; plot the three data points and the mean instead. What is the actin filament concentration used in Fig. 3E?

Line 172: I would look at atomic models for steric interference or other problems to explain the wide range of apparent affinities for actin filaments in Fig. 4B.

Line 184, following and Fig. 3C: What is a position specific matrix? It is not clear to me that this analysis makes sense. It seems to compare apples and oranges (each protein vs other proteins) rather than comparing sequence features with properties such as affinity for actin. On the other hand the data in Fig 3C would be a good starting point to evaluate structure function relations by studying the structures to determine why some residues are more conserved than others.

Line 195: cite the figure. The binding experiment with a single unspecified actin filament concentration in Fig 3E is not a valid measure of

affinity. Again, the authors failed to simply look at the structures for hints about the variation in affinities for actin filaments.

Line 211: “Although a small number of substitutions were observed at these positions in certain species, these core residues remained largely unchanged, indicating strong evolutionary constraints (Figure 4a).” Again, a careful analysis of the structures should be reported to explain these observations.

Fig. 4C is unclear to me. Does the box with the MRCA’s mean that an early branching example like SHROOM3 is present is all bilateria and so on up the tree? Are any of the early arriving proteins lost on higher branches? The analysis does not consider a very large part of the metazoan phylogenetic tree.

Line 247: “they are involved in the regulation of diverse cellular functions (Figure 5b, Extended Data Figure 10 and Table 1).” The word “involved” in might be justified but how do you know they have “regulatory functions?” “Modulate actin” is vague. Some of these proteins have been characterized, revealing in most cases rather specific mechanisms of action. It is hard to justify lumping them in broad categories using rough genome annotations.

Line 263: “discovery of a new short linear F-actin binding motif (SFM), and provide first evidence for its biological role” is an overstatement. The VxxLxxxFE was discovered and exploited by one of the authors to make the actin filament binding peptide Lifeact. I do not understand how this paper explains the biological roles of VxxLxxxFE other than grouping the proteins in broad and possibly incorrect categories.

Lines 277-321: This discussion of possible functions of SFM motifs is mostly generalizations regarding localization to cellular compartments and “modulation of actin” rather than considering the more interesting question of actual mechanisms.

Line 512: “For the Phalloidin-stabilized F-actin-ITPKA complex, we initially applied 2 µl of 1 µM Phalloidin-stabilized F-actin to the grids. Subsequently, we added 2 µl of 10 µM ITPKA and mixed it directly on the grids immediately before plunge-freezing in a liquid ethane/propane.” Given the low affinity of ITPKA for actin, how did this procedure saturate the filaments with the ligand?

Line 1242: Explain the meaning of “plotting mean ipTM.”

Line 1248: Add a phrase to explain “HDBSCAN clustering.”

Line 1263: Add a phrase to explain “Poji radial profiles.”

Line 1265: “highest F-actin intensity” should be “highest fluorescent phalloidin intensity.”

Line 1084: Add a phrase to explain “position specific matrix (PSFM).”

Line 1105: Add a phrase to explain “dN/dS ratio.”

Fig. 1A: P4 does not look like leucine from the angle shown. Showing the map for the new structure would be informative Showing a stick figure of the peptide on a space filling model of actin would illustrate how the conserved residues interact with actin and residues on the other face of the helix that do not interact with actin. Some of the renderings in Extended Data Fig 1 are close to what is needed but do not show the binding sites of the four crucial residues. Helical wheels may assist in understanding evolutional constraints.

Style

What is the significance of the sentences in red font?

I recommend using actin filaments and actin monomers. G- and F-actin are jargon.

Line 54: “Many ABPs are inhibited in resting cells..” “Some” and “inactive” are better.

Line 70: “…thereby modulating polymerisation and filament dynamics.” This is vague and not informative about what is known about the mechanisms of action.

Signed, Tom Pollard

GUIDELINES FOR MANUSCRIPT SUBMISSION TO NATURE CELL BIOLOGY

READABILITY OF MANUSCRIPTS – Nature Cell Biology is read by cell biologists from diverse backgrounds, many of whom are not native English speakers. Authors should aim to communicate their findings clearly, explaining technical jargon that might be unfamiliar to non-specialists, and avoiding non-standard abbreviations. Titles and abstracts should concisely communicate the main findings of the study, and the background, rationale, results and conclusions should be clearly explained in the manuscript in a manner accessible to a broad cell biology audience. Nature Cell Biology uses British spelling.

MANUSCRIPT FORMAT – please follow the guidelines listed in our Guide to Authors regarding manuscript formats at Nature Cell Biology.

TITLE – should be no more than 100 characters including spaces, without punctuation and avoiding technical terms, abbreviations, and active verbs..

AUTHOR NAMES – should be given in full.

AUTHOR AFFILIATIONS – should be denoted with numerical superscripts (not symbols) preceding the names. Full addresses should be included, with US states in full and providing zip/post codes. The corresponding author is denoted by: "Correspondence should be addressed to [initials]."

ABSTRACT AND MAIN TEXT – please follow the guidelines that are specific to the format of your manuscript, as listed in our Guide to Authors (http://www.nature.com/ncb/pdf/ncb_gta.pdf) Briefly, Nature Cell Biology Articles, Resources and Technical Reports have 3500 words, including a 150 word abstract, and the main text is subdivided in Introduction, Results, and Discussion sections. Nature Cell Biology Letters have up to 2500 words, including a 180 word introductory paragraph (abstract), and the text is not subdivided in sections.

ACKNOWLEDGEMENTS – should be kept brief. Professional titles and affiliations are unnecessary. Grant numbers can be listed.

AUTHOR CONTRIBUTIONS – must be included after the Acknowledgements, detailing the contributions of each author to the paper (e.g. experimental work, project planning, data analysis etc.). Each author should be listed by his/her initials.

FINANCIAL AND NON-FINANCIAL COMPETING INTERESTS – the authors must include one of three declarations: (1) that they have no financial and non-financial competing interests; (2) that they have financial and non-financial competing interests; or (3) that they decline to respond, after the Author Contributions section. This statement will be published with the article, and in cases where financial and non-financial competing interests are declared, these will be itemized in a web supplement to the article. For further details please see https://www.nature.com/licenceforms/nrg/competing-interests.pdf.

REFERENCES – are limited to a total of 70 for Articles, Resources, Technical Reports; and 40 for Letters. This includes references in the main text and Methods combined. References must be numbered sequentially as they appear in the main text, tables and figure legends and Methods and must follow the precise style of Nature Cell Biology references. References only cited in the Methods should be numbered consecutively following the last reference cited in the main text. References only associated with Supplementary Information (e.g. in supplementary legends) do not count toward the total reference limit and do not need to be cited in numerical continuity with references in the main text. Only published papers can be cited, and each publication cited should be included in the numbered reference list, which should include the manuscript titles. Footnotes are not permitted.

METHODS – Nature Cell Biology publishes methods online. The methods section should be provided as a separate Word document, which will be copyedited and appended to the manuscript PDF, and incorporated within the HTML format of the paper.

Methods should be written concisely, but should contain all elements necessary to allow interpretation and replication of the results. As a guideline, Methods sections typically do not exceed 3,000 words. The Methods should be divided into subsections listing reagents and techniques. When citing previous methods, accurate references should be provided and any alterations should be noted. Information must be provided about: antibody dilutions, company names, catalogue numbers and clone numbers for monoclonal antibodies; sequences of RNAi and cDNA probes/primers or company names and catalogue numbers if reagents are commercial; cell line names, sources and information on cell line identity and authentication. Animal studies and experiments involving human subjects must be reported in detail, identifying the committees approving the protocols. For studies involving human subjects/samples, a statement must be included confirming that informed consent was obtained. Statistical analyses and information on the reproducibility of experimental results should be provided in a section titled “Statistics and Reproducibility”.

All Nature Cell Biology manuscripts submitted on or after March 21 2016 must include a Data availability statement at the end of the Methods section. For Springer Nature policies on data availability see http://www.nature.com/authors/policies/availability.html; for more information on this particular policy see http://www.nature.com/authors/policies/data/data-availability-statements-data-citations.pdf. The Data availability statement should include:

• Accession codes for primary datasets (generated during the study under consideration and designated as "primary accessions") and secondary datasets (published datasets reanalysed during the study under consideration, designated as "referenced accessions"). For primary accessions data should be made public to coincide with publication of the manuscript. A list of data types for which submission to community-endorsed public repositories is mandated (including sequence, structure, microarray, deep sequencing data) can be found here http://www.nature.com/authors/policies/availability.html#data.

• Unique identifiers (accession codes, DOIs or other unique persistent identifier) and hyperlinks for datasets deposited in an approved repository, but for which data deposition is not mandated (see here for details http://www.nature.com/sdata/data-policies/repositories).

• At a minimum, please include a statement confirming that all relevant data are available from the authors, and/or are included with the manuscript (e.g. as source data or supplementary information), listing which data are included (e.g. by figure panels and data types) and mentioning any restrictions on availability.

• If a dataset has a Digital Object Identifier (DOI) as its unique identifier, we strongly encourage including this in the Reference list and citing the dataset in the Methods.

We recommend that you upload the step-by-step protocols used in this manuscript to protocols.io. More details can found at https://www.protocols.io/help/publish-articles.

DISPLAY ITEMS – main display items are limited to 6-8 main figures and/or main tables for Articles, Resources, Technical Reports; and 5 main figures and/or main tables for Letters. For Supplementary Information see below.

FIGURES – Colour figure publication costs $600 for the first, and $300 for each subsequent colour figure. All panels of a multi-panel figure must be logically connected and arranged as they would appear in the final version. Unnecessary figures and figure panels should be avoided (e.g. data presented in small tables could be stated briefly in the text instead).

All imaging data should be accompanied by scale bars, which should be defined in the legend.
Cropped images of gels/blots are acceptable, but need to be accompanied by size markers, and to retain visible background signal within the linear range (i.e. should not be saturated). The boundaries of panels with low background have to be demarked with black lines. Splicing of panels should only be considered if unavoidable, and must be clearly marked on the figure, and noted in the legend with a statement on whether the samples were obtained and processed simultaneously. Quantitative comparisons between samples on different gels/blots are discouraged; if this is unavoidable, it should only be performed for samples derived from the same experiment with gels/blots were processed in parallel, which needs to be stated in the legend.

Figures should be provided at approximately the size that they are to be printed at (single column is 86 mm, double column is 170 mm) and should not exceed an A4 page (8.5 x 11"). Reduction to the scale that will be used on the page is not necessary, but multi-panel figures should be sized so that the whole figure can be reduced by the same amount at the smallest size at which essential details in each panel are visible. In the interest of our colour-blind readers we ask that you avoid using red and green for contrast in figures. Replacing red with magenta and green with turquoise are two possible colour-safe alternatives. Lines with widths of less than 1 point should be avoided. Sans serif typefaces, such as Helvetica (preferred) or Arial should be used. All text that forms part of a figure should be rewritable and removable.

We accept files from the following graphics packages in either PC or Macintosh format:

- For line art, graphs, charts and schematics we prefer Adobe Illustrator (.AI), Encapsulated PostScript (.EPS) or Portable Document Format (.PDF). Files should be saved or exported as such directly from the application in which they were made, to allow us to restyle them according to our journal house style.

- We accept PowerPoint (.PPT) files if they are fully editable. However, please refrain from adding PowerPoint graphical effects to objects, as this results in them outputting poor quality raster art. Text used for PowerPoint figures should be Helvetica (preferred) or Arial.

- We do not recommend using Adobe Photoshop for designing figures, but we can accept Photoshop generated (.PSD or .TIFF) files only if each element included in the figure (text, labels, pictures, graphs, arrows and scale bars) are on separate layers. All text should be editable in 'type layers' and line-art such as graphs and other simple schematics should be preserved and embedded within 'vector smart objects' - not flattened raster/bitmap graphics.

- Some programs can generate Postscript by 'printing to file' (found in the Print dialogue). If using an application not listed above, save the file in PostScript format or email our Art Editor, Allen Beattie for advice (a.beattie@nature.com).

Regardless of format, all figures must be vector graphic compatible files, not supplied in a flattened raster/bitmap graphics format, but should be fully editable, allowing us to highlight/copy/paste all text and move individual parts of the figures (i.e. arrows, lines, x and y axes, graphs, tick marks, scale bars etc.). The only parts of the figure that should be in pixel raster/bitmap format are photographic images or 3D rendered graphics/complex technical illustrations.

All placed images (i.e. a photo incorporated into a figure) should be on a separate layer and independent from any superimposed scale bars or text. Individual photographic images must be a minimum of 300+ DPI (at actual size) or kept constant from the original picture acquisition and not decreased in resolution post image acquisition. All colour artwork should be RGB format.

FIGURE LEGENDS – must not exceed 350 words for each figure to allow fit on a single printed NCB page together with the figure. They must include a brief title for the whole figure, and short descriptions of each panel with definitions of the symbols used, but without detailing methodology.

TABLES – main tables should be provided as individual Word files, together with a brief title and legend. For supplementary tables see below.

SUPPLEMENTARY INFORMATION – Supplementary information is material directly relevant to the conclusion of a paper, but which cannot be included in the printed version in order to keep the manuscript concise and accessible to the general reader. Supplementary information is an integral part of a Nature Cell Biology publication and should be prepared and presented with as much care as the main display item, but it must not include non-essential data or text, which may be removed at the editor's discretion. All supplementary material is fully peer-reviewed and published online as part of the HTML version of the manuscript. Supplementary Figures and Supplementary Notes are appended at the end of the main PDF of the published manuscript.

Supplementary items should relate to a main text figure, wherever possible, and should be mentioned sequentially in the main manuscript, designated as Supplementary Figure, Table, Video, or Note, and numbered continuously (e.g. Supplementary Figure 1, Supplementary Figure 2, Supplementary Table 1, Supplementary Table 2 etc.).

Unprocessed scans of all key data generated through electrophoretic separation techniques need to be presented in a supplementary figure that should be labelled and numbered as the final supplementary figure, and should be mentioned in every relevant figure legend. This figure does not count towards the total number of figures and is the only figure that can be displayed over multiple pages, but should be provided as a single file, in PDF or TIFF format. Data in this figure can be displayed in a relatively informal style, but size markers and the figures panels corresponding to the presented data must be indicated.

The total number of Supplementary Figures (not including the "unprocessed scans" Supplementary Figure) should not exceed the number of main display items (figures and/or tables (see our Guide to Authors and March 2012 editorial http://www.nature.com/ncb/authors/submit/index.html#suppinfo; http://www.nature.com/ncb/journal/v14/n3/index.html#ed). No restrictions

apply to Supplementary Tables or Videos, but we advise authors to be selective in including supplemental data.

Each Supplementary Figure should be provided as a single page and as an individual file in one of our accepted figure formats and should be presented according to our figure guidelines (see above). Supplementary Tables should be provided as individual Excel files. Supplementary Videos should be provided as .avi or .mov files up to 50 MB in size. Supplementary Figures, Tables and Videos much be accompanied by a separate Word document including titles and legends.

GUIDELINES FOR EXPERIMENTAL AND STATISTICAL REPORTING

REPORTING REQUIREMENTS – To improve the quality of methods and statistics reporting in our papers we have recently revised the reporting checklist we introduced in 2013. We are now asking all life sciences authors to complete two items: an Editorial Policy Checklist (found here https://www.nature.com/authors/policies/Policy.pdf) that verifies compliance with all required editorial policies and a reporting summary (found here https://www.nature.com/authors/policies/ReportingSummary.pdf) that collects information on experimental design and reagents. These documents are available to referees to aid the evaluation of the manuscript. Please note that these forms are dynamic 'smart pdfs' and must therefore be downloaded and completed in Adobe Reader. We will then flatten them for ease of use by the reviewers. If you would like to reference the guidance text as you complete the template, please access these flattened versions at http://www.nature.com/authors/policies/availability.html.

STATISTICS – Wherever statistics have been derived the legend needs to provide the n number (i.e. the sample size used to derive statistics) as a precise value (not a range), and define what this value represents. Error bars need to be defined in the legends (e.g. SD, SEM) together with a measure of centre (e.g. mean, median). Box plots need to be defined in terms of minima, maxima, centre, and percentiles. Ranges are more appropriate than standard errors for small data sets. Wherever statistical significance has been derived, precise p values need to be provided and the statistical test used needs to be stated in the legend. Statistics such as error bars must not be derived from n<3. For sample sizes of n<5 please plot the individual data points rather than providing bar graphs. Deriving statistics from technical replicate samples, rather than biological replicates is strongly discouraged. Wherever statistical significance has been derived, precise p values need to be provided and the statistical test stated in the legend.

Information on how many times each experiment was repeated independently with similar results needs to be provided in the legends and/or Methods for all experiments, and in particular wherever representative experiments are shown.

We strongly recommend the presentation of source data for graphical and statistical analyses as a separate Supplementary Table, and request that source data for all independent repeats are provided when representative experiments of multiple independent repeats, or averages of two independent experiments are presented. This supplementary table should be in Excel format, with data for different figures provided as different sheets within a single Excel file. It should be labelled and numbered as one of the supplementary tables, titled "Statistics Source Data", and mentioned in all relevant figure legends.

--------- Please don't hesitate to contact NCB@nature.com should you have queries about any of the above requirements ---------

Version 1:

Decision Letter:

*Please delete the link to your author homepage if you wish to forward this email to co-authors.

Dear Professor Windhorst,

I apologize for the delay. We had contacted both Reviewers #2 and #3 for additional feedback, particularly regarding Reviewer #1's current concerns. We have appended Reviewer #2's additional feedback in their comments below.

Your manuscript, "Discovery of a new evolutionarily conserved short linear F-actin binding motif", has now been seen by our original referees, who are experts in cryoEM, mechanobiology (referee 1); protein self-assembly, computational methods (referee 2); and actin, structure (referee 3). As you will see from their comments (attached below) they find this work of interest, but have raised some important points. Although we are also very interested in this study, we believe that their concerns should be addressed before we can consider publication in Nature Cell Biology.

Nature Cell Biology editors discuss the referee reports in detail within the editorial team, including the chief editor, to identify key referee points that should be addressed with priority, and requests that are overruled as being beyond the scope of the current study. To guide the scope of the revisions, I have listed these points below. We are committed to providing a fair and constructive peer-review process, so please feel free to contact me if you would like to discuss any of the referee comments further.

In particular, it would be essential to:

A) Compare D-loop structures in 7AD9 with the ensemble of conformations (as suggested by Reviewer #2 to address Reviewer #1's concerns)

B) additional discussion on potential future directions regarding potential effects of filament softening (Reviewer #2 to address Reviewer #1's concerns)

C) Provide additional explanations and parameters of the MD simulations, and compare with Iyer et al (doi: 10.1073/pnas.2421467122) (see comments from Reviewer #3)

D) All other referee concerns pertaining to strengthening existing data, providing controls, methodological details, clarifications and textual changes, should also be addressed.

E) Finally please pay close attention to our guidelines on statistical and methodological reporting (listed below) as failure to do so may delay the reconsideration of the revised manuscript.In particular please provide:

- a Supplementary Figure including unprocessed images of all gels/blots in the form of a multi-page pdf file. Please ensure that blots/gels are labeled and the sections presented in the figures are clearly indicated.

- a Supplementary Table including all numerical source data in Excel format, with data for different figures provided as different sheets within a single Excel file. The file should include source data giving rise to graphical representations and statistical descriptions in the paper and for all instances where the figures present representative experiments of multiple independent repeats, the source data of all repeats should be provided.

In contrast, although we agree with referee #1 that additional experimentation to justify MD simulations regarding alterations in actin stiffness would provide valuable insights, we consider this point to be beyond the scope of the present study. Thus, addressing it experimentally will not be necessary for reconsideration of the manuscript at this journal but would welcome further discussion and caveats as appropriate.

We therefore invite you to take these points into account when revising the manuscript. In addition, when preparing the revision please:

- ensure that it conforms to our format instructions and publication policies (see below and www.nature.com/nature/authors/).

- provide a point-by-point rebuttal to the full referee reports verbatim, as provided at the end of this letter.
- for any revision that includes light microscopy data, we ask our authors to please include a completed light microscopy reporting table https://www.nature.com/documents/Light_microscopy_reporting_table.xlsx to ensure the methods are described thoroughly. The table will be available to reviewers and ultimately published should the manuscript be accepted at the journal.

Nature Cell Biology is committed to improving transparency in authorship. As part of our efforts in this direction, we are now requesting that all authors identified as 'corresponding author' on published papers create and link their Open Researcher and Contributor Identifier (ORCID) with their account on the Manuscript Tracking System (MTS), prior to acceptance. ORCID helps the scientific community achieve unambiguous attribution of all scholarly contributions. You can create and link your ORCID from the home page of the MTS by clicking on 'Modify my Springer Nature account'. For more information please visit please visit www.springernature.com/orcid.

Please submit the revised manuscript files and the point-by-point rebuttal to the referee comments using this link:

Link Redacted

*This url links to your confidential home page and associated information about manuscripts you may have submitted or be reviewing for us. If you wish to forward this email to co-authors, please delete the link to your homepage.

We would like to receive the revision within four weeks. If submitted within this time period, reconsideration of the revised manuscript will not be affected by related studies published elsewhere, or accepted for publication in Nature Cell Biology in the meantime. We would be happy to consider a revision even after this timeframe, but in that case we will consider the published literature at the time of resubmission when assessing the file.

We hope that you will find our referees' comments, and editorial guidance helpful. Please do not hesitate to contact me if there is anything you would like to discuss.

Best wishes,

Daryl

Daryl Jason Verzosa David, PhD

Senior Editor, Nature Cell Biology
Advisory Editor, npj Biological Physics and Mechanics
Nature Portfolio

Heidelberger Platz 3, 14197 Berlin, Germany
Email: daryl.david@nature.com
ORCID: https://orcid.org/0000-0002-9253-4805

Reviewers' Comments:

Reviewer #1 (Remarks to the Author):

In their revised manuscript, Paraschiakos and colleagues were highly responsive to our comments and those of the other reviewers.

Many aspects of the paper were substantively improved, including rigorous presentation of the structural data, and a much more comprehensible discussion of the evolutionary implications of the work. The inclusion of the phosphosite analysis and comparison with other actin-bound structures also fills out the paper nicely.

There is, however, one new area of major concern. In response to a reviewer comment, the authors have now included analysis of molecular dynamics simulations (MD) suggesting that SFMs impact the bending stiffness of actin filaments. This is a quite striking and potentially very important result, but it is in our opinion weakly supported. We believe rigorously making this claim would require experimental support, which could be garnered through either fluorescence microscopy experiments in the presence / absence of SFM (e.g. as done in ref. 50 for cofilin) or by analyzing the curvature of filament segments in cryo-EM images (as done in ref. 48) and comparing them to bare filaments. Both of these would be non-trivial exercises.

A major reason for this skepticism is the potential for the MD simulations to be sensitive to the starting conformation of the D loop, which as the authors note has previously been linked to actin bending dynamics. The D loop tends to be the most poorly resolved portion of actin in cryo-EM reconstructions, which has been characterized through extensive local masked classification to represent conformational variability in prior studies. Inspecting Fig. 1a, it is apparent that the authors built the D loop of the ITPKA structure in a different conformation than that of the Lifeact structure (7AD9). Examining Fig. 5e, it appears that the short region of the D loop displaying rigidification in the presence of SFMs is within the stretch that the authors modeled as a different conformation. However, the model-map fit shown in ED Fig. 1a shows poor density for this region of the D loop, suggesting that multiple conformations could potentially be consistent with the density.

To at least moderately enhance confidence in the existing approach, the authors could test the robustness of their analysis by performing simulations with different starting D loop conformations (e.g. that in the Lifeact structure). It is also not clear from the Methods what the authors used as their “bare actin structure”: was it one of the structures presented in this paper where the SFM was computationally removed, or another structure from the PDB?

A path forward that would satisfy us is to simply remove the MD analysis / claims about effects on actin mechanics. We believe it is is ancillary to the overall findings of the paper, and it could be the subject of a future study featuring an experimental component as outlined above. However, we realize this may put us at odds with other reviewers, so we encourage the editor to weigh in on this point.

Reviewer #2 (Remarks to the Author):

I am happy with the changes made. I recommend publication.

Reviewer #2’s further comments on Reviewer #1’s current concerns:
The major skepticism of the MD results expressed by the reviewer arises from (1) the difference in D-loop conformation modeled in the ITPKA-bound filament compared to the Lifeact-bound filament structure (7AD9) used as a template, and (2) the lack of experimental results directly supporting filament softening due to ITPKA interactions with the D-loop.

To address the first concern, I suggest that the authors explicitly compare the D-loop structure in 7AD9 with the ensemble of D-loop conformations rendered in Figure 5e. I believe that at least one of the simulated conformations would match well with the D-loop conformation observed in 7AD9. The reviewer’s concern appears to be influenced by reliance on a single static conformation derived from cryo-EM reconstruction, whereas MD simulations—although initiated from a single structure—naturally generate an ensemble of thermally accessible D-loop conformations (at 310K!). In this context, the modest difference in D-loop conformation between the starting structure and 7AD9 does not represent a significant limitation of the simulations.

Regarding the second concern—the lack of direct experimental evidence supporting filament softening due to ITPKA interactions with the D-loop—I emphasize that the role of MD simulations here is not to replace experiments, but to provide mechanistic insight and testable predictions. The authors should include a line in the text about this while discussing the MD results. Within the context of prior experimental and computational studies that have established a link between D-loop flexibility and filament bending rigidity, the MD results presented are mechanically well grounded and internally consistent.

Reviewer #3 (Remarks to the Author):

Windhorst et al. revised

The authors carried out additional work and made extensive revisions in response to the first round of review. I am generally satisfied with the constructive responses to my review. The new work provides valuable additional information about the interactions of SFM with actin filaments. However, some of the extensive new sections (conveniently in red font) are still rough and need revision. I am now enthusiastic about the publication of this paper after suitable revision.

Line 2: “F-actin” not replaced with “actin filaments” as promised. Many other examples are found throughout the text.

Line 255: “insides” should be “insights.”

Line 295: “(Figure 4c)” should be “(Figure 5b).”

Order of text: The results might be easier to understand if excellent new Fig 4 and the associated text were presented before Fig 3 and its text.

Fig. 4: These structure renderings are an important addition, but many of the labels are difficult to read, particularly the purple font on the

space-filling model. Enlarge the labels for the subunits in panels a and b as “Subunit A0” and “Subunit A-2.” Consider showing panels a and b as stereo pairs, which would be more informative. Alternatively, a supplemental movie might help. The ribbon diagrams in panels D-F would be better with darker colors. The blue sidechain labels are too faint. Label panels g and h with larger and darker “ITPKA” and “USP54 M1”. The helical wheels are helpful, but labels are essential to identify the upper residues as part of actin.

Line 281: Give the temperature of the simulations.

Fig. 5: The MD simulations and their analysis are another valuable addition. However, the most of the panels and labels are too small, especially panel g. The plots in Panel c clearly show SFM binding reduces D-loop fluctuations, but these differences are not apparent to my eye in panel e. The D-loop region of panel C should be shown at larger size (expanded region of x-axis) in an inset to show which residues are affected. Neither of these panels shows how SFM binding affects interactions between the side chains of D-loop residues with the adjacent actin subunit. Panel f needs a y-axis label “Residue interactions along the long pitch helix.” The legend for panel f must explain that the x-axis labels are pairs of interacting residues in subunits along the long-pitch helix with the lower subunit first and upper residue second. Oddly, the most important D-loop residue making long-pitch interactions, M44, is not considered. Neither is V43.

Line 304: what are “inter-subunit contact edges?”

Line 306: the text says “both peptides consistently reduced the inter-protomer contact of residues Q49–E167,” but Fig 5F shows this is largely restricted to the first 15 ns of the simulation. Explain this more clearly. Might this be due to the shifting conformations of the actin subunits during the initial part of the simulation, as noted in the next paragraph?

Comment on MD simulations: The authors would not have been aware of a recent paper by Iyer et al. (PNAS Dec 2025), but may want to take their findings into consideration during final revision. Iyer et al. also simulated a 13-mer Mg-ADP-actin filament at 310 K and found that the conformations of the subunits and the helical twist fluctuate on a nanosecond time scale, only equilibrating after about 200 ns to a different structure than the starting cryo-EM structure. Did you observe these fluctuations in subunit conformations in your MD simulations of the actin filament alone or with SFM peptides? Did the fluctuations influence SFM peptide binding or longitudinal interactions between the subunits? Might the fact that your simulations were during the transition between the frozen and warm state have accounted for the variability over time in Fig. 5F?

Lines 311-314: Confusing. Which actin subunits have these changes? Between i+1 or i-1? Spell this out for each pair in the legend. What do the percentages mean?

Line 456: replace “share identical interactions within..” with “interact with the same residues in..”

Line 523: delete “strongly.” This is an inappropriate construction.

Style: The text is clear, but many sentences could be more concise. For example on Line 242 “Our data depicted in Figure 3e – h reveal that the M1 mutants of USP54 and SHROOM3 exhibit increased binding strength to actin filaments compared to their wild-type counterparts” could be “The affinities of M1 mutants of USP54 and SHROOM3 for actin filaments are higher than their wild-type counterparts (Figure 3e).” Many other sentences could be more concise.

Some figure legends such as 5 are too long. Just explain the panels and leave the interpretation to the text as done in Figure 4.

Signed, Tom Pollard

GUIDELINES FOR SUBMISSION OF NATURE CELL BIOLOGY ARTICLES

READABILITY OF MANUSCRIPTS – Nature Cell Biology is read by cell biologists from diverse backgrounds, many of whom are not native English speakers. Authors should aim to communicate their findings clearly, explaining technical jargon that might be unfamiliar to non-specialists, and avoiding non-standard abbreviations. Titles and abstracts should concisely communicate the main findings of the study, and the background, rationale, results and conclusions should be clearly explained in the manuscript in a manner accessible to a broad cell biology audience. Nature Cell Biology uses British spelling.

ARTICLE FORMAT

TITLE – should be no more than 100 characters including spaces, without punctuation and avoiding technical terms, abbreviations, and active verbs..

AUTHOR NAMES – should be given in full.

AUTHOR AFFILIATIONS – should be denoted with numerical superscripts (not symbols) preceding the names. Full addresses should be included, with US states in full and providing zip/post codes. The corresponding author is denoted by: "Correspondence should be addressed to [initials]."

ABSTRACT – should not exceed 150 words and should be unreferenced. This paragraph is the most visible part of the paper and should briefly outline the background and rationale for the work, and accurately summarize the main results and conclusions. Key genes,

proteins and organisms should be specified to ensure discoverability of the paper in online searches.

TEXT – the main text consists of the Introduction, Results, and Discussion sections and must not exceed 3500 words including the abstract. The Introduction should expand on the background relating to the work. The Results should be divided in subsections with subheadings, and should provide a concise and accurate description of the experimental findings. The Discussion should expand on the findings and their implications. All relevant primary literature should be cited, in particular when discussing the background and specific findings.

ACKNOWLEDGEMENTS – should be kept brief. Professional titles and affiliations are unnecessary. Grant numbers can be listed.

AUTHOR CONTRIBUTIONS – must be included after the Acknowledgements, detailing the contributions of each author to the paper (e.g. experimental work, project planning, data analysis etc.). Each author should be listed by his/her initials.

FINANCIAL AND NON-FINANCIAL COMPETING INTERESTS – the authors must include one of three declarations: (1) that they have no financial and non-financial competing interests; (2) that they have financial and non-financial competing interests; or (3) that they decline to respond, after the Author Contributions section. This statement will be published with the article, and in cases where financial and non-financial competing interests are declared, these will be itemized in a web supplement to the article. For further details please see https://www.nature.com/licenceforms/nrg/competing-interests.pdf.

REFERENCES – are limited to a total of 70 in the main text and Methods combined,. They must be numbered sequentially as they appear in the main text, tables and figure legends and Methods and must follow the precise style of Nature Cell Biology references. References only cited in the Methods should be numbered consecutively following the last reference cited in the main text. References only associated with Supplementary Information (e.g. in supplementary legends) do not count toward the total reference limit and do not need to be cited in numerical continuity with references in the main text. Only published papers can be cited, and each publication cited should be included in the numbered reference list, which should include the manuscript titles. Footnotes are not permitted.

METHODS – Nature Cell Biology publishes methods online. The methods section should be provided as a separate Word document, which will be copyedited and appended to the manuscript PDF, and incorporated within the HTML format of the paper.

Methods should be written concisely, but should contain all elements necessary to allow interpretation and replication of the results. As a guideline, Methods sections typically do not exceed 3,000 words. The Methods should be divided into subsections listing reagents and techniques. When citing previous methods, accurate references should be provided and any alterations should be noted. Information must be provided about: antibody dilutions, company names, catalogue numbers and clone numbers for monoclonal antibodies; sequences of RNAi and cDNA probes/primers or company names and catalogue numbers if reagents are commercial; cell line names, sources and information on cell line identity and authentication. Animal studies and experiments involving human subjects must be reported in detail, identifying the committees approving the protocols. For studies involving human subjects/samples, a statement must be included confirming that informed consent was obtained. Statistical analyses and information on the reproducibility of experimental results should be provided in a section titled “Statistics and Reproducibility”.

All Nature Cell Biology manuscripts submitted on or after March 21 2016, must include a Data availability statement as a separate section after Methods but before references, under the heading "Data Availability”. For Springer Nature policies on data availability see http://www.nature.com/authors/policies/availability.html; for more information on this particular policy see http://www.nature.com/authors/policies/data/data-availability-statements-data-citations.pdf. The Data availability statement should include:

• Accession codes for primary datasets (generated during the study under consideration and designated as "primary accessions") and secondary datasets (published datasets reanalysed during the study under consideration, designated as "referenced accessions"). For primary accessions data should be made public to coincide with publication of the manuscript. A list of data types for which submission to community-endorsed public repositories is mandated (including sequence, structure, microarray, deep sequencing data) can be found here http://www.nature.com/authors/policies/availability.html#data.

• Unique identifiers (accession codes, DOIs or other unique persistent identifier) and hyperlinks for datasets deposited in an approved repository, but for which data deposition is not mandated (see here for details http://www.nature.com/sdata/data-policies/repositories).

• At a minimum, please include a statement confirming that all relevant data are available from the authors, and/or are included with the manuscript (e.g. as source data or supplementary information), listing which data are included (e.g. by figure panels and data types) and mentioning any restrictions on availability.

• If a dataset has a Digital Object Identifier (DOI) as its unique identifier, we strongly encourage including this in the Reference list and citing the dataset in the Methods.

We recommend that you upload the step-by-step protocols used in this manuscript to protocols.io. More details can found at https://www.protocols.io/help/publish-articles.

DISPLAY ITEMS – main display items are limited to 6-8 main figures and/or main tables. For Supplementary Information see below.

FIGURES – Colour figure publication costs $395 per colour figure. All panels of a multi-panel figure must be logically connected and arranged as they would appear in the final version. Unnecessary figures and figure panels should be avoided (e.g. data presented in small tables could be stated briefly in the text instead).

All imaging data should be accompanied by scale bars, which should be defined in the legend.
Cropped images of gels/blots are acceptable, but need to be accompanied by size markers, and to retain visible background signal within the linear range (i.e. should not be saturated). The boundaries of panels with low background have to be demarked with black lines. Splicing of panels should only be considered if unavoidable, and must be clearly marked on the figure, and noted in the legend with a

statement on whether the samples were obtained and processed simultaneously. Quantitative comparisons between samples on different gels/blots are discouraged; if this is unavoidable, it has be performed for samples derived from the same experiment with gels/blots were processed in parallel, which needs to be stated in the legend.

Figures should be provided at approximately the size that they are to be printed at (single column is 86 mm, double column is 170 mm) and should not exceed an A4 page (8.5 x 11"). Reduction to the scale that will be used on the page is not necessary, but multi-panel figures should be sized so that the whole figure can be reduced by the same amount at the smallest size at which essential details in each panel are visible. In the interest of our colour-blind readers we ask that you avoid using red and green for contrast in figures. Replacing red with magenta and green with turquoise are two possible colour-safe alternatives. Lines with widths of less than 1 point should be avoided. Sans serif typefaces, such as Helvetica (preferred) or Arial should be used. All text that forms part of a figure should be rewritable and removable.

We accept files from the following graphics packages in either PC or Macintosh format:

- For line art, graphs, charts and schematics we prefer Adobe Illustrator (.AI), Encapsulated PostScript (.EPS) or Portable Document Format (.PDF). Files should be saved or exported as such directly from the application in which they were made, to allow us to restyle them according to our journal house style.

- We accept PowerPoint (.PPT) files if they are fully editable. However, please refrain from adding PowerPoint graphical effects to objects, as this results in them outputting poor quality raster art. Text used for PowerPoint figures should be Helvetica (preferred) or Arial.

- We do not recommend using Adobe Photoshop for designing figures, but we can accept Photoshop generated (.PSD or .TIFF) files only if each element included in the figure (text, labels, pictures, graphs, arrows and scale bars) are on separate layers. All text should be editable in 'type layers' and line-art such as graphs and other simple schematics should be preserved and embedded within 'vector smart objects' - not flattened raster/bitmap graphics.

- Some programs can generate Postscript by 'printing to file' (found in the Print dialogue). If using an application not listed above, save the file in PostScript format or email our Art Editor, Allen Beattie for advice (a.beattie@nature.com).

Regardless of format, all figures must be vector graphic compatible files, not supplied in a flattened raster/bitmap graphics format, but should be fully editable, allowing us to highlight/copy/paste all text and move individual parts of the figures (i.e. arrows, lines, x and y axes, graphs, tick marks, scale bars etc). The only parts of the figure that should be in pixel raster/bitmap format are photographic images or 3D rendered graphics/complex technical illustrations.

All placed images (i.e. a photo incorporated into a figure) should be on a separate layer and independent from any superimposed scale bars or text. Individual photographic images must be a minimum of 300+ DPI (at actual size) or kept constant from the original picture acquisition and not decreased in resolution post image acquisition. All colour artwork should be RGB format.

FIGURE LEGENDS – must not exceed 350 words for each figure to allow fit on a single printed NCB page together with the figure. They must include a brief title for the whole figure, and short descriptions of each panel with definitions of the symbols used, but without detailing methodology.

TABLES – main tables should be provided as individual Word files, together with a brief title and legend. For supplementary tables see below.

SUPPLEMENTARY INFORMATION – Supplementary information is material directly relevant to the conclusion of a paper, but which cannot be included in the printed version in order to keep the manuscript concise and accessible to the general reader. Supplementary information is an integral part of a Nature Cell Biology publication and should be prepared and presented with as much care as the main display item, but it must not include non-essential data or text, which may be removed at the editor's discretion. All supplementary material is fully peer-reviewed and published online as part of the HTML version of the manuscript. Supplementary Figures and Supplementary Notes are appended at the end of the main PDF of the published manuscript.

Supplementary items should relate to a main text figure, wherever possible, and should be mentioned sequentially in the main manuscript, designated as Supplementary Figure, Table, Video, or Note, and numbered continuously (e.g. Supplementary Figure 1, Supplementary Figure 2, Supplementary Table 1, Supplementary Table 2 etc.).

Unprocessed scans of all key data generated through electrophoretic separation techniques need to be presented in a supplementary figure that should be labeled and numbered as the final supplementary figure, and should be mentioned in every relevant figure legend. This figure does not count towards the total number of figures and is the only figure that can be displayed over multiple pages, but should be provided as a single file, in PDF or TIFF format. Data in this figure can be displayed in a relatively informal style, but size markers and the figures panels corresponding to the presented data must be indicated.

The total number of Supplementary Figures (not including the "unprocessed scans" Supplementary Figure) should not exceed the number of main display items (figures and/or tables (see our Guide to Authors and March 2012 editorial http://www.nature.com/ncb/authors/submit/index.html#suppinfo; http://www.nature.com/ncb/journal/v14/n3/index.html#ed). No restrictions apply to Supplementary Tables or Videos, but we advise authors to be selective in including supplemental data.

Each Supplementary Figure should be provided as a single page and as an individual file in one of our accepted figure formats and should be presented according to our figure guidelines (see above). Supplementary Tables should be provided as individual Excel files. Supplementary Videos should be provided as .avi or .mov files up to 50 MB in size. Supplementary Figures, Tables and Videos much be accompanied by a separate Word document including titles and legends.

GUIDELINES FOR EXPERIMENTAL AND STATISTICAL REPORTING

REPORTING REQUIREMENTS – To improve the quality of methods and statistics reporting in our papers we have recently revised the reporting checklist we introduced in 2013. We are now asking all life sciences authors to complete two items: an Editorial Policy Checklist (found here https://www.nature.com/authors/policies/Policy.pdf) that verifies compliance with all required editorial policies and a Reporting Summary (found here https://www.nature.com/authors/policies/ReportingSummary.pdf) that collects information on experimental design and reagents. These documents are available to referees to aid the evaluation of the manuscript. Please note that these forms are dynamic 'smart pdfs' and must therefore be downloaded and completed in Adobe Reader. We will then flatten them for ease of use by the reviewers. If you would like to reference the guidance text as you complete the template, please access these flattened versions at http://www.nature.com/authors/policies/availability.html.

STATISTICS – Wherever statistics have been derived the legend needs to provide the n number (i.e. the sample size used to derive statistics) as a precise value (not a range), and define what this value represents. Error bars need to be defined in the legends (e.g. SD, SEM) together with a measure of centre (e.g. mean, median). Box plots need to be defined in terms of minima, maxima, centre, and percentiles. Ranges are more appropriate than standard errors for small data sets. Wherever statistical significance has been derived, precise p values need to be provided and the statistical test used needs to be stated in the legend. Statistics such as error bars must not be derived from n<3. For sample sizes of n<5 please plot the individual data points rather than providing bar graphs. Deriving statistics from technical replicate samples, rather than biological replicates is strongly discouraged. Wherever statistical significance has been derived, precise p values need to be provided and the statistical test stated in the legend.

Information on how many times each experiment was repeated independently with similar results needs to be provided in the legends and/or Methods for all experiments, and in particular wherever representative experiments are shown.

We strongly recommend the presentation of source data for graphical and statistical analyses as a separate Supplementary Table, and request that source data for all independent repeats are provided when representative experiments of multiple independent repeats, or averages of two independent experiments are presented. This supplementary table should be in Excel format, with data for different figures provided as different sheets within a single Excel file. It should be labelled and numbered as one of the supplementary tables, titled "Statistics Source Data", and mentioned in all relevant figure legends.

--------- Please don't hesitate to contact NCB@nature.com should you have queries about any of the above requirements ---------

Version 2:

Decision Letter:

Our ref: NCB-A57869B

20th February 2026

Dear Dr. Windhorst,

Thank you for submitting your revised manuscript "Discovery of a new evolutionarily conserved short linear actin filament binding motif" (NCB-A57869B). It has now been seen by the original referees and their comments are below. The reviewers find that the paper has improved in revision, and therefore we'll be happy in principle to publish it in Nature Cell Biology, pending minor revisions to satisfy the referees' final requests and to comply with our editorial and formatting guidelines.

We are now performing detailed checks on your paper and will send you a checklist detailing our editorial and formatting requirements in about a week. Please do not upload the final materials and make any revisions until you receive this additional information from us.

Thank you again for your interest in Nature Cell Biology Please do not hesitate to contact me if you have any questions.

Sincerely,
Daryl

Daryl Jason Verzosa David, PhD

Senior Editor, Nature Cell Biology
Advisory Editor, npj Biological Physics and Mechanics
Nature Portfolio

Heidelberger Platz 3, 14197 Berlin, Germany
Email: daryl.david@nature.com
ORCID: https://orcid.org/0000-0002-9253-4805

Reviewer #2 (Remarks to the Author):

I am happy with the revisions and recommend publication.

Version 3:

Decision Letter:

Dear Dr Windhorst,

I am pleased to inform you that your manuscript, "Evolutionarily conserved short linear motifs drive actin filament binding", has now been accepted for publication in Nature Cell Biology.

Thank you for sending us the final manuscript files to be processed for print and online production, and for returning the manuscript checklists and other forms. Your manuscript will now be passed to our production team who will be in contact with you if there are any questions with the production quality of supplied figures and text.

Over the next few weeks, your paper will be copyedited to ensure that it conforms to Nature Cell Biology style. Once your paper is typeset, you will receive an email with a link to choose the appropriate publishing options for your paper and our Author Services team will be in touch regarding any additional information that may be required.

After the grant of rights is completed, you will receive a link to your electronic proof via email with a request to make any corrections within 48 hours. If, when you receive your proof, you cannot meet this deadline, please inform us at rjsproduction@springernature.com immediately.

You will not receive your proofs until the publishing agreement has been received through our system.

Due to the importance of these deadlines, we ask that you please let us know now whether you will be difficult to contact over the next month. If this is the case, we ask you provide us with the contact information (email, phone and fax) of someone who will be able to check the proofs on your behalf, and who will be available to address any last-minute problems.

If you have any questions about our publishing options, costs, Open Access requirements, or our legal forms, please contact ASJournals@springernature.com

Once your paper has been scheduled for online publication, the Nature press office will be in touch to confirm the details. An online order form for reprints of your paper is available at https://www.nature.com/reprints/author-reprints.html. All co-authors, authors' institutions and authors' funding agencies can order reprints using the form appropriate to their geographical region.

Publication is conditional on the manuscript not being published elsewhere and on there being no announcement of this work to any media outlet until the online publication date in Nature Cell Biology.

You may wish to make your media relations office aware of your accepted publication, in case they consider it appropriate to organize some internal or external publicity. Once your paper has been scheduled you will receive an email confirming the publication details. This is normally 3-4 working days in advance of publication. If you need additional notice of the date and time of publication, please let the production team know when you receive the proof of your article to ensure there is sufficient time to coordinate. Further information on our embargo policies can be found here: https://www.nature.com/authors/policies/embargo.html

Please note that *Nature Cell Biology* is a Transformative Journal (TJ). Authors may publish their research with us through the traditional subscription access route or make their paper immediately open access through payment of an article-processing charge (APC). Authors will not be required to make a final decision about access to their article until it has been accepted. Find out more about Transformative Journals

Authors may need to take specific actions to achieve compliance with funder and institutional open access mandates. If your research is supported by a funder that requires immediate open access (e.g. according to Plan S principles or the NIH public access policy) then you should select the gold OA route, and we will direct you to the compliant route where possible. Because authors warrant under our subscription licensing terms that they haven't committed to licensing any version of their article under a licence inconsistent with the terms of our agreement – including the applicable embargo period – publication under the subscription model isn't suitable for authors whose funders require no embargo.

To assist our authors in disseminating their research to the broader community, our SharedIt initiative provides you with a unique shareable link that will allow anyone (with or without a subscription) to read the published article. Recipients of the link with a subscription will also be able to download and print the PDF.

If your paper includes color figures, please be aware that in order to help cover some of the additional cost of four-color reproduction, Nature Portfolio charges our authors a fee for the printing of their color figures. Please contact our offices for exact pricing and details.

As soon as your article is published, you will receive an automated email with your shareable link.

If you have not already done so, we strongly recommend that you upload the step-by-step protocols used in this manuscript to protocols.io (https://protocols.io), an open online resource that allows researchers to share their detailed experimental know-how. All uploaded protocols are made freely available and are assigned DOIs for ease of citation. Protocols and Nature Portfolio journal papers in which they are used can be linked to one another, and this link is clearly and prominently visible in the online versions of both. Authors who performed the specific experiments can act as primary authors for the Protocol as they will be best placed to share the methodology

details, but the Corresponding Author of the present research paper should be included as one of the authors. By uploading your Protocols onto protocols.io, you are enabling researchers to more readily reproduce or adapt the methodology you use, as well as increasing the visibility of your protocols and papers. You can also establish a dedicated workspace to collect your lab Protocols. Further information can be found at https://www.protocols.io/help/publish-articles.

You can use a single sign-on for all your accounts, view the status of all your manuscript submissions and reviews, access usage statistics for your published articles and download a record of your refereeing activity for the Nature Portfolio.

Nature Cell Biology encourages authors presenting evidence for cell, biological, molecular, and genetic interactions to consider communicating these findings using Biofactoid (https://biofactoid.org/). This tool helps users share a searchable representation of interactions (e.g. binding, gene expression, post-translational modification) between genes, gene products, or chemicals. Information added to Biofactoid, with author attribution, is shared on social media and public databases, such as Pathway Commons, where it can be discovered and analyzed in the context of a large and growing corpus of knowledge.

Please feel free to contact us if you have any questions.

With kind regards,
Daryl

Daryl Jason Verzosa David, PhD

Senior Editor, Nature Cell Biology
Advisory Editor, npj Biological Physics and Mechanics
Nature Portfolio

Heidelberger Platz 3, 14197 Berlin, Germany
Email: daryl.david@nature.com
ORCID: https://orcid.org/0000-0002-9253-4805

Click here if you would like to recommend Nature Cell Biology to your librarian
http://www.nature.com/subscriptions/recommend.html#forms

** Visit the Springer Nature Editorial and Publishing website at www.springernature.com/editorial-and-publishing-jobs for more information about our career opportunities. If you have any questions please click here.**

Open Access This Peer Review File is licensed under a Creative Commons Attribution 4.0 International License, which permits use, sharing, adaptation, distribution and reproduction in any medium or format, as long as you give appropriate credit to the original author(s) and the source, provide a link to the Creative Commons license, and indicate if changes were made.
In cases where reviewers are anonymous, credit should be given to 'Anonymous Referee' and the source.
The images or other third party material in this Peer Review File are included in the article's Creative Commons license, unless indicated otherwise in a credit line to the material. If material is not included in the article's Creative Commons license and your intended use is not permitted by statutory regulation or exceeds the permitted use, you will need to obtain permission directly from the copyright holder.
To view a copy of this license, visit https://creativecommons.org/licenses/by/4.0/

Zentrum für Experimentelle Medizin
Institut für Biochemie und Signaltransduktion (IBS)

Prof. Dr. Sabine Windhorst

Martinistraße 52
20246 Hamburg

Gebäude N25, EG, Raum 00.1.011
Telefon: +49 (0) 40 7410-56013
Fax: +49 (0) 40 7410-56818
s.windhorst@uke.de
www.uke.de

Universitätsklinikum Hamburg-Eppendorf | Martinistraße 52 | 20246 Hamburg
Zentrum für Experimentelle Medizin – Institut für Biochemie und Signaltransduktion

Nature Cell Biology

Hamburg, 12.12.2025

Re-submission of the manuscript "Discovery of a new evolutionarily conserved short linear F-actin binding motif"

Dear Dr. Kary,

Thank you very much for giving us the opportunity to re-submit a revised version of our manuscript "Discovery of a new evolutionarily conserved short linear F-actin binding motif"

We are also grateful full to the reviewer for carefully reading our manuscript, and for their expert suggestions. We addressed all issues, and marked the changes inside the manuscript in red.

Comment of the Editors:
In particular, it would be essential to:

A) Carry out additional structure/function studies to address the questions of all 3 reviewers about the structure of SFM peptides bound to actin filaments.

B) Clarify evolutionary analysis (reviewer 1)

C) All other referee concerns pertaining to strengthening existing data, providing controls, methodological details, presentation of data including cryo-EM data, clarifications and textual changes, should also be addressed.

Gerichtsstand: Hamburg
Körperschaft des öffentlichen Rechts
USt-Id: DE 21 8618 948

Bank: Hamburger Sparkasse AG
BIC: HASP DEHH | Konto: 1280 1706 38
IBAN: DE91 2005 0550 1280 1706 38

Vorstandsmitglieder:
Prof. Dr. Christian Gerloff (Vorstandsvorsitzender)
Joachim Prölß
Prof. Dr. Blanche Schwappach-Pignataro
Corinna Wriedt

Universitätsklinikum Hamburg-Eppendorf

D) Finally please pay close attention to our guidelines on statistical and methodological reporting (listed below) as failure to do so may delay the reconsideration of the revised manuscript. In particular please provide:

- a Supplementary Figure including unprocessed images of all gels/blots in the form of a multi-page pdf file. Please ensure that blots/gels are labeled and the sections presented in the figures are clearly indicated.

- a Supplementary Table including all numerical source data in Excel format, with data for different figures provided as different sheets within a single Excel file. The file should include source data giving rise to graphical representations and statistical descriptions in the paper and for all instances where the figures present representative experiments of multiple independent repeats, the source data of all repeats should be provided.

We thank the editors for their summary and have focused on the issues raised. The detailed changes are explained in our point-by-point response:

Reviewer #1:

In their paper "Discovery of a new evolutionarily conserved short linear F-actin binding motif", Paraschiakos and colleagues present evidence for a widely distributed conserved "Short Linear Motif" (SLiM) peptide sequence that confers actin-binding activity on many proteins, which they name a "short linear F-actin binding motif" (SFM). Using cryo-EM structural analysis, they resolve the SFM of full-length ITPKA bound to F-actin, confirming a previously reported structure of this peptide bound to F-actin (which corresponds to the widely-used F-actin reporter F-tractin). Guided by this result and the similar structure of Lifeact, the authors proceed to develop (what appears to this non-expert) a sophisticated bioinformatics and protein structure prediction-based pipeline to identify more proteins containing predicted SFMs in many species. Impressively, the authors then validate many of these predictions experimentally, with a mixture of cellular F-actin colocalization assays and biochemical F-actin binding assays. They furthermore develop a bioinformatic model of the sequence determinants of SFM affinity, allowing them to produce SFMs with improved actin-binding affinity through mutagenesis. They next perform evolutionary analysis which they claim supports the convergent evolution "ex-nihilo" of SFMs in many proteins, which have become fixed through purifying selection. Finally, they expand their bioinformatic analysis to identify over 100 putative SFMs in the human genome, which are found in proteins of diverse functions. Many of these SFMs have not been experimentally characterized, providing a resource that may guide future studies into the role of F-actin binding activity in many unexpected pathways.

Overall, this is largely a straightforward and conceptually appealing paper. With the exception of the evolutionary analysis (which we found a bit confusing, detailed below), the data from multiple experimental and computational threads soundly cohere to support the authors' conclusions. The identification of SFMs is likely to stimulate substantial future cell biology research. Furthermore, the authors' bioinformatic model of the SFM has the potential to enable the development of improved or fine-tuned F-actin imaging probes (similar to Lifeact or F-tractin), an idea that was not mentioned in the text. We are thus very enthusiastic about the likely impact of this paper, and we believe it has the potential to be suitable for publication in Nature Cell Biology, pending revision to address the modest concerns outlined below:

Scientific issues:

Comment 1: The presentation of the evolutionary analysis is confusing, particularly for a non-specialist reader. Why does a particular dN/dt value indicate purifying selection? How precisely did the authors come to the conclusion that the SFM motifs arose ex-nihilo? This part is particularly problematic, as there appear to be inconsistencies in the presentation. E.g. the authors write "To examine the evolutionary conservation of the SFM motif, we generated sequence logos for 12 SFM-containing proteins… These seven classes were chosen because…" (lines 205-208). However, in Figure 4a sequences logos from 9 "representative organism classes" are shown. It is unclear whether these correspond to 12 proteins across these 9 (not 7) classes, or what precisely is being depicted. This is also true for the tree shown in Figure 4c. This should be clarified. Change Figure in that way that also not experts understand it, compare with Figures already published and cite them

Answer 1: We thank the reviewer for these helpful points. We revised text and Fig. 4 (Now Fig. 6) to make the analysis better understandable.

In codon models, the nonsynonymous/synonymous mutation ratio (dN/dS) indicates evolutionary selection. For each of the 13 SFM-containing proteins, we built a codon-aware ortholog alignment and computed site-wise (per-codon) dN/dS values. We then averaged these per-codon estimates across all 13 proteins for positions within the SFM core (P1–P9) and for predefined flanking regions. For dN/dS ≈1, approximately identical numbers of nonsynonymous and synonymous mutations occur at a given site, suggesting the absence of evolutionary conservation. In contrast, dN/dS <1 indicates higher rates of synonymous mutations and/or lower rates of nonsynonymous mutations, suggesting an evolutionary sequence conservation pressure (purifying selection). Conversely, dN/dS >1 shows higher rates of nonsynonymous mutations or lower synonymous mutation rates thus indicating diversifying selection. As shown in Fig. 6c, dN/dS is consistently lower in the core compared to the flanks, supporting the hypothesis of purifying selection on the motif.

Because SLiMs are short and can evolve convergently, ex-nihilo cannot be proven strictly; we now phrase this cautiously. Our data are most consistent with ex-nihilo based on: (i) absence of conserved motif-bearing exons/modules around the SFM across distant orthologs; (ii) patchy taxonomic presence within otherwise clear orthologs (Fig. 6a); and (iii) motif age younger than gene age (motif MRCA ≪ gene MRCA). Fig. 6a legend now states that MRCA icons mark the first confident SFM appearance within a gene lineage.

Each logo represents one organism class and pools SFM instances from the 13 validated proteins that have a confidently detected SFM in that class (proteins lacking an SFM in that class do not contribute).

The manuscript was revised accordingly to make these points clearer. Please see page 13, 14.

Comment 2: In addition to facilitating the evolution of SLiMs, unstructured regions of proteins are often enriched for posttranslational modifications (PTMs). Are SFMs enriched in known or potential PTM sites? For instance, 7 out of the 8 SFMs displayed in Figure 3a contain serine and/or threonine residues, either inside the SFM or within 1 residue from the P1 / P9 boundaries, suggesting the potential for phosphorylation.

Answer 2: We agree that SLiM-rich, disordered regions often host PTMs. To test this for our SFM, we analyzed NetPhos 3.1 predictions on our SFM list and compared predefined positional categories against all other positions. We focused on Ser/Thr phosphorylation because (i) Ser/Thr residues occur frequently at or near SFM boundaries and phosphorylation is a common regulator of SLiM function (reviewed in van der Lee et al. 2014; doi: 10.1021/cr400525m) and (ii) the added phosphate introduces a defined negative charge that can modulate actin binding—as seen for cofilin Ser3 and the WH2 motif of Las17 (Moriyama et al., 1996; doi: 10.1046/j.1365-2443.1996.05005.x. ; Tyler JJ et al., 2021 doi: 10.1038/s41598-021-88826-z.). The N-terminal edge ("P—2 – P0") is enriched for high-confidence pSerine/pThreonine predictions (score ≥0.90) relative to outside positions (OR = 1.55, 95% CI 1.03–2.34; Fisher two-sided $p \approx 0.035$). In contrast, the C-terminal edge ("P10–P12") is depleted (OR = 0.18, 95% CI 0.065–0.506; $p < 0.0001$), and the actin-contacting core residues (P1/P4/P5/P8/P9) are not enriched (OR = 0.06, 95% CI 0.0036–1.001). Non-interacting core residues (P2/P3/P6/P7) are intermediate and not significant (OR = 0.66, 95% CI 0.395–1.096). Structurally, we highlight the N-terminal edge window on the complex and observe that it lies adjacent to a negatively charged patch on actin, consistent with a model in which phosphorylation at the N-terminal edge (adding negative charge) could electrostatically weaken actin binding and thereby regulate the interaction (see Extended Data Fig. 16). In line with this, our newly resolved cryo-EM structure of USP54M1 shows that Ser7 (P0) engages hydrogen bonds with Asp25 within this negatively charged actin patch (see Fig. 4g), supporting a charge-sensitive contact at the N-terminal edge. This might be interesting to follow-up in future studies. Please also see discussion page 16.

Comment 3: The cryo-EM analysis appears to be sound and well-executed. However, there are some minor issues with the cryo-EM data presentation. Specifically, the authors should show an image of the atomic model fit into the experimental cryo-EM density map, particularly highlighting the fit of the SFM. They should also, at a minimum, display half-map and map-model Fourier Shell Correlation (FSC) curves. Additionally, the EMD / PDB entries for the ITPKA-F-actin complex corresponding to the accession codes provided in the data availability statement appear to have been withdrawn. However, it seems like those in the provided validation report / Table S4 are still active (HPUB). So, this should be updated.

Answer 3: We appreciate the reviewer's suggestions and have updated the cryo-EM presentation accordingly. We now include an image of the atomic model fitted into the experimental cryo-EM density, with a zoom on the SFM helix to highlight side-chain placement. We also provide map-model Fourier Shell Correlation (FSC) curves (Extended Data Fig. 2 a and b, and Extended Data Fig. 12 a and b).

Comment 4: Several canonical F-actin binding domains with folded cores feature extended unstructured "tails" which engage the F-actin surface in a linear fashion upon binding. This includes cell adhesion proteins (e.g. vinculin and alpha-catenin) and proteins containing calponin-homology domains (CHDs), e.g. calponin, filamin, spectrin, etc. Many of these domains have been structurally resolved bound to F-actin. The Introduction and / or Discussion could be expanded to mention this as a common theme. It could also be interesting to compare / contrast the binding modes of these "tails" with the results presented here.

Answer 4: This is a very helpful suggestion. We compared the structure of the SFM with those of vinculin, alpha-catenin, L-Plastin, and Cofiin-1 (see Supplementary Table 2) and discussed the similarities and differences at page 17.

Presentation / Clarification:

Comment 5: The presentation of the predicted SFM /F-actin complexes in Figure 1 could enhanced by including a more directly visual depiction of the results. E.g., a Ca superposition of all of the complexes assigned as SFMs, versus those that were rejected (which might be shown in a supplemental figure).

Answer 5: We agree and now provide a compact, side-by-side visualization in Extended Data Fig. 5. After aligning all peptide–actin models to the ITPKA reference (panel a), we plot every model in the orientation space (Δφ, Δθ, polarity, panel b) and show the HDBSCAN-assigned clusters (panel c). We introduce a new subpanel depicting the four resulting orientation clusters mapped back onto actin (panel d) and illustrated each cluster with three representative peptide helices (panel e).

Comment 6: It would be better to show two-color fluorescence images as magenta / green rather than red / green for colorblind readers.

Answer 6: We thank the reviewer for noticing, and changed the Figure accordingly. Please see Figure 2, and Extended Data Fig. 6-9.

Comment 7: In the Methods, the "Poji" analysis would benefit from a brief explanation, rather than simply referring to a prior publication (line 592).

Answer 7: We completely agree, and explained this method more carefully. Please see page 28.

Comment 8: Lines 610 – 616, the authors refer to selecting a binding model based on the amount of remaining "free actin", but likely they mean "free ABP".

Answer 8: We thank the reviewer for noticing. This chapter has been re-written, and the sentence deleted because it was obsolete. Please see page 28

Comment 9: Lines 299-301, it would be clearer to say that SFMs "Do not need to be clustered in repeats to be necessary for F-actin targeting".

Answer 9: We agree, and replaced the sentence. Please see page 18.

Comment 10: Line 192, "Similary USP54 mutants…", this sentence is unfinished.

Answer 10: Again, we thank the reviewer for noticing and completed the sentences, please see page 10 first paragraph.

Comment 11: Line 186, "Figure 4" references should likely be "Figure 3".

Answer 11: Also, this has been corrected. Please see page 9.

Comment 12: Title and legend of Extended Data Fig. 7, "pulldown assay" should probably be "co-sedimentation assay". It would be useful to include the F-actin concentrations in this figure legend, and the SHROOM protein concentration in b.

Comment 12: This is very good point, we changed this all over the manuscript

Comment 13: A matter of personal preference, but the authors frequently use phrases such as "a lot of" and "A high number of", this could be simplified to "Many".

Answer 13: We agree, and replaced this.

Comment 14: In general, the paper would benefit (as is generally the case), from careful proofreading and ensuring that phrases have their intended meaning. As one example, line 316, "it will be very exciting to elucidate their role in actin targeting…", likely the thing of interest is what functional role actin targeting plays in these proteins, rather than vice-versa.

Answer 14: We finally carefully proofread the manuscript and ensured that phrases have their intended meaning

Reviewer #1 (Remarks on code availability):

I have not performed a detailed review of the code, as I do not have the relevant bioinformatics expertise. However, I can confirm that it is available on GitHub as described, and that the code has documentation provided which is comprehensible.

Reviewer #2:

Review: Discovery of a new evolutionarily conserved short linear F-actin binding motif.

In the article "Discovery of new evolutionarily conserved short linear F-actin binding motif" the authors report a new cryo-EM determined structure of human Inositoltriphosphate 3-kinase A (ITPKA)-F-actin complex. They perform a bioinformatics analysis of amino acid sequence in actin binding proteins and identify a new VxxLxxxFE motif present in short linear F-actin binding helical peptides.

However, the authors should address the following questions/concerns regarding the results on association of SFM-peptides to F-actin. These may require significant revision.

Comment 1: Kd values in µM range reported for Group 2 candidates indicate weak and nonspecific interactions which could also be due to the non-specific interactions of GFP. How is this ruled out?

Answer 1: This is a very good point! To exclude this, we performed a control F-actin co-sedimentation assay with EGFP alone. Here, we spun-down EGFP in absence (0 µM F-actin) and presence (12 µM F-actin) of F-actin, and the level of EGFP in the supernatant was not different. As a further control, we included the Lifeact P1/8 mutant that does not appear in the pellet fraction (please, see Extended Data Fig. 10).

In summary, EGFP alone, and the EGFP-Lifeact P1/P8 mutant do not co-sediment with F-actin after ultracentrifugation. These control experiments are now described in the method section on page 29, and the results shown in Extended Data Figure 10.

Comment 2. Can the authors report the difference in Kd values of the P1/P8 mutants?

Answer 2. This is a very good, and has been already performed by Belyy et al. 2025 for F-tractin (Figure 4). The authors found a Kd of > 100 micromolar. We discussed this result on page 15,16.

Comment 3. Difference in Kd values for the WT and M1 (triple mutant) correspond to about 1 kcal/mol difference in free energy of binding. This is within a thermal fluctuation (kBT is 0.6 kcal/mol). How is this significant?

Answer 3. Yes we agree, and calculated the $\Delta\Delta G$ for each KD value, and included this calculation in Figure 3h. According to our calculation, $\Delta\Delta G$ values > 0.6 kcal/mol are above the background, and represent differences between two samples. However, we also agree that these differences are only moderate and discussed this issue on page 9,10.

Comment 4. The authors should also study F-actin association with simultaneous mutations of P1/P8 and residues in F-actin that interact with P1/P8?

Answer 4. This is a very good idea, in particular because the amino acids inside the actin molecule interacting with the ITPKA-SFM are visible. However, there is one technical issue that we could not solve. Our first idea was to co-express mCherry actin wt and mutant with Lifeact in macrophages to analyze binding of Lifeact to F-actin. The problem here is that the endogenous actin still binds Lifeact, and it is not possible to distinguish between binding to recombinant and endogenous F-actin. An alternative approach is to express actin in bacteria, including the actin mutants. However, in this case the posttranslational modifications inside the actin molecule are missing, and therefore the data are not comparable to those obtained with native actin.

Comment 5. D-loop interactions are known to stiffen the actin filament (see, Chu and Voth, www.pnas.org/cgi/doi/10.1073/pnas.0503732102; Kang H et al. Proc Natl Acad Sci U S A. 2012 , doi: 10.1073/pnas.1211078109; Hocky GM et al. J Phys Chem B. 2016 , doi: 10.1021/acs.jpcb.6b02741). Did ITPKA binding to the D-loop stiffen the actin filament?

Answer 5. We thank the reviewer for raising this important point. To address this, we performed molecular dynamics simulations of 13-mer F-actin filaments in three conditions: actin alone (control), actin + ITPKA, and actin + USP54 M1, each simulated for 40 ns.

Interestingly, while we observed clear D-loop stabilization upon SFM binding, the global mechanical effect differs from ours and the reviewers expectation. ΔRMSF analysis (Figure 5, panel c) reveals pronounced reduction in atomic fluctuations at the D-loop region for both ITPKA and USP54 M1 binding, with negative ΔRMSF values indicating significant local stabilization compared to actin alone. Visual inspection of D-loop trajectory ensembles (Figure 5, panel e) further confirms this, showing markedly reduced conformational heterogeneity when ITPKA or USP54 M1 is bound.

However, when we analyzed global filament stiffness by calculating the persistence length (Lp) using the Chu & Voth method (Figure 5, panel b), which measures the decay of tangent-tangent correlations along the filament, we found that ITPKA and USP54 M1 binding both decreased Lp from 9.3 µm (actin alone) to 6.3 µm (ITPKA) and 5.1 µm (USP54 M1), demonstrating enhanced filament bending flexibility rather than rigidification.

This apparent paradox, local D-loop stabilization coupled with global filament softening, has been observed for other D-loop binding proteins, most notably cofilin (McCullough, Brannon R et al. 2008). Cofilin binds and locally stabilizes the D-loop while simultaneously decreasing filament persistence length. Our findings suggest that ITPKA and other SFM-containing proteins may function similarly as mechanical regulators that increase filament flexibility through constrained D-loop interactions, potentially facilitating cytoskeletal remodeling.

Comment 6: On page 8, "mutant S2 and U2" are not defined.

Answer 6: We thank the reviewer for noticing, the naming of the mutants was wrong, we corrected this. Please see page 9,10.

Comment 7: The small range of helix orientations in Fig. 1h appears to be an outcome of steric clashes with actin subunits rather than the effect of SLiM sequence conservation. This should be clarified

Answer 7: We partially agree. As shown in Extended Data Fig. 1, 2, after superposing all peptide–actin models, helices occupy a limited set of sterically accessible orientation modes around the barbed-end surface (panels b–d). Orientations outside this accessible region mostly appear as outliers, consistent with clashes or poor fits. Within the same accessible region, sequence–surface complementarity also influences which modes are populated (panel e): the hydrophobic cleft and local electrostatics align with the conserved SFM chemistry (hydrophobic P1/P4/P8, acidic P9), supporting specific placements. Consistently, in both ITPKA and USP54 M1 (Figure 4), P1/P4 bury against Ile341/Ala144/Ile345, P8 Phe π-stacks in a T-geometry with Tyr143, and P9 Glu hydrogen-bonds to Ser350, which together stabilize the observed orientations. Thus, steric accessibility and sequence complementarity jointly explain the restricted range of orientations.

Comment 8: In Group 1 SMF peptide studies, P1 is mutated from V to A, but these amino acids have very similar characteristics. The authors should study how V to D/E mutation change F-actin association?

Answer 8: We did this, and again found no co-localization of the V/F->E mutants with F-actin in macrophages, please see Extended Data Fig. 7.

Reviewer #3

(Remarks to the Author):

This work uses cryo-EM structures reported here and recently published by others of a peptide from the protein ITPKA also called "F-tractin" to search similar actin filament-binding motifs in other proteins. The authors find a remarkable number of proteins incorporating related peptides characterized by the residues Val, Leu, Phe and Glu in positions 1, 4, 8 and 9 (VxxLxxxFE). Samples of these peptides colocalized with phalloidin-stained actin filaments in cultured cells and bound to actin filaments in co-sedimentation assays. The evolutionary analysis is a strong point of the paper. These findings are interesting and likely to generate additional research of value.

On the other hand, the manuscript has weaknesses including incomplete or unclear explanations of the work, problems with the design and interpretation of some assays and missed opportunities to understand mechanisms. In particular, the authors emphasize the output of bioinformatic tools but miss many opportunities to understand the relation of structure to function, by not studying and thinking about the structures of the peptides bound to actin filaments. Additional work is required to correct these problems.

Detailed comments

Comment 1: The introduction emphasizes well characterized WH2 motifs but they are not compared structurally or functionally with the STM peptides.

Answer 1: We now provide a direct comparison in Extended Data Fig. 17. Although both contact the barbed-end cleft, the WH2 helix adopts a more tilted, opposite-polarity orientation whose N-terminal segment projects toward the D-loop and thereby creating a potential steric conflict along the actin filament, whereas the SFM engages the conserved anchors P1/P4/P8/P9 and follows a path that avoids this D-loop clash (panels b–c, d–e).

Comment 2: Line 123 following: The informatic procedures for finding actin-binding peptides related to VxxLxxxFE need to be explained more fully. For example, the paragraph does not explain the creation of atomic models of peptides bound to actin filaments or explain the question of polarity.

Answer 2: We thank the reviewer for pointing this out. We now added additional sentences explaining the SLiMfold pipeline as well as the creation of atom models of peptide-actin-complexes and the meaning of helix polarity in more detail. Please see page 6, 7. An additional Extended Data Fig. 5 was created to better illustrate our clustering and helix polarity analysis.

Comment 3: Line 129: "detailed comparison against the ITPKAArg28-Ala49–actin complex." Rather than or in addition to using this automated method, why not simply make homology models of each peptide bound to an actin filament based on the ITPKA structure? Identifying steric clashes would explain weak or no binding. Explain how "HDBSCAN" works. What is the meaning of "orientation shifts relative to ITPKA?" Do some peptides have the opposite polarity?

Answer 3. We agree that checking steric clashes and amino-acid contacts is informative. We used AlphaFold2-Multimer (AF2-M) because it provides an unbiased, high-throughput pose screen that optimizes backbone and side-chain packing rather than constraining every peptide to a single ITPKA-like placement. In contrast, templating all peptides onto the ITPKA helix assumes a fixed secondary-structure and pose, which can bias discovery toward one geometry. Moreover, template docking with a rigid actin scaffold cannot accommodate local rearrangements on the actin side that may be required for binding; such rearrangements include side-chain rotamer flips at the pocket (e.g., Actins Asp 25 side chain that flips between ITPKA-actin and USP54 M1-actin resolved structures).

HDBSCAN is a density-based clustering method that groups points which form dense regions in a space (here, orientation, polarity and RMSD to ITPKA V31-F38) while labeling sparse or inconsistent points as outliers.

After superposing each model on the ITPKA–actin reference, we describe helix orientation by two angles, Δφ (rotation around the filament/barbed-end axis) and Δθ (tilt)—plus a polarity term (+1 if the helix runs N→C like ITPKA; −1 if the orientation is inverted). Thus, 'orientation shift' denotes the pair (Δφ, Δθ) relative to the central ITPKA helix axis. Opposite-polarity peptides (helix-polarity = -1) are present; they are not parallel to ITPKA but occur at distinct tilts/rotations (Δφ, Δθ) relative to it. Extended Data Fig. 5 visualizes these orientation modes and their clustering (panels a–c) and shows representative exemplars (panels d–e).

Comment 4: Line 140: "Validation of SFM-peptides in cellulo and in vitro". In my opinion, these terms are misused. Since the invention of tissue culture a century ago, "in vitro" has been used to describe cultured cells, not biochemical and biophysical studies (also in glass) of purified proteins and other macromolecules. "In cellulo" is a vague, silly recent neologism. It does not reveal whether the cell is living or dead, in culture or in a multicellular organism. "In cells," "in vitro" and "in vivo" are clearer.

Answer 4: We agree, and changed this, please see page 7.

Comment 5: Lines 148-149. Cite the relevant figures.

Answer 5: We thank the reviewer for noticing, and cited the Figure, please see page 8, first paragraph.

Comment 6: Line 159: "Together, these data indicate that the SFM binds to F-actin with different efficiency, and preference to F-actin subpopulations." Efficiency is a thermodynamic parameter not relevant to this point. How can these peptides have preferences for different actin filament subpopulations? Do you have an hypothesis? The observation that the mutants (V→A and F→A) at positions 1 and 8 do not bind actin is obvious from the structures and the biochemical experiments. Were these mutants tested in cells to rule out nonspecific binding?

Answer 6: We agree, and deleted "efficiency" but binding to F-actin subpopulations is obvious. In particular in case of DENND1C, which mainly localized to the podosome cap (please see Figure 4). This different binding mode most likely results from preferentially binding to F-actin cross-links/branches in the podosome core, and bundles in the podosome cap. We discussed this hypothesis on page 8.

Yes, the mutants (V→A and F→A P1/P8) were tested in macrophages (Figure 2) and in H1299 cells (Extended Data Fig. 6-9), and they did not bind to F-actin.

Comment 7: Line 168, following: Actin filament binding assays. Varying the actin filament concentration is exactly what to do. However, it is impossible to measure a Kd unless the binding curves reach saturation,

which accomplished for only two peptides (CEFIP and USP54) in Fig. 3B and two USP54 peptides in Fig. 3F but not the other peptides tested. If P9E is so important for binding, why does USP54 with P9Q have a higher affinity for actin filaments than other peptides with P9E? Using either SD or SEM to describe the variation of three data points is inappropriate, since one cannot verify the assumption of normal distributions with three points; plot the three data points and the mean instead. What is the actin filament concentration used in Fig. 3E?

Answer 7: Yes, this indeed is a good point. To reach saturation in all cases we included 200 µM F-actin as highest concentration. However, for this we had to repeat alle experiments 3-fold. We excluded the PPP1R9A Kd-data because critical evaluation of our Western-blot data reveal too many non-specific bands. Since we moved last year, we now are using a different imaging system to detect the chemoluminescence signals derived from the Western-blots. In order to validate the quality of the new imaging system a calibration curve was performed and we found that all signals measured were in the linear range (see information for reviewer). Since this imager is more sensitive compared to our old one, also signals with low intensities were detected. Thus, most $K_{D,app}$ values are lower compared to our first results. However, since this was not consistently the case, we could not exclude that samples were mixed up. Thus, to be 100% sure that the right samples were measured we controlled the protein sequences by mass spectrometry.

Our new data, shown in Figure 3 b, reveal that USP54 exhibits a relatively high $K_{D,app}$, which decreases upon mutating P9Q to P9E. This indicates that P9E modulates the affinity of the SFM peptides for actin filaments. This finding is further supported by our new cryo-EM data of USP54 (see Figure 4), where we observe a polar interaction between P9E and Ser350 within the actin molecule.

Due to space limitations, we combined all $K_{D,app}$ data into a single curve presented as mean ± SD, and additionally provided the individual curves in Extended Data Figure 10.

Comment 8: Line 172: I would look at atomic models for steric interference or other problems to explain the wide range of apparent affinities for actin filaments in Fig. 4B.

Answer 8: We elucidated the cryo-EM structure of USP54-M1 and performed MD simulations (see Figure 5). We also aligned the helices of all SFMs for which $K_{D,app}$ values were determined (Figure 3A, right panel). Based on these experiments, we conclude that, in some SFMs, the amino acids at positions P4 and P9 modulate the affinity of SFM peptides for F-actin. However, modulation of SFM binding to F-actin may also arise from differences in helix formation, as observed for ESPNL, which exhibits an additional N-terminal helix (see Figure 3A, right panel). These hypotheses are discussed in detail on page 16 first paragraph.

Comment 9: Line 184, following and Fig. 3C: What is a position specific matrix? It is not clear to me that this analysis makes sense. It seems to compare apples and oranges (each protein vs other proteins) rather than comparing sequence features with properties such as affinity for actin. On the other hand the data in Fig 3C would be a good starting point to evaluate structure function relations by studying the structures to determine why some residues are more conserved than others.

Answer 9: We thank the reviewer for this thoughtful comment. The PSFM (position-specific frequency matrix) indeed compares SFM sequences to each other and highlights which motif positions are most conserved across the validated set. As clarified in the revision, our intent was not to treat the PSFM as a measure of binding strength, but to use it descriptively to guide deeper analysis.

In the revised manuscript, we have explicitly linked the PSFM to structure. We overlaid the SFM helix from ITPKA with our new cryo-EM structure of the high-affinity variant USP54-M1, both bound to actin (Figure 4 and Extended Data Fig.11). The two helices occupy the same actin pocket and superimpose with nearly identical orientation. The most conserved PSFM positions (P1, P4, P8, and P9) project into a hydrophobic groove and form matching contacts in both structures. Helical-wheel diagrams (Figure 4) show that these residues cluster on the actin-facing side of the helix, while more variable positions tend to face solvent. This provides a structural explanation for their conservation.

More subtly, the PSFM revealed a preference for polar residues at the N-terminal motif edge (P0), which led us to recognize a hydrogen bond in USP54-M1 between Ser7 and Asp25. This interaction was not evident from the ITPKA structure alone due to an alternate rotamer of Asp25, but was flagged by the PSFM. Likewise, the C-terminal isoleucine at position 18 in USP54-M1 forms a van der Waals contact with actin's Met47, another interaction not obvious from the original template, but consistent with our PSFM analysis.

In summary, the PSFM helped identify the core "anchor" residues (P1, P4, P8, P9), supported by both structure and function, and also highlighted less obvious positions (e.g., Ser7, Ile18) that contributed to interface optimization. We have revised the manuscript accordingly and believe that combining structural analysis with our PSFM-based conservation data offers a robust framework for enhancing SFM–actin interactions.

Comment 10. Line 195: cite the figure. The binding experiment with a single unspecified actin filament concentration in Fig 3E is not a valid measure of affinity. Again, the authors failed to simply look at the structures for hints about the variation in affinities for actin filaments.

Answer 10. Here, it was not our intention to analyze binding affinities but to reveal whether there are differences in principle. However, we see, the term "affinity" has been used, and it was deleted in the new version of the manuscript

Thereafter, in the next step we selected the most important (at least to our opinion) mutant, and determined the $K_{D,app}$. In the new version of the manuscript, also a new CryoEM structure of USP54 was resolved to get hinds about the structure (please see Figure 4). We carefully discussed these new insides at page 15,16.

Comment 11 Line 211: "Although a small number of substitutions were observed at these positions in certain species, these core residues remained largely unchanged, indicating strong evolutionary constraints (Figure 4a)." Again, a careful analysis of the structures should be reported to explain these observations.

Answer 11. We thank the reviewer for this suggestion. To address the link between sequence conservation and structural function, we added a new figure (Figure 4) that superimposes the ITPKA and USP54-M1 helices in complex with F-actin. The structures show that the evolutionarily conserved residues (P1, P4, P8, P9) consistently face into the actin-binding pocket and engage the same actin residues. We also included helical-wheel diagrams that visualize how these conserved side chains cluster on the actin-facing face of the helix. Together, these panels directly support the idea that the observed evolutionary constraint reflects functional necessity at the actin interface. We have updated the main text to clarify this connection.

Comment 12 Fig. 4C is unclear to me. Does the box with the MRCA's mean that an early branching example like SHROOM3 is present is all bilateria and so on up the tree? Are any of the early arriving proteins lost on higher branches? The analysis does not consider a very large part of the metazoan phylogenetic tree.

Answer 12. We thank the reviewer for the helpful comments. We have revised panel 5 and its legend to address these points. We now state explicitly in the figure legend that MRCA symbols mark the inferred first appearance of the SFM motif within each gene, not the first appearance of the gene itself. We have added a matrix, that reports presence and absence of the motif under our search and filtering criteria: green indicates that the gene contains an SFM; red indicates that the motif is absent or not detected.

An early MRCA (e.g., Bilateria) does not imply universal motif presence in descendant lineages. This is conveyed by the green/red pattern in the matrix: early-origin genes may show patchy presence, consistent with lineage-specific loss.

Figure 6A samples a fungal outgroup (Saccharomyces), a protostome exemplar (Insecta), and a broad set of vertebrate classes. As noted in the manuscript, our broad search (e.g., Cnidaria, Mollusca, Nematoda) did not yield confident motif-positive orthologs under our filters in all cases. We therefore did not find any additional motif-positive orthologs outside the clades shown.

Comment 13: Line 247: "they are involved in the regulation of diverse cellular functions (Figure 5b, Extended Data Figure 10 and Table 1)." The word "involved" in might be justified but how do you know they have "regulatory functions?" "Modulate actin" is vague. Some of these proteins have been characterized, revealing in most cases rather specific mechanisms of action. It is hard to justify lumping them in broad categories using rough genome annotations.

Answer 13: Yes, we agree, but we did not use genome annotations to categorize the proteins. Instead, we categorized them based on the functions reported in the literature. In the revised version of the manuscript, we have included a glossary that briefly summarizes the cellular roles of the newly identified SFM proteins. Our intention is to provide at least a concise overview of the current knowledge regarding these newly identified SFM proteins. Please, see page 17,18, and Table 2.

Comment 14: Line 263: "discovery of a new short linear F-actin binding motif (SFM), and provide first evidence for its biological role" is an overstatement. The VxxLxxxFE was discovered and exploited by one of the authors to make the actin filament binding peptide Lifeact. I do not understand how this paper explains the biological roles of VxxLxxxFE other than grouping the proteins in broad and possibly incorrect categories.

Answer 14: We thank the reviewer for this important comment and have revised the manuscript to clarify both the novelty of the motif and its functional relevance. We respectfully disagree with the assertion that the short F-actin binding motif was already known through Lifeact. Lifeact was derived from the N-terminal segment of yeast Abp140 and, as stated in the original publication, was thought to lack homologous sequences in higher eukaryotes: "Lifeact has no homologous sequences in higher eukaryotes, which makes competition with endogenous proteins less likely." Our work revises this view by identifying over 120 eukaryotic proteins-across multiple lineages-that harbor a conserved motif with the same geometry, orientation, and actin-binding interface. These instances are confirmed through structural, biochemical, and evolutionary evidence, including newly resolved cryo-EM structures and mutational validation. Our results generalize the Lifeact-derived sequence into a conserved F-actin-binding motif (SFM) with a common structure and interface across diverse proteins.

Regarding the biological role, while the current study does not include new cellular data, we bring together compelling functional insights from prior publications. For example, deletion of the tandem motif in the Rho-GEF PLEKHG3 impairs cell polarity and destabilizes leading-edge protrusions (Nguyen et al., PNAS). Similarly, removal of the SFM in ARHGEF11 disrupts the Rac/Rho signaling balance, leading to defects in front-back migration (Nanda et al., Nat. Commun.). In ITPKA, mutations within the motif reduce Ins(1,4,5)P_3 kinase activity (Ashour et al.), and impairs the formation of invasive protrusions in cancer cells (Küster et al.). These examples demonstrate that the motif is not only structurally conserved but also biologically relevant across diverse systems.

Thus, by defining the SFM, our study brings together prior observations and frames a recurring actin-regulatory module, as evidenced by published work on migration, signaling, and invasion. The manuscript was modified accordingly.

Comment 15: Lines 277-321: This discussion of possible functions of SFM motifs is mostly generalizations regarding localization to cellular compartments and "modulation of actin" rather than considering the more interesting question of actual mechanisms.

Answer 15: We addressed this concern on page 17,18.

Comment 16: Line 512: "For the Phalloidin-stabilized F-actin-ITPKA complex, we initially applied 2 µl of 1 µM Phalloidin-stabilized F-actin to the grids. Subsequently, we added 2 µl of 10 µM ITPKA and mixed it directly on the grids immediately before plunge-freezing in a liquid ethane/propane." Given the low affinity of ITPKA for actin, how did this procedure saturate the filaments with the ligand?

Answer 16. The reason we limited the ITPKA concentration to only a tenfold excess is that wild-type ITPKA exhibits a strong propensity to bundle F-actin. Even when directly mixed with F-actin on the EM grids at a 1:10 actin:ITPKA molar ratio, we observed extensive filament bundling. Such bundles are not suitable for cryo-EM analysis, which necessitated the use of a lower ligand concentration. We agree with the reviewer that the F-actin filaments in our preparations are not fully saturated with ITPKA. This is evident from the cryo-EM reconstructions, where the density corresponding to the ITPKA SFM region is noticeably weaker than that of the F-actin filament. This observation is consistent with the relatively low binding affinity of ITPKA for actin. To address this limitation, we performed extensive 3D classification, including focused 3D classification with a mask at the ITPKA SFM region without alignment, to enrich for F-actin segments bound to ITPKA. While these procedures yielded a final EM density map that clearly resolves ITPKA in complex with F-actin, the densities at the ITPKA binding site remain weaker than those of the actin filament, further reflecting the partial occupancy.

Comment 17. Line 1242: Explain the meaning of "plotting mean ipTM."

Answer 17. We thank the reviewer. The manuscript was modified accordingly. Please see page 6,7.

Comment 18: Line 1248: Add a phrase to explain "HDBSCAN clustering."

Answer 18: We thank the reviewer. The manuscript was modified accordingly. Please see page 7.

Comment 19: Line 1263: Add a phrase to explain "Poji radial profiles."

Answer 19: We did this, please see page 28.

Comment 20: Line 1265: "highest F-actin intensity" should be "highest fluorescent phalloidin intensity."

Answer 20: We did this, please see Figure 2.

Comment 21: Line 1084: Add a phrase to explain "position specific matrix (PSFM)."

Answer 21: We thank the reviewer. The manuscript was modified accordingly. Please see page 29,30.

Comment 22: Line 1105: Add a phrase to explain "dN/dS ratio."

Answer 22: We revised text and Fig. 4 to make the analysis better understandable.

Comment 23: Fig. 1A: P4 does not look like leucine from the angle shown. Showing the map for the new structure would be informative Showing a stick figure of the peptide on a space filling model of actin would illustrate how the conserved residues interact with actin and residues on the other face of the helix that do not interact with actin. Some of the renderings in Extended Data Fig 1 are close to what is needed but do not show the binding sites of the four crucial residues. Helical wheels may assist in understanding evolutional constraints.

Answer 23: We thank the reviewer for the helpful suggestions. To clarify the identity and context of the conserved motif residues, we generated a new Figure 4 that presents several updated structural views. First, we show the SFM peptide in stick representation overlaid on a space-filling model of actin, which illustrates how the key conserved residues (P1, P4, P8, P9) insert into the actin-binding groove, while residues on the opposite face of the helix remain solvent-exposed. Second, we include zoomed-in views of the ITPKA and

USP54-M1 peptides superimposed in the actin pocket, showing in detail how these conserved residues form contacts across both structures. Finally, we added helical-wheel diagrams for ITPKA and USP54-M1, which clearly visualize the conserved residues cluster on the actin-facing side of the helix.

Comment 24: What is the significance of the sentences in red font?

Answer 24: We apologize, this was a mistake.

Comment 25: I recommend using actin filaments and actin monomers. G- and F-actin are jargon.

Answer 25: We agree and have revised this throughout the manuscript.

Comment 26: Line 54: "Many ABPs are inhibited in resting cells.." "Some" and "inactive" are better.

Answer 26: This has been changes, please see page 4.

Comment 27: Line 70: "...thereby modulating polymerisation and filament dynamics." This is vague and not informative about what is known about the mechanisms of action.

Answer 27: Yes, we agree, and described this in more detail. Please see page 4,5.

Sincerely,

[redacted]

Sabine Windhorst

Zentrum für Experimentelle Medizin

Institut für Biochemie und Signaltransduktion (IBS)

HAMBURG

Prof. Dr. Sabine Windhorst

Martinistraße 52
20246 Hamburg

Gebäude N25, EG, Raum 00.1.011
Telefon: +49 (0) 40 7410-56013
Fax: +49 (0) 40 7410-56818
s.windhorst@uke.de
www.uke.de

Universitätsklinikum Hamburg-Eppendorf | Martinistraße 52 | 20246 Hamburg

Zentrum für Experimentelle Medizin – Institut für Biochemie und Signaltransduktion

Nature Cell Biology

Hamburg, 10.02.2026

Second Re-submission of the manuscript "Discovery of a new evolutionarily conserved short linear actin filament binding motif"

Dear Dr. Verzosa David,

We thank you and the reviewers for the careful evaluation of our revised manuscript entitled *"Discovery of a new evolutionarily conserved short linear actin filament binding motif"* and for the constructive and helpful comments.

All points raised by the reviewer, as summarized by the Editor, have been carefully addressed. The corresponding revisions have been implemented in the manuscript and are highlighted in yellow for clarity.

Below, we provide a detailed, point-by-point response to each comment, describing the changes made and clarifying how the concerns were addressed.

Summary of the Editor:

A) Compare D-loop structures in 7AD9 with the ensemble of conformations (as suggested by Reviewer #2 to address Reviewer #1's concerns)

B) additional discussion on potential future directions regarding potential effects of filament softening (Reviewer #2 to address Reviewer #1's concerns)

Gerichtsstand: Hamburg
Körperschaft des öffentlichen Rechts
USt-Id: DE 21 8618 948

Bank: Hamburger Sparkasse AG
BIC: HASP DEHH | Konto: 1280 1706 38
IBAN: DE91 2005 0550 1280 1706 38

Vorstandsmitglieder:
Prof. Dr. Christian Gerloff (Vorstandsvorsitzender)
Joachim Prölß
Prof. Dr. Blanche Schwappach-Pignataro
Corinna Wriedt

Universitätsklinikum Hamburg-Eppendorf

C) Provide additional explanations and parameters of the MD simulations, and compare with Iyer et al (doi: 10.1073/pnas.2421467122) (see comments from Reviewer #3)

D) All other referee concerns pertaining to strengthening existing data, providing controls, methodological details, clarifications and textual changes, should also be addressed.

E) Finally please pay close attention to our guidelines on statistical and methodological reporting (listed below) as failure to do so may delay the reconsideration of the revised manuscript.In particular please provide:

- a Supplementary Figure including unprocessed images of all gels/blots in the form of a multi-page pdf file. Please ensure that blots/gels are labeled and the sections presented in the figures are clearly indicated.

- a Supplementary Table including all numerical source data in Excel format, with data for different figures provided as different sheets within a single Excel file. The file should include source data giving rise to graphical representations and statistical descriptions in the paper and for all instances where the figures present representative experiments of multiple independent repeats, the source data of all repeats should be provided.

In contrast, although we agree with referee #1 that additional experimentation to justify MD simulations regarding alterations in actin stiffness would provide valuable insights, we consider this point to be beyond the scope of the present study. Thus, addressing it experimentally will not be necessary for reconsideration of the manuscript at this journal but would welcome further discussion and caveats as appropriate.

We therefore invite you to take these points into account when revising the manuscript. In addition, when preparing the revision please:

- ensure that it conforms to our format instructions and publication policies (see below and www.nature.com/nature/authors/).

- provide a point-by-point rebuttal to the full referee reports verbatim, as provided at the end of this letter.
- for any revision that includes light microscopy data, we ask our authors to please include a completed light microscopy reporting table https://www.nature.com/documents/Light_microscopy_reporting_table.xlsx to ensure the methods are described thoroughly. The table will be available to reviewers and ultimately published should the manuscript be accepted at the journal.

Reviewer #1
In their revised manuscript, Paraschiakos and colleagues were highly responsive to our comments and those of the other reviewers. Many aspects of the paper were substantively improved, including rigorous presentation of the structural data, and a much more comprehensible discussion of the evolutionary implications of the work. The inclusion of the phosphosite analysis and comparison with other actin-bound structures also fills out the paper nicely.

Comment 1: There is, however, one new area of major concern. In response to a reviewer comment, the authors have now included analysis of molecular dynamics simulations (MD) suggesting that SFMs impact the bending stiffness of actin filaments. This is a quite striking and potentially very important result, but it is in our opinion weakly supported. We believe rigorously making this claim would require experimental support, which could be garnered through either fluorescence microscopy experiments in the presence / absence of SFM (e.g. as done in ref. 50 for cofilin) or by analyzing the curvature of filament segments in cryo-EM images (as done in ref. 48) and comparing them to bare filaments. Both of these would be non-trivial exercises.

A major reason for this skepticism is the potential for the MD simulations to be sensitive to the starting conformation of the D loop, which as the authors note has previously been linked to actin bending dynamics. The D loop tends to be the most poorly resolved portion of actin in cryo-EM reconstructions, which has been characterized through extensive local masked classification to represent conformational variability in prior studies. Inspecting Fig. 1a, it is apparent that the authors built the D loop of the ITPKA structure in a different conformation than that of the Lifeact structure (7AD9). Examining Fig. 5e, it appears that the short region of the D loop displaying rigidification in the presence of SFMs is within the stretch that the authors modeled as a different conformation. However, the model-map fit shown in ED Fig. 1a shows poor density for this region of the D loop, suggesting that multiple conformations could potentially be consistent with the density.
To at least moderately enhance confidence in the existing approach, the authors could test the robustness of their analysis by performing simulations with different starting D loop conformations (e.g. that in the Lifeact structure).

A path forward that would satisfy us is to simply remove the MD analysis / claims about effects on actin mechanics. We believe it is is ancillary to the overall findings of the paper, and it could be the subject of a future study featuring an experimental component as outlined above.-> das wollte aber reviewer 2 However, we realize this may put us at odds with other reviewers, so we encourage the editor to weigh in on this point.

Answer 1: To answer the reviewer's question regarding the "bare actin structure," we generated the actin-only control by computationally removing the peptide from our ITPKA-bound Cryo-EM reconstruction. For the peptide-bound systems (ITPKA and USP54), simulations were initiated from their respective experimental structures to ensure the most accurate representation of each specific peptide-protein interface. While this means the starting coordinates differed slightly, the highly conserved actin core allows all systems to rapidly relax into a comparable structural baseline during equilibration. Regarding the D-loop conformation, we agree with Reviewer #2 that MD naturally samples a broad thermal ensemble at 310 K, moving beyond the static starting snapshot. To demonstrate this robustness, we compared our trajectories to the Lifeact-bound structure (PDB 7AD9) and found that our simulations spontaneously sample D-loop conformations compatible with 7AD9 (***please see Extended Data Fig. 13d-e).*** This confirms that our results are not artifacts of the starting model but reflect valid conformational dynamics.

Reviewer #2

I am happy with the changes made. I recommend publication

Reviewer #2's further comments on Reviewer #1's current concerns:
The major skepticism of the MD results expressed by the reviewer arises from (1) the difference in D-loop conformation modeled in the ITPKA-bound filament compared to the Lifeact-bound filament structure (7AD9) used as a template, and (2) the lack of experimental results directly supporting filament softening due to ITPKA interactions with the D-loop.

Comment 1: To address the first concern, I suggest that the authors explicitly compare the D-loop structure in 7AD9 with the ensemble of D-loop conformations rendered in Figure 5e. I believe that at least one of the simulated conformations would match well with the D-loop conformation observed in 7AD9. The reviewer's concern appears to be influenced by reliance on a single static conformation derived from cryo-EM reconstruction, whereas MD simulations—although initiated from a single structure—naturally generate an ensemble of thermally accessible D-loop conformations (at 310K!). In this context, the modest difference in D-loop conformation between the starting structure and 7AD9 does not represent a significant limitation of the simulations.

Regarding the second concern—the lack of direct experimental evidence supporting filament softening due to ITPKA interactions with the D-loop—I emphasize that the role of MD simulations here is not to replace experiments, but to provide mechanistic insight and testable predictions. The authors should

include a line in the text about this while discussing the MD results. Within the context of prior experimental and computational studies that have established a link between D-loop flexibility and filament bending rigidity, the MD results presented are mechanically well grounded and internally consistent.

Answer 1: We thank the reviewer for raising the point regarding the D-loop conformation in PDB 7AD9. We would like to emphasize that 7AD9 represents a single static cryo-EM snapshot (in a Life-Act-bound state), whereas the actin D-loop is intrinsically dynamic and MD simulations naturally sample an ensemble of thermally accessible conformations. Importantly, cryo-EM reconstructions reflect an averaged conformation, and the D-loop is among the most flexible regions of actin, with less well-defined density than the actin core. To directly address this concern, we now explicitly compare our simulated D-loop conformations to 7AD9 by aligning each actin subunit to 7AD9 using only core Cα atoms (excluding the D-loop) and quantifying the D-loop Cα RMSD (residues 46-50) over time (please see ***Extended Data Fig. 13d and e***). Notably, this analysis shows that both SFM-bound systems (ITPKA and USP54 M1) exhibit a slightly reduced D-loop deviation from the 7AD9 reference compared to actin alone (please see mean RMSD in ***Extended Data Fig. 13d***), indicating that in average the simulations not only sample 7AD9-compatible conformations but that SFM binding tends to stabilize a D-loop state closer to the 7AD9 conformation. Please see also ***discussion on page 18,19.***

Reviewer #3

Windhorst et al. revised

The authors carried out additional work and made extensive revisions in response to the first round of review. I am generally satisfied with the constructive responses to my review. The new work provides valuable additional information about the interactions of SFM with actin filaments. However, some of the extensive new sections (conveniently in red font) are still rough and need revision. I am now enthusiastic about the publication of this paper after suitable revision.

Comment 1: Line 2: “F-actin” not replaced with “actin filaments” as promised. Many other examples are found throughout the text.

Answer 1: We thank the reviewer for noticing and replaced “F-actin” with “actin filaments” and “G-actin” with globular actin.

Comment 2: Line 255: “insides” should be “insights.”

Answer 2: Again, we thank the reviewer for noticing and corrected this. Please see ***page 21.***

Comment 3: Line 295: “(Figure 4c)” should be “(Figure 5b).”

Answer 3: This has been corrected.

Comment 4: Order of text: The results might be easier to understand if excellent new Fig 4 and the associated text were presented before Fig 3 and its text.

Answer 4: In principle we agree but the USP54 M1 mutant first appear in Fig. 3, and its new Cryo-EM structure is shown in Fig.4. That is why the MD-simulation of the USP54 M1 binding to actin filaments first can be shown in Fig. 5.

Comment 5: Fig. 4: These structure renderings are an important addition, but many of the labels are difficult to read, particularly the purple font on the space-filling model. Enlarge the labels for the subunits in panels a and b as “Subunit A0” and “Subunit A-2.” Consider showing panels a and b as stereo pairs, which would be more informative. Alternatively, a supplemental movie might help. The ribbon diagrams in panels D-F would be better with darker colors. The blue sidechain labels are too faint. Label panels g and h with larger and darker “ITPKA” and “USP54 M1”. The helical wheels are helpful, but labels are essential to identify the upper residues as part of actin.

Answer 5: We thank the reviewer for the helpful suggestions to improve the clarity of ***Figure 4.*** We have revised the figure significantly to enhance readability. Specifically, we enlarged the labels for "Subunit A0" and "Subunit A-2" in panels a and b, and we added a background box to the text to ensure high contrast against the space-filling model. Furthermore, we darkened the colors of the ribbon diagrams in panels a-f and increased the contrast of the sidechain labels. Finally, we enlarged the labels in panels g and h and annotated the helical wheels to explicitly identify the actin residues. As suggested, we have also generated a supplemental movie (***Movie S1***) to better illustrate the 3D spatial arrangement.

Comment 6: Line 281: Give the temperature of the simulations.

Answer 6: 310 Kelvin, please see ***Methods page 12***.

Comment 7: Fig. 5: The MD simulations and their analysis are another valuable addition. However, the most of the panels and labels are too small, especially panel g. The plots in Panel c clearly show SFM binding reduces D-loop fluctuations, but these differences are not apparent to my eye in panel e. The D-loop region of panel C should be shown at larger size (expanded region of x-axis) in an inset to show which residues are affected. Neither of these panels shows how SFM binding affects interactions between the side chains of D-loop residues with the adjacent actin subunit. Panel f needs a y-axis label "Residue interactions along the long pitch helix." The legend for panel f must explain that the x-axis labels are pairs of interacting residues in subunits along the long-pitch helix with the lower subunit first and upper residue second. Oddly, the most important D-loop residue making long-pitch interactions, M44, is not considered. Neither is V43.

Answer 7: We thank the reviewer for these constructive suggestions and agree that improving readability and clarity strengthens ***Fig. 5***.

To address the visibility issues, we enlarged all panels and labels, in particular panel g. In addition, because the reduction in D-loop fluctuations is clearly captured in panel c but is less apparent by eye in panel e, we added an expanded inset of the D-loop region (zoomed x-axis) to highlight the affected residues. We also improved the D-loop visualization by aligning trajectories on the actin core to minimize global motion, overlaying fewer representative frames, and annotating key D-loop residues (G42, M47, G48, Q49) to emphasize the reduced conformational spread. Also, we changed the orders of Figures: Figure C is now D, and Figure D is C.

Regarding long-pitch inter-subunit interactions, we would like to clarify that panel f already reports residue-residue interactions between actin protomers along the long-pitch helix (subunit i contacting subunit i-2). As suggested, we have added the y-axis label "Residue interactions along the long pitch helix" in ***Figure 5f,*** and we revised the legend to explicitly explain that the y-axis lists pairs of interacting residues between the two long-pitch related subunits, with the lower subunit residue given first and the upper subunit residue second.

Finally, we agree that V43 and M44 are central D-loop residues in long-pitch contacts. In our analysis, however, their interaction changes upon SFM binding were not among the largest contact frequency differences, and ***panel f*** was intentionally designed to highlight the most significant interaction changes. We have therefore kept panel f focused on the strongest differences, and we now clarify this selection criterion in the legend to avoid confusion.

Comment 8: Line 304: what are "inter-subunit contact edges?"

Answer 8: By "inter-subunit contact edges" we mean residue-residue contacts between neighboring actin protomers along the long-pitch helix (subunit i contacting subunit i-2). We have clarified this wording in the manuscript. Please see ***page 11.***

Comment 9: Line 306: the text says "both peptides consistently reduced the inter-protomer contact of residues Q49–E167," but Fig 5F shows this is largely restricted to the first 15 ns of the simulation. Explain this more clearly. Might this be due to the shifting conformations of the actin subunits during the initial part of the simulation, as noted in the next paragraph?

Answer 9: We thank the reviewer for pointing out that the time-dependence of inter-protomer contact changes needs to be clarified in more detail. We agree that the reduction of Q49-E167 contact

occupancy is most pronounced during the initial 15 ns of the simulation (***Fig. 5F***). Notably, our reported values (ITPKA – 22 %, USP54 M1 – 26 %, Δ contact occupancy vs actin alone, in percentage points) represent time-averaged contact occupancies relative to actin alone. The early phase of the MD simulation thus contributes disproportionally to these time-averaged values.
To analyze this in more detail, we performed dihedral angle analysis of the actin subunit SD2-SD1-SD3-SD4 dihedral angle on our MD simulations as described by Iyer et al (PNAS, 2025) and found that all three studied systems (actin alone, actin+ITPKA, actin+USP54-M1) undergo a transition towards a more twisted state during 40 ns, consistent with recent findings published by Iyer et al (PNAS, 2025). Our cryo-EM-derived starting structures show SD2-SD1-SD3-SD4 dihedral angles in the range of -3° to -4° and reach dihedral angles in the range of -8° to -9° for actin alone and actin+USP54-M1 while actin+ITPKA exhibits a less pronounced twisting with dihedral angles fluctuating around -6.5° after 40 ns (see ***Fig. S13f).***
This suggests that the observed conformational shift as seen by this dihedral angle analysis does not explain the Q49-E167 contact occupancy differences observed between actin alone and actin+USP54-M1 (***Fig. 5f),*** because both systems show a comparable structural transition towards a more twisted state, keeping the Q49-E167 contact mostly intact in the actin control, while the same contact appears less frequent in the presence of the USP54-M1 peptide.
We thus hypothesize that SFM-binding-mediated competitive occlusion at the D-loop tip favors a filament state in which intra-protomer contacts occur more frequently.

Comment 10: Comment on MD simulations: The authors would not have been aware of a recent paper by Iyer et al. (PNAS Dec 2025), but may want to take their findings into consideration during final revision. Iyer et al. also simulated a 13-mer Mg-ADP-actin filament at 310 K and found that the conformations of the subunits and the helical twist fluctuate on a nanosecond time scale, only equilibrating after about 200 ns to a different structure than the starting cryo-EM structure. Did you observe these fluctuations in subunit conformations in your MD simulations of the actin filament alone or with SFM peptides? Did the fluctuations influence SFM peptide binding or longitudinal interactions between the subunits? Might the fact that your simulations were during the transition between the frozen and warm state have accounted for the variability over time in Fig. 5F?

Answer 10: We thank the reviewer for pointing out the highly relevant study by Iyer et al (PNAS, 2025) on structural dynamics of actin filaments. We applied the actin subunit definitions and SD2-SD1-SD3-SD4 dihedral angle analysis shown in their paper to our MD simulations and found that all our systems (actin alone, actin+ITPKA, actin+USP54-M1) indeed show the same expected equilibration trend towards a more twisted state as explained by Iyer et al (***Fig. S13f).*** However, while following the same trend with very similar dihedral angles as reported by Iyer et al, we note that our simulations are considerably shorter and thus do not fully resemble the finally equilibrated warm state. Nonetheless, we do observe nanosecond subunit conformational fluctuations in our MD simulations and found that the ITPKA peptide seems to bias the filament equilibration towards less negative dihedral angles (i.e. towards a less twisted state) while the USP54-M1 decorated actin filament behaved more like an undecorated (control) filament with comparable dihedral angle changes over time (***Fig. S13f).*** Comparing our undecorated actin filament with the USP54-M1 bound filament, we therefore do not see an apparent correlation between subunit conformational fluctuations and our observed changes in longitudinal inter-protomer interactions during the 40 ns timescale (see also ***Fig. 5f).*** Although the undecorated control filament and the USP54-M1 bound filament showed comparable dihedral angle changes over time, the USP54-M1 (and ITPKA) peptide remained stably bound to the actin filament throughout the 40 ns trajectory (***Fig. 5a),*** indicating that conformational fluctuations do not influence SFM peptide binding within 40 ns. Conversely, SFM peptide binding might influence filament equilibration and subunit conformational fluctuations as seen for the ITPKA peptide. More extensive MD simulations over substantially longer time periods might be necessary though to quantify such a potential effect. We have added the study by Iyer et al to our discussion.

Comment 11: Lines 311-314: Confusing. Which actin subunits have these changes? Between i+1 or i-1? Spell this out for each pair in the legend. What do the percentages mean?

Answer 11: Thank you for pointing this out. We agree the description needed to be more explicit. All residue pairs reported in lines 311-314 refer to inter-subunit contacts between long-pitch neighboring

actin protomers, i.e. subunit i interacting with subunit i-2. We now state this explicitly in the figure legend and text. Please see ***Figure 5f***

The percent values report the time averaged change in contact occupancy relative to actin alone. For each residue pair, we computed the contact occupancy as the fraction of MD frames in which the two residues are in contact (across all protomers), and then calculated the difference between conditions.

This difference is reported as percentage points (fraction × 100). Thus, a value of e.g. −30% indicates that the contact occurs in 30 percentage points fewer frames in the SFM-bound simulation compared to actin alone across the whole filament.

Comment 12: Line 456: replace "share identical interactions within.." with "interact with the same residues in.."

Answer 12: This has been done, please ***see page 18.***

Comment 13: Line 523: delete "strongly." This is an inappropriate construction.
Answer 13: We agree, and deleted "Strongly"

Comment 14: Style: The text is clear, but many sentences could be more concise. For example on Line 242 "Our data depicted in Figure 3e – h reveal that the M1 mutants of USP54 and SHROOM3 exhibit increased binding strength to actin filaments compared to their wild-type counterparts" could be "The affinities of M1 mutants of USP54 and SHROOM3 for actin filaments are higher than their wild-type counterparts (Figure 3e)." Many other sentences could be more concise.

Answer 14: We replaced this sentence, and carefully controlled the text again.

Comment 15: Some figure legends such as 5 are too long. Just explain the panels and leave the interpretation to the text as done in Figure 4.

Answer 15: In the new version of the manuscript all Figure Legends are shortened.

Signed, Tom Pollard

Sincerely,

[redacted]

Sabine Windhorst
